# Housing and child health in sub-Saharan Africa: A cross-sectional analysis

**DOI:** 10.1371/journal.pmed.1003055

**Published:** 2020-03-23

**Authors:** Lucy S. Tusting, Peter W. Gething, Harry S. Gibson, Brian Greenwood, Jakob Knudsen, Steve W. Lindsay, Samir Bhatt

**Affiliations:** 1 Department of Disease Control, London School of Hygiene & Tropical Medicine, London, United Kingdom; 2 Big Data Institute, Nuffield Department of Medicine, University of Oxford, United Kingdom; 3 The Royal Danish Academy of Fine Arts, Schools of Architecture, Design and Conservation, Copenhagen, Denmark; 4 Department of Biosciences, Durham University, Durham, United Kingdom; 5 Department of Infectious Disease Epidemiology, Imperial College London, London, United Kingdom; Umeå Centre for Global Health Research, Umeå University, SWEDEN

## Abstract

**Background:**

Housing is essential to human well-being but neglected in global health. Today, housing in Africa is rapidly improving alongside economic development, creating an urgent need to understand how these changes can benefit health. We hypothesised that improved housing is associated with better health in children living in sub-Saharan Africa (SSA). We conducted a cross-sectional analysis of housing conditions relative to a range of child health outcomes in SSA.

**Methods and findings:**

Cross-sectional data were analysed for 824,694 children surveyed in 54 Demographic and Health Surveys, 21 Malaria Indicator Surveys, and two AIDS Indicator Surveys conducted in 33 countries between 2001 and 2017 that measured malaria infection by microscopy or rapid diagnostic test (RDT), diarrhoea, acute respiratory infections (ARIs), stunting, wasting, underweight, or anaemia in children aged 0–5 years. The mean age of children was 2.5 years, and 49.7% were female. Housing was categorised into a binary variable based on a United Nations definition comparing improved housing (with improved drinking water, improved sanitation, sufficient living area, and finished building materials) versus unimproved housing (all other houses). Associations between house type and child health outcomes were determined using conditional logistic regression within surveys, adjusting for prespecified covariables including age, sex, household wealth, insecticide-treated bed net use, and vaccination status. Individual survey odds ratios (ORs) were pooled using random-effects meta-analysis. Across surveys, improved housing was associated with 8%–18% lower odds of all outcomes except ARI (malaria infection by microscopy: adjusted OR [aOR] 0.88, 95% confidence intervals [CIs] 0.80–0.97, *p* = 0.01; malaria infection by RDT: aOR 0.82, 95% CI 0.77–0.88, *p* < 0.001; diarrhoea: aOR 0.92, 95% CI 0.88–0.97, *p* = 0.001; ARI: aOR 0.96, 95% CI 0.87–1.07, *p* = 0.49; stunting: aOR 0.83, 95% CI 0.77–0.88, *p* < 0.001; wasting: aOR 0.90, 95% CI 0.83–0.99, *p* = 0.03; underweight: aOR 0.85, 95% CI 0.80–0.90, *p* < 0.001; any anaemia: aOR 0.87, 95% CI 0.82–0.92, *p* < 0.001; severe anaemia: aOR 0.89, 95% CI 0.84–0.95, *p* < 0.001). In comparison, insecticide-treated net use was associated with 16%–17% lower odds of malaria infection (microscopy: aOR 0.83, 95% CI 0.78–0.88, *p* < 0.001; RDT: aOR 0.84, 95% CI 0.79–0.88, *p* < 0.001). Drinking water source and sanitation facility alone were not associated with diarrhoea. The main study limitations are the use of self-reported diarrhoea and ARI, as well as potential residual confounding by socioeconomic position, despite adjustments for household wealth and education.

**Conclusions:**

In this study, we observed that poor housing, which includes inadequate drinking water and sanitation facility, is associated with health outcomes known to increase child mortality in SSA. Improvements to housing may be protective against a number of important childhood infectious diseases as well as poor growth outcomes, with major potential to improve children’s health and survival across SSA.

## Abstract 2

In a cross-sectional analysis, Lucy S. Tusting and colleagues investigate housing conditions and child health outcomes in sub-Saharan Africa.

## Author summary

**Why was this study done?:**

- Children’s health has improved substantially in sub-Saharan Africa since 2000, but mortality among children under 5 years old is still 14 times higher than among children living in developed regions.
- Adequate housing is essential to human well-being but is neglected in public health.
- Today African housing is rapidly changing alongside economic development, presenting a major opportunity for health. However, to realise this potential, we urgently need to understand better how housing conditions affect children’s health.

**What did the researchers do and find?:**

- We assessed whether improved housing is associated with a lower risk of five major causes of death in African children: malaria, diarrhoea, acute respiratory infection, growth failure, and anaemia.
- We performed a cross-sectional analysis using data from 33 African countries collected in 77 national surveys conducted between 2001 and 2017. We analysed data from 824,694 children aged 0–5 years living in 468,471 households.
- Children living in housing with improved drinking water and sanitation, sufficient living area, and durable construction had a reduced likelihood of four major causes of death: malaria (12%–18% reduction), diarrhoea (8% reduction), growth failure (stunting: 17% reduction, wasting: 10% reduction, underweight: 15% reduction), and anaemia (11%–13% reduction) after accounting for differences in levels of urbanisation, poverty, vaccinations, and other public health interventions. However, there was no detectable relationship between housing conditions and acute respiratory infection.
- As a comparison with mainstream health interventions, children sleeping under insecticide-treated bed nets were 16%–17% less likely to be infected with malaria compared with children not sleeping under bed nets, but the quality of drinking water and sanitation alone were not associated with any reduction in diarrhoea.

**What do these findings mean?:**

- Improvements to housing may help to reduce the burden of disease among children in sub-Saharan Africa alongside wider poverty reduction.
- Housing improvements may have a similar effect on malaria to insecticide-treated bed nets.
- Community-level experimental studies are needed to replicate our findings in different African settings.
- The main limitation of this study was the use of observational data, which means that it is not possible to fully account for factors that may confound the relationship between housing and health, such as household wealth.

## Introduction

Children’s health has improved substantially in sub-Saharan Africa (SSA) since 2000, with reductions in the leading causes of death, including diarrhoea, malaria, and pneumonia [1]. However, mortality in children under 5 years old in SSA remains the highest globally, and at least 34 countries in this region are unlikely to meet the United Nations (UN) Sustainable Development Goals target of <25 deaths per 1,000 live births by 2030 [2]. Childhood malnutrition, which contributes to 45% of deaths in young children, is not expected to disappear from any African country before 2030 [3]. It is widely recognised that child health is linked to poverty-related factors such as water, sanitation, and education, so substantial reductions in child mortality are best achieved through an integrated approach that addresses health alongside wider development goals [4].

Provision of adequate housing, a basic human right and central to human well-being, remains neglected in global health, although features of substandard housing such as an unsafe water supply, poor sanitation, indoor air pollution, and overcrowding have long been recognised as risk factors for diseases such as acute respiratory infection (ARI), diarrhoea, malaria, and tuberculosis [5, 6]. Indeed, improving slum housing was a cornerstone of early disease control campaigns in Europe and North America [7–9]. Today, housing remains highly relevant to child health in SSA. For example, house design influences malaria transmission because the primary African malaria vector, *Anopheles gambiae*, typically bites indoors at nighttime. Simple modifications such as screening windows and doors can therefore reduce exposure to malaria infection [10]. Despite these links, housing is poorly addressed in public health. For example, the World Health Organization’s 2018 guidelines on housing and health do not mention malaria [11].

**Fig 1:**
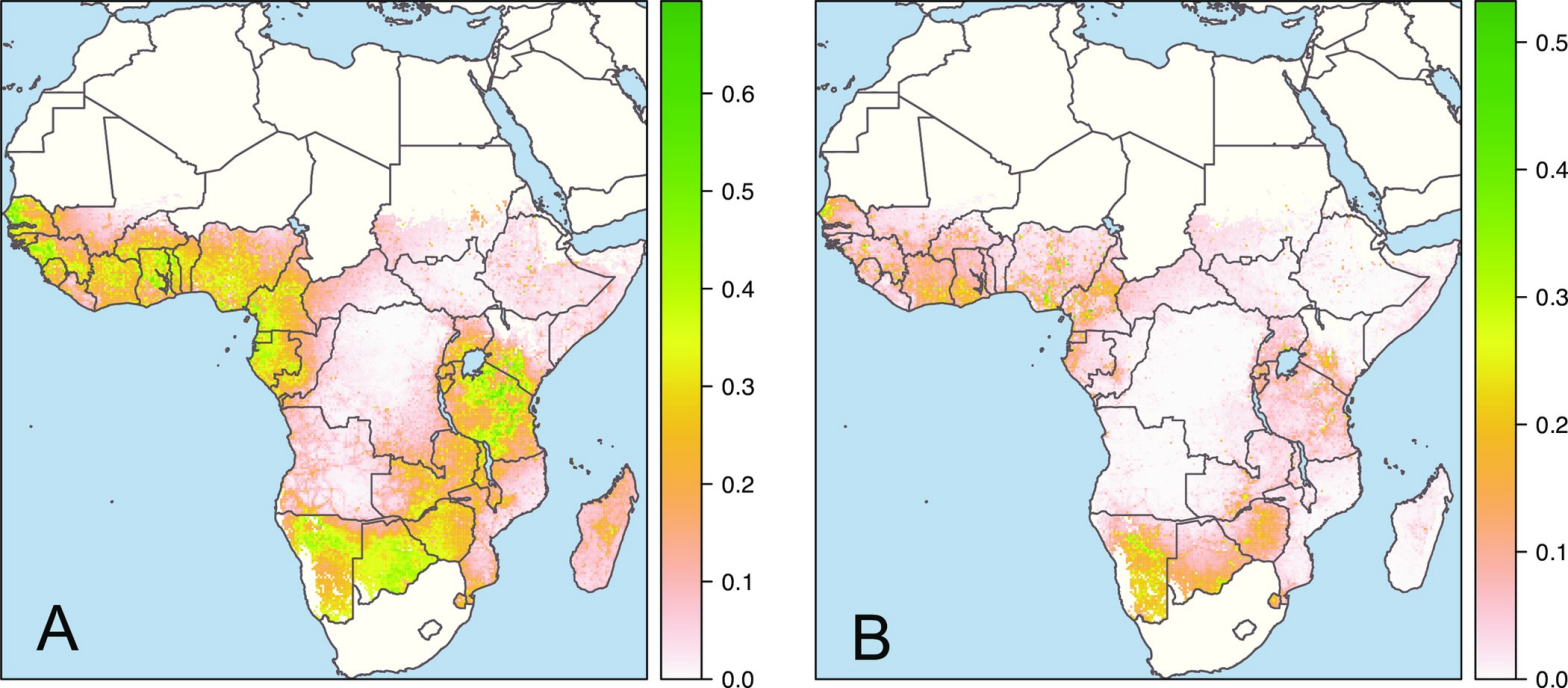
Changes in housing in sub-Saharan Africa between 2000 and 2015. The maps show the absolute difference in prevalence (scale 0 to 1) of housing built with finished materials (A) and improved housing (B) in 2000 and 2015. Houses built with finished materials were those with at least two of three of the wall, roof, and floor made from finished materials (e.g., parquet, vinyl, tiled, cement, or carpet floor), rather than natural or unfinished materials (e.g., earth, sand, dung, or palm floor). Improved houses were those with improved water and sanitation, sufficient living area, and finished building materials. Results are derived from a geospatial model fitted to 62 surveys representing 661,945 households (building materials) and 59 surveys representing 629,298 households (house type) [13]. Areas in green show the greatest changes in housing. *First published in* Nature *[13]. The base map was created by Samir Bhatt of the Malaria Atlas Project, Oxford*.

**Fig 2:**
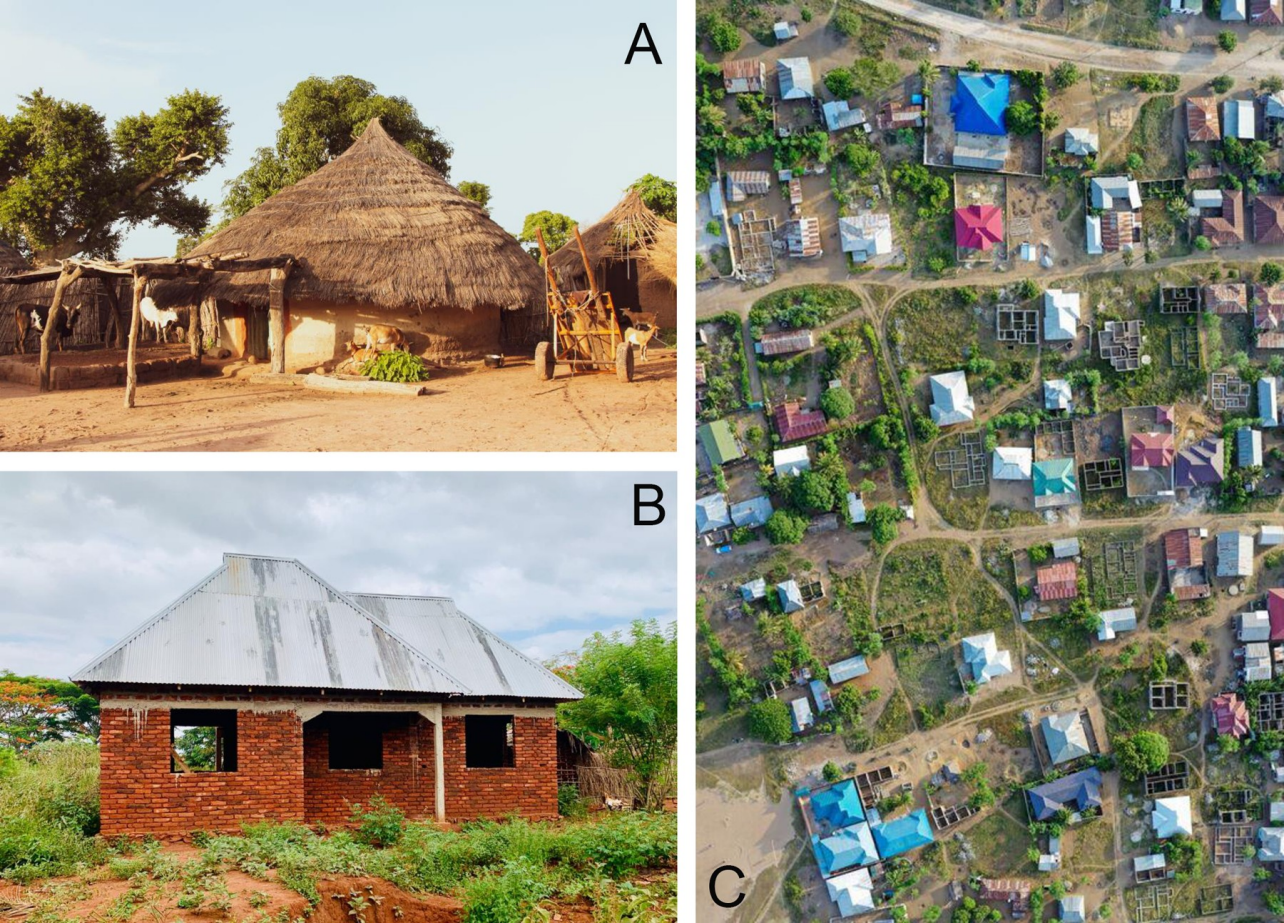
Housing development in sub-Saharan Africa. (A) Traditional, thatch-roof house in Upper River Region, The Gambia. (B) Modern building under construction in Tanga, Tanzania. (C) Incremental house construction in suburban Tanga, Tanzania. Houses range from approximately 20 years old with rusted roofs to new homes with coloured roofs. New foundations are also visible. *Photographs were taken by Jakob Knudsen*.

Housing in SSA is currently undergoing an unprecedented modernisation, presenting a major opportunity to improve the health of millions of people. Rapid population growth in SSA will necessitate homes for an additional 1.3 billion people by 2050 relative to 2015 [12]. In addition, the current stock of housing is changing fast alongside economic development, with the prevalence of housing with improved water and sanitation, sufficient living area, and durable construction doubling from 11% in 2000 to 23% in 2015 across SSA, excluding South Africa and desert areas (Figs 1 and 2) [13]. Leveraging these changes requires a detailed understanding of the relationship between house design and health and of the ways in which improvements in housing impact on individual causes of child morbidity and mortality. Studies have provided insight in specific settings [14, 15], but there remains an overall paucity of evidence, limiting our understanding of how ‘healthy’ housing can be designed and scaled-up, as well as its potential impact.

Here, we analyse the relationship between housing conditions and child health in SSA, providing standardised data across geographies and populations. We assess two aspects of housing aligning with a recent study that mapped changes in housing from 2000 to 2015 in SSA [13]: house construction materials (comparing houses built from finished materials [e.g., parquet, vinyl, tiled, cement, or carpet floor] with natural or unfinished materials [e.g., earth, sand, dung, or palm floor]) and overall house type (a UN definition that compares houses with improved water and sanitation, finished building materials, and sufficient living area with all other houses) (Fig 1). We use national survey data to test the hypothesis that improvements in these housing indicators are associated with reductions in five of the major causes of child mortality in SSA: (1) malaria, (2) diarrhoea, (3) ARI, (4) anaemia, and (5) malnutrition. To our knowledge, this is the only comprehensive study of the relationship between housing quality and multiple child health outcomes in SSA to date.

## Methods

### Data source

The prospective analysis plan is included as S1 Text, and there were no changes to the analysis. This study is reported according to the Strengthening the Reporting of Observational Studies in Epidemiology (STROBE) guidelines (S1 Checklist). We conducted a cross-sectional analysis of data from Demographic and Health Surveys (DHS), Malaria Indicator Surveys (MIS), and AIDS Indicator Surveys (AIS), which collect nationally representative health and sociodemographic data [16]. Informed consent to participate in DHS, MIS, and AIS interviews and biomarker tests is obtained orally. Individual survey protocols and questionnaires are reviewed by the ICF Institutional Review Board (IRB), which adheres to the United States Department of Health and Human Services regulations for the protection of human subjects (45 CFR 46), and typically also by an IRB in the host country [17]. DHS, MIS, and AIS have a two-stage sampling strategy, in which clusters are randomly selected from census files and households are randomly selected in each cluster. Eligible children are those who are in the specified age group (0–5 years) and are either usual members of the selected household or slept in the household the night before the interviewer’s visit. We analysed all DHS, MIS, and AIS conducted in SSA that measured malaria infection, diarrhoea, ARI, anthropometric data, and/or anaemia, as well as housing variables and all prespecified covariables.

### Health outcomes in children aged 0–5 years

Malaria infection was determined using a rapid diagnostic test (RDT) and/or microscopy using thick or thin blood smears. Diarrhoea was defined as any reported episode of diarrhoea (the passage of three or more loose or liquid stools per day) in the past 2 weeks. ARI was defined as a caregiver perceiving their child to have had in the past 2 weeks a cough; faster breathing than usual with short, quick breaths; or difficulty in breathing, excluding children who had only a blocked nose [18]. Stunting was defined as a height-for-age *z* score (HAZ) two or more standard deviations below the reference median, wasting a weight-for-height *z* score (WHZ) < −2, and underweight a weight-for-age *z* score (WAZ) < −2. Haemoglobin (Hb) concentrations were determined using the HemoCue system, and results were adjusted for altitude using a standard conversion [19]. Anaemia was classified as none (Hb ≥ 12.0 g/dL), mild (10.0–11.9 g/dL), moderate (7.0–9.9 g/dL), or severe (<7.0 g/dL). We analysed two anaemia outcomes: children with any anaemia and children with moderate or severe anaemia.

### Housing quality

Following a recent analysis [13], we categorised housing into two variables based on (1) house construction materials and (2) overall house type. All included surveys recorded the main materials used for the roof, walls, and/or floor and classified these as ‘natural’ (e.g., earth floor), ‘rudimentary’ (e.g., bamboo floor), or ‘finished’ (e.g., parquet floor) [16]. We classified houses as ‘built from finished materials’ if at least two of three variables of the wall, roof, and floor materials were finished and otherwise as ‘built from natural or unfinished materials’. We classified house type using an international definition [20] in which improved houses have all of the four following characteristics: (1) improved water supply (as defined by the World Health Organization Joint Monitoring Programme (WHO-JMP) [21]), (2) improved sanitation (as defined by WHO-JMP [21]), (3) three or fewer people per bedroom, and (4) house made of finished material(s). Houses lacking any of these characteristics were considered ‘unimproved’ [20] (S2 Text).

### Household wealth

DHS and MIS household wealth index scores are developed using principal component analysis (PCA) that typically includes variables describing durable asset ownership, access to utilities and infrastructure, and house construction materials [22]. In order to adjust for confounding by wealth in our analysis, we constructed a new wealth index for each survey that did not include variables related to house construction. We applied inclusion criteria [23] of (1) fewer than 10% missing values and (2) population frequency between 5% and 95% for the following assets, which are commonly used in DHS and MIS wealth indices: (a) car, (b) motorboat, (c) scooter, (d) cart, (e) bicycle, (f) television, (g) refrigerator, (h) radio, (i) watch, (j) mobile telephone, (k) landline telephone, and (l) electrification of the household. To condense these 12 assets into a single dimensional index, we tested isometric mapping, kernel principle component analysis, t-distributed stochastic neighbour embedding, and linear PCA. We found minimal differences between algorithms, so we used linear PCA [24].

### Association between housing quality and child health outcomes

For each survey, we modelled the association between house types and the odds of each health outcome, adjusting for prespecified variables (S3 Text). We used exact conditional logistic regression to enable associations to be estimated within geographical clusters, minimising confounding due to intercluster variation in disease risk and other factors. Because all children within geographical clusters are surveyed at a similar time point, conducting our analysis within clusters enabled comparisons to be made between children exposed to the same disease transmission seasons and prevention programmes. This approach was consistent with the underlying survey design. The mean cluster size was 25 children (range: 1–238 children). The analysis was restricted to children aged 0–5 years because of the availability of biomarker data for this age group only. All variables were included as categorical variables (including integer age), allowing nonlinearities to be modelled, except wealth score, which was included as a continuous variable. We explored nonlinear parameterisations of wealth using restricted cubic and B-splines, but these had no benefit over the linear model in terms of estimate concordance. We also explored sparsity promoting *l*_1_ penalised versions, but these had no benefit over unpenalised models. We preprocessed surveys to ensure that (1) missingness was not >10% for any variable, (2) data with no outcome variation in a given strata were removed, (3) constant covariates were removed, and (4) there were at least 100 observations after processing. Individual survey odds ratios (ORs) were combined to produce a summary OR using random-effects meta-analysis [25]. We opted for a two-stage approach to the analysis (whereby each survey was individually analysed and then individual survey ORs were pooled) over a one-stage approach [26] to maximise data use and ensure a consistent analysis because not all outcomes and covariables were collected by all surveys. Analyses were conducted in Stata16 (StataCorp, Texas) and R version 3.6.0 (R Core Team, Vienna).

## Results

### Study population

Characteristics of study participants and full results are presented in Table 1 and S4 and S5 Texts. Data were extracted for 121 surveys, of which 77 surveys were analysed, comprising 54 DHS, 21 MIS, and two AIS dating from 2001 to 2017 in 33 countries (Table A in S4 Text). The 44 surveys that did not measure any outcomes of interest and/or lacked key covariables were not analysed. The total study population comprised 824,694 children aged 0–5 years, resident in 468,471 households. The mean age of children was 2.5 years, and 49.7% were female.

**Table 1:** Characteristics of study participants.

Survey	Household-level characteristics						Child-level characteristics		
	*N*	Improved drinking water source (%)	Improved sanitation (%)	House built with finished materials (%)	Improved house (%)	Household head attended secondary education (%)	*N*	Mean age (years)	Male (%)
Angola 2011 MIS	8,391	48.1	44.9	45.6	15.0	-	9,681	2.4	49.9
Angola 2015 DHS	16,109	50.4	56.1	43.7	15.0	37.1	18,311	2.5	49.6
Benin 2001 DHS	5,769	53.3	17.0	54.3	-	17.0	6,250	2.5	50.1
Benin 2006 DHS	17,511	70.3	17.2	56.6	7.6	19.2	19,444	2.5	50.4
Benin 2012 DHS	17,422	77.2	29.1	60.8	14.3	20.4	17,489	2.6	51.0
Burkina Faso 2010 DHS	14,424	79.0	32.9	48.7	18.7	10.9	16,969	2.4	50.9
Burkina Faso 2014 MIS	6,448	76.8	42.6	45.1	17.1	-	8,419	2.5	50.9
Burundi 2010 DHS	8,596	76.1	44.3	42.8	14.5	11.7	9,025	2.4	50.3
Burundi 2012 MIS	4,866	80.5	77.7	42.9	23.7	11.2	4,985	2.4	49.7
Burundi 2016 DHS	15,977	83.0	54.8	54.7	25.1	13.1	15,544	2.5	50.3
Cameroon 2011 DHS	14,214	68.0	56.5	60.9	31.0	39.0	14,276	2.4	49.7
Comoros 2012 DHS	4,482	89.3	38.2	80.4	20.4	32.5	3,933	2.4	50.0
Congo 2005 DHS	5,879	71.5	20.5	70.5	12.0	63.0	5,753	2.4	50.3
Congo 2011 DHS	11,632	53.1	23.1	53.5	10.9	58.6	11,145	2.4	50.6
Cote d'Ivoire 2012 DHS	9,686	79.0	45.5	74.2	23.3	22.1	9,742	2.5	50.0
DRC 2013 DHS	18,171	40.3	37.6	19.8	5.6	52.8	22,059	2.4	49.6
Eswatini 2006 DHS	4,843	71.5	41.2	86.6	29.2	44.6	3,713	2.5	49.4
Ethiopia 2016 DHS	16,650	69.2	25.4	27.5	9.9	19.7	12,794	2.5	51.1
Gabon 2012 DHS	9,755	80.4	38.8	70.7	26.5	50.2	7,446	2.3	49.9
The Gambia 2013 DHS	6,217	90.4	58.2	79.1	33.0	25.4	10,701	2.4	50.9
Ghana 2008 DHS	11,778	77.6	65.4	79.8	30.2	55.8	7,411	2.5	50.8
Ghana 2014 DHS	11,835	65.9	68.0	89.3	25.8	57.6	7,341	2.4	51.9
Ghana 2016 MIS	5,841	61.1	66.3	86.2	22.2	-	4,159	2.5	50.8
Guinea 2012 DHS	7,109	73.4	45.0	58.7	24.8	19.3	8,531	2.5	51.4
Kenya 2008 DHS	9,057	64.3	50.0	49.3	22.5	34.4	7,231	2.4	51.4
Kenya 2014 DHS	36,430	64.5	47.9	49.0	19.9	33.5	26,253	2.5	50.6
Kenya 2015 MIS	6,481	63.3	54.5	55.2	22.9	-	4,724	2.6	50.3
Lesotho 2009 DHS	9,396	78.0	33.9	57.4	14.3	23.3	5,987	2.5	49.4
Lesotho 2014 DHS	9,402	83.3	67.2	62.1	31.7	28.3	5,181	2.6	49.8
Liberia 2011 MIS	4,162	70.4	28.1	46.9	9.4	-	4,340	2.5	50.2
Liberia 2013 DHS	9,333	64.9	33.6	37.9	10.2	40.4	9,724	2.5	51.2
Liberia 2016 MIS	4,218	67.5	36.9	49.8	10.0	-	3926	2.5	49.9
Madagascar 2008 DHS	17,857	43.8	7.4	29.8	3.7	29.1	15,763	2.5	50.6
Madagascar 2011 MIS	8,094	46.8	15.1	31.1	5.9	-	8,109	2.5	50.9
Madagascar 2013 MIS	8,574	45.3	16.4	29.0	6.1	-	7,306	2.5	51.0
Malawi 2010 DHS	24,825	79.8	11.3	30.5	5.1	19.9	24,280	2.5	49.4
Malawi 2012 MIS	3,404	82.6	25.1	41.2	12.2	-	2,813	2.4	47.5
Malawi 2014 MIS	3,405	85.6	19.8	49.4	12.6	-	2,621	2.4	50.1
Malawi 2015 DHS	26,361	87.0	83.3	46.6	29.6	27.6	21,414	2.6	49.9
Malawi 2017 MIS	3,729	88.8	27.0	59.3	17.9	-	2,950	2.5	49.2
Mali 2012 DHS	10,107	67.9	43.8	31.4	17.0	14.1	12,882	2.5	50.9
Mali 2015 MIS	4,240	70.5	44.3	39.8	18.6	-	9,539	2.5	50.4
Mozambique 2011 DHS	13,919	58.3	22.9	33.3	9.1	17.2	12,683	2.4	49.9
Mozambique 2015 AIS	7,169	67.6	17.0	42.7	11.0	21.9	6,483	2.4	49.3
Namibia 2006 DHS	9,200	88.1	44.0	60.4	30.8	44.4	6,774	2.4	49.8
Namibia 2013 DHS	9,849	87.4	47.9	71.2	35.0	53.9	6,953	2.5	49.5
Niger 2012 DHS	10,750	69.7	27.4	15.1	7.8	10.4	15,291	2.5	50.4
Nigeria 2008 DHS	34,070	52.6	49.0	61.0	18.3	38.1	31,634	2.4	50.9
Nigeria 2010 MIS	5,895	55.6	43.8	62.4	16.3	39.0	6,941	2.4	50.9
Nigeria 2013 DHS	38,522	58.3	52.6	67.8	21.8	42.7	35,364	2.4	50.7
Nigeria 2015 MIS	7,744	61.9	51.5	70.8	20.9	45.9	8,290	2.5	50.4
Rwanda 2010 DHS	12,540	74.2	75.0	49.6	25.1	11.7	10,697	2.6	50.8
Rwanda 2015 DHS	12,698	74.2	71.7	36.3	22.3	13.6	9,505	2.5	50.5
Rwanda 2017 MIS	5,041	76.9	82.8	42.5	28.5	-	3,548	2.4	52.0
Senegal 2008 MIS	10,651	65.4	46.6	52.2	21.2	-	23,105	2.5	51.1
Senegal 2010 DHS	7,904	69.5	44.3	59.3	23.0	11.5	15,752	2.5	51.4
Senegal 2012 DHS	4,177	66.9	51.3	63.4	24.3	12.8	8,746	2.5	50.0
Senegal 2014 DHS	4,233	69.5	49.1	64.1	24.5	11.5	8,432	2.4	50.2
Senegal 2015 DHS	4,511	65.5	49.1	69.4	25.9	12.0	8,553	2.4	49.9
Senegal 2016 DHS	4,440	72.0	52.5	68.6	26.1	12.5	8,380	2.5	51.2
Sierra Leone 2008 DHS	7,284	55.2	46.7	41.6	15.4	26.0	7,426	2.4	50.1
Sierra Leone 2013 DHS	12,629	58.8	50.0	50.2	15.6	24.6	14,958	2.6	49.5
Sierra Leone 2016 MIS	6,719	62.2	42.3	52.2	16.6	-	8,460	2.5	50.4
Tanzania 2004 DHS	9,735	52.6	6.8	34.8	-	10.9	10,142	2.4	50.2
Tanzania 2010 DHS	9,623	52.4	25.5	43.7	11.2	14.0	10,107	2.5	49.5
Tanzania 2012 AIS	10,040	60.5	37.1	46.7	17.4	13.0	10,921	2.4	50.2
Tanzania 2017 MIS	9,330	61.1	58.7	56.5	23.7	-	9,623	2.5	50.5
Togo 2013 DHS	9,549	63.2	38.3	78.8	18.9	36.8	8,583	2.5	50.4
Uganda 2006 DHS	8,870	69.3	27.1	27.7	8.1	22.7	10,064	2.5	49.1
Uganda 2009 MIS	4,421	72.7	35.4	34.3	12.3	-	4,940	2.5	50.1
Uganda 2014 MIS	5,345	77.1	31.2	38.5	12.6	-	6,108	2.5	49.0
Uganda 2016 DHS	19,588	77.0	34.4	40.7	14.9	30.8	19,453	2.6	50.4
Zambia 2007 DHS	7,164	42.5	33.0	41.3	12.3	37.9	7,404	2.3	49.3
Zambia 2013 DHS	15,920	62.1	41.5	51.2	18.0	44.5	16,657	2.5	50.6
Zimbabwe 2005 DHS	9,285	77.3	63.4	67.2	35.9	48.9	7,284	2.6	50.4
Zimbabwe 2010 DHS	9,756	78.5	63.1	71.2	35.7	53.5	7,187	2.4	50.2
Zimbabwe 2015 DHS	10,534	81.6	69.7	79.6	43.0	61.7	8,082	2.6	49.6

Houses were classified as improved if they had an improved water supply (as defined by WHO-JMP) [21]), improved sanitation (as defined by WHO-JMP [21]), three or fewer people per bedroom and were made of finished materials.

Abbreviations: AIS, AIDS Indicator Survey; DHS, Demographic and Health Survey; MIS, Malaria Indicator Survey; WHO-JMP, World Health Organization Joint Monitoring Programme.

### Housing and insecticide-treated bed nets

Houses built with finished materials ranged in prevalence from 15% (Niger 2012 DHS) to 89% (Ghana 2014 DHS) (Table A in S4 Text). Across all surveys, the prevalence of houses built with finished materials was higher in urban areas (84%) than in rural areas (35%) (*p* < 0.001). The prevalence of improved houses (with improved water and sanitation, sufficient living area, and finished materials) ranged from 4% (Madagascar 2008 DHS) to 43% (Zimbabwe 2015 DHS). Overall, the prevalence of improved houses was higher in urban areas (37%) than in rural areas (10%) (*p* < 0.001). Insecticide-treated bed net (ITN) use ranged from 1% (Eswatini 2006 DHS) to 75% (Burkina Faso 2014 MIS) (Table B in S4 Text).

### Malaria infection

**Fig 3:**
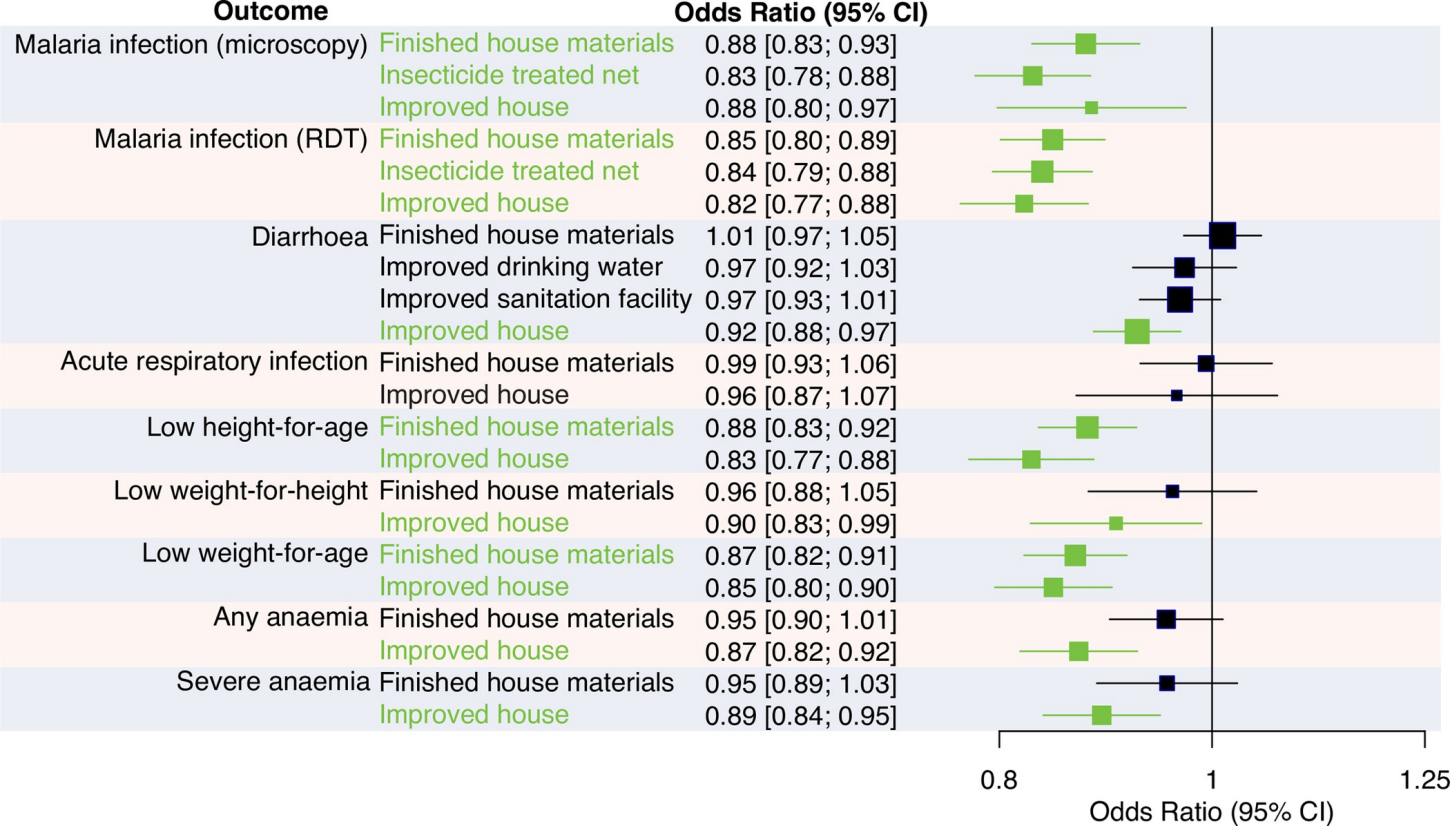
Association between housing conditions and health in children aged 0–5 years in sub-Saharan Africa. Data are from 824,694 children surveyed in 54 Demographic and Health, 21 Malaria Indicator, and two AIDS Indicator Surveys conducted between 2001 and 2017 in 33 countries. Houses built with finished materials were those with at least two of three of the wall, roof, and floor made from finished materials (e.g., parquet, vinyl, tiled, cement, or carpet floor), rather than natural or unfinished materials (e.g., earth, sand, dung, or palm floor). Improved houses were those with improved water and sanitation, sufficient living area, and finished building materials. For comparison, insecticide-treated bed net use is included for malaria infection, and type of water source and sanitation facility are included for diarrhoea. The pooled reduction in odds of each outcome is shown to the left of the vertical line representing the null value. Odds ratios are adjusted for a suite of covariates defined a priori, including age, gender, vaccination coverage, and household characteristics (S3 Text), as well as geographic cluster. Summary effects are from random-effects analysis. Error bars show 95% CIs, and green colouring shows *p* < 0.05. CI, confidence interval; RDT, rapid diagnostic test.

A total of 188,651 blood smears were taken in 38 of 77 surveys, of which 40,178 (21%) were malaria parasite positive (Table C in S4 Text). The prevalence of malaria infection measured by microscopy ranged from 0.5% (Senegal 2015 DHS) to 65% (Burkina Faso 2010 DHS). A total of 224,075 RDTs were done in 44 surveys, of which 60,547 (27%) were positive. Malaria infection prevalence measured by RDT ranged from 1% (Senegal 2015 DHS) to 76% (Burkina Faso 2010 DHS). Across all surveys, use of finished building materials was associated with a 12% reduction in the odds of malaria infection when measured by microscopy (adjusted OR [aOR] 0.88, 95% confidence interval (CI) 0.83–0.93, *p* < 0.001) and a 15% reduction when measured by RDT (aOR 0.85, 95% CI 0.80–0.89, *p* < 0.001) compared with houses with unfinished materials (Fig 3 and Table A in S5 Text). Similarly, improved housing was associated with a 12%–18% reduction in the odds of malaria infection compared with unimproved housing (microscopy: aOR 0.88, 95% CI 0.80–0.97, *p* = 0.01; RDT: aOR 0.82, 95% CI 0.77–0.88, *p* < 0.001) (Table C in S5 Text). In comparison, ITN use the night before the survey was associated with a 16%–17% reduction in the odds of malaria infection compared with no ITN use (microscopy: aOR 0.83, 95% CI 0.78–0.88, *p* < 0.001; RDT: aOR 0.84, 95% CI 0.79–0.88, *p* < 0.001) (Table E in S5 Text).

### Diarrhoea

Data on diarrhoea were available for 479,910 children in 55 surveys, 462,330 of whom from 53 surveys had sufficient covariable data to be included in the analysis. Of these children, 71,127 (15%) had a reported episode of diarrhoea in the previous 2 weeks. Diarrhoea prevalence ranged from 7% (Benin 2012 DHS) to 27% (Uganda 2006 DHS) (Table C in S4 Text). Overall, there was no association between diarrhoea and house construction materials (aOR 1.01, 95% CI 0.97–1.05, *p* = 0.60), type of drinking water source (aOR 0.97, 95% CI 0.92–1.03, *p* = 0.30), or sanitation facility (aOR 0.97, 95% CI 0.93–1.01, *p* = 0.12). Improved housing was, however, associated with an 8% reduction in the odds of diarrhoea (aOR 0.92, 95% CI 0.88–0.97, *p* = 0.001).

### Acute respiratory infection

Data on ARI were available for 155,609 children in 55 surveys, 121,179 of whom from 36 surveys were included in the analysis. Of these children, 37,121 (31%) had a reported cough accompanied by rapid breathing in the previous 2 weeks. ARI prevalence ranged from 5% (Angola 2015 DHS) to 80% (Burundi 2010 DHS) (Table C in S4 Text). There was no overall association between ARI and house construction materials (aOR 0.99, 95% CI 0.93–1.06, *p* = 0.86) or house type (aOR 0.96, 95% CI 0.87–1.07, *p* = 0.49).

### Stunting

Height and age were recorded for 343,773 children in 54 surveys, 326,047 of whom from 50 surveys were included in the analysis. Of these children, 98,763 (30%) were stunted. Stunting prevalence ranged from 15% (Ghana 2014 DHS) to 49% (Burundi 2010 DHS) (Table C in S4 Text). Overall, use of finished building materials was associated with a 12% reduction in the odds of stunting (aOR 0.88, 95% CI 0.83–0.92, *p* < 0.001) (Table B in S5 Text), and improved housing was associated with a 17% reduction in the odds of stunting (aOR 0.83, 95% CI 0.77–0.88, *p* < 0.001) (Table D in S5 Text).

### Wasting

Weight and height were recorded for 344,012 children in 53 surveys, 325,344 of whom from 49 surveys were included in the analysis. Of these children, 24,566 (8%) were wasted. Wasting prevalence ranged from 2% (Rwanda 2015 DHS) to 16% (Niger 2012 DHS) (Table C in S4 Text). Overall, use of house construction materials was not associated with wasting, but improved housing was associated with a 10% reduction in the odds of wasting (aOR 0.90, 95% CI 0.83–0.99, *p* = 0.03).

### Underweight

Weight and age were recorded for 338,283 children in 53 surveys, 320,557 of whom from 49 surveys were included in the analysis. Of these children, 70,810 (22%) were underweight. Underweight prevalence ranged from 7% (Eswatini 2006 DHS) to 41% (Niger 2012 DHS) (Table C in S4 Text). Overall, use of finished building materials was associated with a 13% reduction in the odds of underweight (aOR 0.87, 95% CI 0.82–0.91, *p* < 0.001), and improved housing was associated with a 15% reduction in the odds of underweight (aOR 0.85, 95% CI 0.80–0.90, *p* < 0.001).

### Anaemia

Hb concentration was measured in 306,417 children in 67 surveys, 182,281 of whom from 40 surveys were included in the analysis. Of these children, 116,628 (64%) had any degree of anaemia, and 70,447 (39%) had moderate or severe anaemia. The prevalence of any anaemia ranged from 35% (Kenya 2015 MIS) to 88% (Burkina Faso 2010 DHS), and the prevalence of moderate or severe anaemia ranged from 14% (Rwanda 2010 DHS) to 70% (Burkina Faso 2014 MIS) (Table C in S4 Text). Overall, there was no association between house construction materials and anaemia, but improved housing was associated with a 11%–13% reduction in the odds of anaemia (any degree of anaemia: aOR 0.87, 95% CI 0.82–0.92, *p* < 0.001; moderate to severe anaemia: aOR 0.89, 95% CI 0.84–0.95, *p* < 0.001).

## Discussion

We hypothesised that improved housing is associated with lower odds of malaria infection, diarrhoeal disease, ARI, growth failure, and anaemia in SSA. By analysing data from 77 national surveys, to our knowledge we have conducted the first comprehensive analysis of housing conditions relative to multiple child health outcomes across SSA. Use of finished house materials was associated with a 12%–15% reduction in the odds of malaria infection as well as stunting (12% reduction) and underweight (13% reduction) compared with unfinished materials, although there was no association with diarrhoea, ARI, wasting, or anaemia. Housing with improved water and sanitation, sufficient living area, and finished materials was associated with a 12%–18% reduction in the odds of malaria infection as well as diarrhoea (8% reduction), stunting (17% reduction), wasting (10% reduction), underweight (15% reduction), any anaemia (13% reduction), and moderate to severe anaemia (11% reduction) compared with unimproved housing. In comparison, ITN use was associated with a 16%–17% reduction in the odds of malaria infection compared with no ITNs. There was no association between drinking water source or sanitation facility alone and diarrhoea.

Of the five health outcomes evaluated, the quality of housing was most consistently associated with malaria infection and anaemia. There are three main explanations for these relationships. First, well-built housing with few entry points for malaria vectors can lower human exposure to infectious bites. Protective features may include screened doors and windows, the presence of a ceiling, and closed eaves [27]. We did not have data to analyse these features but, instead, evaluated finished house materials, which are often associated with modern housing styles that incorporate features such as closed eaves [14]. Second, house design may affect malaria transmission via indoor climate [28]. Metal-roofed houses are hotter in the daytime than traditional thatched homes, possibly lowering mosquito survival and inhibiting parasite development within the mosquito [29, 30], although the greater accumulation of CO_2_ in metal-roofed than thatch-roof homes may attract more vectors indoors [31]. Third, household crowding, incorporated into our overall measure of house type, can increase vector attraction [32]. The reduction in malaria prevalence associated with improved housing and use of an ITN was similar (16%–18% and 16%–17% reductions, respectively). Our study builds on a previous meta-analysis of 29 national surveys with similar findings: a 9%–14% reduction in the odds of malaria infection associated with finished house materials and a 15%–16% reduction associated with ITN use [23]. The findings confirm housing conditions as an important risk factor for malaria across SSA.

Beyond malaria, we conducted a comprehensive geographical analysis of housing conditions in relation to a range of key child health outcomes. We found that finished house materials were associated with child growth but not with the incidence of diarrhoea, ARIs, or anaemia. However, when finished house materials were combined with improved water source, improved sanitation facility, and sufficient living area, there was a clear association between improved housing and greater child growth, but less so for diarrhoea and anaemia. Diarrhoea prevention is based on reducing faecal–oral transmission of pathogens through better water, sanitation, and hygiene (WASH), including an improved water supply (e.g., a piped connection into the home), improved sanitation facility (e.g., flush toilets to a confined system), and handwashing with soap [33]. We found no association between diarrhoea and either water source or sanitation facility alone. It is possible that a very high standard of improvements is needed to reduce diarrhoea. Additionally, the use of self-reported diarrhoea is limited by the possible misclassification of cases, one consequence being that any association with more severe forms of infection may be masked.

Improved housing was strongly associated with reductions in stunting, wasting, and underweight. These indicators declined in nearly all African countries between 2000 and 2015, but the prevalence of stunting remains high in many settings [34]. Growth interventions typically tackle underlying causes such as poor nutrition and repeated infections and are therefore largely nutrition and WASH focused. Current evidence, however, suggests that nutrition interventions alone are unlikely to reduce childhood undernutrition globally [35], and there is mixed evidence for the efficacy of WASH interventions for child growth [35]. Recent studies from The Gambia conclude that a very high threshold of improvements in living conditions, disease reduction, diet, and healthcare must be exceeded to eliminate malnutrition [15, 34]. Our findings support this hypothesis, although it remains unclear why reductions in stunting in SSA would not have occurred more quickly given the rapid changes in housing since 2000. A lack of affordable housing has been linked to inadequate nutrition in other settings [7]. Our study suggests that the key housing features for nutrition may not be structural but are related to access to improved water and sanitation. Further research is needed to understand the relationship between housing conditions and child growth, as well as possible benefits of concurrent reductions in malaria and diarrhoea.

We did not observe a relationship between ARI and housing conditions, which are linked primarily via indoor air quality, ventilation, and density of residents. In particular, air pollution caused by indoor cooking with polluting stoves and solid fuels causes over half of pneumonia deaths in children aged 0–5 years globally [36], and ARI risk may also increase with poor ventilation and crowding (although the evidence is mixed [37]). We did not observe any association between housing conditions and ARI, which may reflect no underlying link with the components of housing incorporated into our definition. Alternatively, it may be difficult for young children to avoid air pollution, either indoors or outdoors, if they remain close to their caregiver as they cook. Ventilation may also be reduced in modern houses with closed eaves and more screening, which is potentially concerning. Self-reported ARI is also nonspecific, which may have reduced the strength of our analysis. Because ARI is the leading cause of mortality among children aged 1–5 years globally [38], a better understanding of the relationship between housing conditions and ARI is urgently needed.

A key consideration in our analysis is household wealth, which is associated closely with both improved housing conditions [13] and better health. We controlled for household wealth using an asset-based wealth index and education level of the household head, but fully separating housing conditions from wealth is impossible in observational studies, and residual confounding is possible. For example, wealthier households are more likely to live in improved houses and have better nutrition and higher rates of exclusive breastfeeding, leading to a reduction in anaemia and growth failure in children. Our metric of improved housing should therefore be interpreted as representing not only the physical characteristics of the home but also the wider social environment. From this perspective, our findings provide optimism for health as SSA continues to develop while emphasising the unacceptable burden of disease amongst the poorest households and the need for health interventions to acknowledge widely embedded social and environmental inequalities.

Today, housing in SSA is transforming, with the prevalence of housing built with finished materials increasing from 32% in 2000 to 51% in 2015, and the prevalence of improved housing (with improved water and sanitation, sufficient living area, and finished materials) doubling from 11% to 23% in the same time period [13]. These changes have arisen primarily from individual households investing incrementally in their homes. We show that the same housing metrics are associated with a reduction in multiple child health outcomes, with two implications. First, improved living conditions may have contributed to reductions in childhood disease since 2000, particularly for malaria, which declined rapidly over the Millennium Development Goal era. Second, Africa’s rapid population growth (from 1.2 billion in 2015 to an estimated 2.5 billion by 2050 [12]) coupled with the world’s fastest urbanisation rate [39] is creating unprecedented demand for new homes. Consequently, the opportunity to improve health through housing is huge. Yet this may be missed without a dedicated, intersectoral effort. Published in 2018, WHO’s first housing and health guidelines primarily focus on crowding, indoor temperature, injury reduction, and injury hazards. Research recommendations are made for diarrhoea and respiratory infections in the context of WASH, crowding, and indoor climate, but nutrition and vector-borne diseases other than dengue are not mentioned [11]. Urgent research is needed to determine how ongoing changes in African housing can improve child health, as well as advocacy to highlight this important opportunity.

This study had several limitations. First, residual confounding by wealth is probable, as previously discussed. Second, our findings do not provide evidence of causality. Indeed, reverse causation is possible if households with more disease are poorer and invest less in their homes [40]. Third, risk factors for children’s poor health outcomes are highly complex. Although our analysis controlled for an extensive suite of covariables, including access to healthcare, vaccination coverage, and household wealth, our primary focus on housing as a primary risk factor should not detract from the importance of a wide range of causal factors in children’s health. Fourth, the diagnosis of diarrhoea and ARI was based on recall during the past 2 weeks, leading to possible misclassification of illness (e.g., a child with only a mild cough may have been diagnosed with an ARI) and the masking of any association with more severe forms of infection. We found no association between water and sanitation and diarrhoea, nor between housing conditions and ARI, despite these being linked in other settings [37]. Fifth, our metric of overall house type, which incorporates house materials, water, sanitation, and living area, may be more robust than our metric of housing materials alone, which was solely reliant upon arbitrary classifications of unfinished and finished materials. Sixth, we generalised across surveys with a wide range of cultures but small differences in questionnaire design, so our metrics may not have been universally appropriate. Nonetheless, the use of national survey data avoids biases typically associated with pooling observational data, including publication bias, selection bias, measurement bias, and selective outcome reporting, and provides estimates generalisable across a range of countries in SSA. Despite these limitations, our study suggests a consistent link between housing and health across SSA while highlighting the need for intervention studies to further examine this relationship.

In conclusion, housing conditions are associated with four key child health outcomes across SSA: malaria infection, diarrhoea, growth failure, and anaemia. Interventions that improve multiple syndemic health outcomes are likely to reduce childhood mortality to a greater extent than would be expected from individual estimates of protective efficacy for each health outcome. This is because a child is more likely to die when experiencing repeat episodes of malaria and diarrhoea while malnourished and anaemic. Changes in housing driven by socioeconomic development should be considered a major opportunity to improve children’s health in SSA alongside wider development goals.

## Supporting information

S1 Text Prospective analysis plan. (PDF)

S2 Text Classification of house materials. (PDF)

S3 Text Surveys included and variables adjusted for in the analysis. (PDF)

S4 Text Characteristics of included surveys and prevalence of child health outcomes by survey. (PDF)

S5 Text Association between housing conditions and health outcomes in children aged 0–5 years in sub-Saharan Africa. (PDF)

S1 Checklist STROBE statement. STROBE, Strengthening the Reporting of Observational Studies in Epidemiology.
(PDF)

## Abbreviations

AIS: AIDS Indicator Survey
aOR: adjusted odds ratio
ARI: acute respiratory infection
CI: confidence interval
DHS: Demographic and Health Survey
HAZ: height-for-age *z* score
Hb: haemoglobin
IRB: Institutional Review Board
ITN: insecticide-treated bed net
MIS: Malaria Indicator Survey
OR: odds ratio
PCA: principal component analysis
RDT: rapid diagnostic test
SSA: sub-Saharan Africa
STROBE: Strengthening the Reporting of Observational Studies in Epidemiology
UN: United Nations
WASH: water, sanitation, and hygiene
WAZ: weight-for-age *z* score
WHO-JMP: World Health Organization Joint Monitoring Programme
WHZ: weight-for-height *z* score
