# Supplementary material for: Housing and child health in sub-Saharan Africa: A cross-sectional analysis
Source: PLoS Med. 2020 Mar 23;17(3):e1003055. doi: 10.1371/journal.pmed.1003055 (PMC7089421; doi:10.1371/journal.pmed.1003055)
Supplement: S1 Text — (PDF) [file pmed.1003055.s001.pdf]

**S1 Text. Prospective analysis plan**

***Objective:*** To conduct a cross-sectional analysis to measure the association between house type and the odds of malaria, diarrhoea, anaemia, acute respiratory infection (ARI) and growth failure in children aged 0-5 years in sub-Saharan Africa.

***Data source:*** We will extract data from the Demographic and Health Surveys (DHS), Malaria Indicator Surveys (MIS) and AIDS Indicator Surveys (AIS).[1] DHS, MIS and AIS typically collect health and sociodemographic data every five years and select households using a two-stage random cluster sampling strategy. We will include in each analysis all DHS, MIS and AIS conducted in SSA that collected data on malaria infection in children (measured by rapid diagnostic test (RDT) or microscopy), reported diarrhoeal disease, reported ARIs, anaemia (measured using the HemoCue system) and anthropometric data as well as house construction materials and all prespecified covariables.

*Health outcomes in children aged <5 years:* Malaria infection in children is determined in surveys using an RDT and/or microscopy using thick or thin blood smears. We will classify children with diarrhoea as those whose caregivers reported them having diarrhoea in the previous two weeks. We will classify children with ARIs as those whose caregivers reported them having a cough in the two weeks preceding the survey accompanied by short and rapid breathing. We will define stunting as a height-for-age *z* score (HAZ) that is two or more standard deviations below the reference median, wasting as a weight-for-height *z* score (WHZ) of $<-2$ and bing underweight as a weight-for-age *z* score (WAZ) of $<-2$. Haemoglobin levels are determined in surveys using the HemoCue system, adjusted for altitude. Anaemia is classified as none (≥12.0 g/dl), mild (10.0–11.9 g/dl), moderate (7.0–9.9 g/dl), and severe (< 7.0 g/dl). We will analyse two anaemia outcomes: children with any anaemia (mild, moderate, or severe) and children with moderate to severe anaemia.

*Housing quality:* As in a previous analysis[2] we will categorise house type into two variables: (1) house construction materials and (2) overall house type. DHS, MIS and AIS record the main materials used for the roof, walls and/or floor and categorize these as 'natural', 'rudimentary' or 'finished' (e.g. finished floor materials include parquet, vinyl, ceramic tiles, cement and carpet, while natural or rudimentary floor materials include earth, sand, dung, wood planks, palm and bamboo[3]). We will classify houses as 'built from finished materials' if at least two out of three of the wall, roof and floor materials are finished and as 'built from natural or unfinished materials' if this criterion is not met. We will categorise overall house type using an international definition[4] where unimproved houses are considered to have at least one of four characteristics: (1) unimproved water supply (as defined by the World Health Organization Joint Monitoring Programme (WHO-JMP)[5]); (2) unimproved sanitation (as defined by WHO-JMP[5]), (3) more than three people per bedroom and (4) house made of natural or unfinished material. Houses with none of these characteristics will be considered to be 'improved'. Following UN protocol, we will exclude a fifth characteristic of unimproved housing from our definition (insecurity of tenure) due to a lack of internationally comparable data.[4]

*Insecticide-treated bednet (ITN) use:* We will consider ITNs to be (i) long-lasting insecticide-treated nets that were ≤3-years-old at the time of survey or (ii) conventional ITNs that were ≤1-year-old or were retreated within the year before the survey.

*Household wealth:* DHS, MIS and AIS datasets divide households into wealth quintiles using wealth index scores that are calculated by entering a range of variables into a factor analysis. These variables vary between surveys but typically include durable asset ownership, access to utilities and infrastructure

(e.g. sanitation facilities) and housing characteristics. However, the inclusion of housing materials, crowding, drinking water source and sanitation facilities in the wealth index may underestimate the association between housing quality and health outcomes, when controlling for household wealth. For each country, we will therefore re-construct country wealth indices, excluding housing materials, crowding, drinking water source and sanitation facilities. We will exclude assets where <5% or >95% of households own the asset.[6]

***Association between housing quality and child health outcomes:*** For each DHS, MIS and AIS survey, we will model the association between house type and the odds of each outcome of interest in children aged 0-5 years using conditional logistic regression. We will adjust for the covariables prespecified in Table S1. As a comparison, we will also analyse the relationship between ITN use and malaria infection and between drinking water source, sanitation facility and diarrhoea. The analysis will be restricted to children aged 0-5 years because national surveys collect biomarker data within this age range only. We will use conditional logistic regression to enable associations to be estimated within geographical cluster so that the analysis eliminates confounding due to inter-cluster variation in disease risk and other factors. Survey odds ratios (ORs) will be combined to determine a summary OR across all surveys using a random effects meta-analysis. Summary ORs will be displayed in a forest plot.

## References


1. ICF International. Demographic and Health Surveys (DHS) Program. Calverton, Maryland: ICF International, 2019. www.icf.com/resources/projects/research-and-evaluation/demographic-and-health-surveys (accessed June 27 2019).
2. Tusting LS, Bisanzio D, Alabaster G, et al. Mapping changes in housing in sub-Saharan Africa from 2000 to 2015. *Nature* 2019; **568**: 391-4.
3. ICF International. Demographic and Health Surveys Methodology. Calverton, Maryland: ICF International, 2011.
4. UN. Indicators for Monitoring the Millennium Development Goals: 7.10 Proportion of urban population living in slums. 2012. http://mdgs.un.org/unsd/mi/wiki/7-10-Proportion-of-urban-population-living-in-slums.ashx (accessed April 7 2018).
5. WHO-UNICEF Joint Monitoring Programme. Drinking-water and sanitation categories for monitoring purposes. 2016. http://www.wssinfo.org/definitions-methods/watsan-categories/ (accessed 18 August 2016).
6. Tusting LS, Rek JC, Arinaitwe E, et al. Measuring socioeconomic inequalities in relation to malaria risk: a comparison of metrics in rural Uganda. *Am J Trop Med Hyg* 2016; **94**: 650-8.

**Table A**. Covariables to include in the analysis, by outcome.

| **Outcome** | **Covariables** | |
|---|---|---|
| | **House construction materials** | **House type** |
| Malaria infection by microscopy or RDT | Child's age<br>Child's sex<br>Education level of household head*<br>Household wealth<br>ITN use<br>IRS in the past 12 months* | Child's age<br>Child's sex<br>Education level of household head*<br>Household wealth<br>ITN use<br>IRS in the past 12 months* |
| Diarrhoea | Child's age<br>Child's sex<br>Birthweight<br>Education level of household head*<br>Household wealth<br>Child has a health immunisation card<br>Type of water source<br>Type of sanitation facility<br>Vitamin A in the past 6 months<br>DTP-3 vaccination<br>Measles-1 vaccination<br>Rotavirus-2 vaccination* | Child's age<br>Child's sex<br>Birthweight<br>Education level of household head*<br>Household wealth<br>Child has a health immunisation card<br>Vitamin A in the past 6 months<br>DTP-3 vaccination<br>Measles-1 vaccination<br>Rotavirus-2 vaccination* |
| Anaemia | Child's age<br>Child's sex<br>Birthweight<br>ITN use<br>Education level of household head*<br>Household wealth<br>Child has a health immunisation card<br>Type of water source<br>Type of sanitation facility<br>Vitamin A in the past 6 months<br>DTP-3 vaccination<br>Measles-1 vaccination<br>Rotavirus-2 vaccination*<br>Pneumococcal-3 vaccination* | Child's age<br>Child's sex<br>Birthweight<br>ITN use<br>Education level of household head*<br>Household wealth<br>Child has a health immunisation card<br>Vitamin A in the past 6 months<br>DTP-3 vaccination<br>Measles-1 vaccination<br>Rotavirus-2 vaccination*<br>Pneumococcal-3 vaccination* |
| Acute respiratory infection | Child's age<br>Child's sex<br>Birthweight<br>Education level of household head*<br>Household wealth<br>Child has a health immunisation card<br>Frequency any household member smokes inside the house*<br>Type of cooking fuel*<br>Overcrowding<br>Vitamin A in the past 6 months<br>DTP-3 vaccination<br>Measles-1 vaccination<br>Pneumococcal-3 vaccination* | Child's age<br>Child's sex<br>Birthweight<br>Education level of household head*<br>Household wealth<br>Child has a health immunisation card<br>Frequency any household member smokes inside the house*<br>Type of cooking fuel*<br>Vitamin A in the past 6 months<br>DTP-3 vaccination<br>Measles-1 vaccination<br>Pneumococcal-3 vaccination* |
| Stunting, wasting and underweight | Child's age<br>Child's sex | Child's age<br>Child's sex |

| **Outcome** | **Covariables** | |
|---|---|---|
| | **House construction materials** | **House type** |
| | Birthweight<br>Education level of household head*<br>Household wealth<br>ITN use<br>Type of water source<br>Type of sanitation facility<br>Child has a health immunisation card<br>DTP-3 vaccination<br>Measles-1 vaccination | Birthweight<br>Education level of household head*<br>Household wealth<br>ITN use<br>Child has a health immunisation card<br>DTP-3 vaccination<br>Measles-1 vaccination |
| Anaemia | Child's age<br>Child's sex<br>Birthweight<br>ITN use<br>Education level of household head*<br>Household wealth<br>Child has a health immunisation card<br>Type of water source<br>Type of sanitation facility<br>Vitamin A in the past 6 months<br>DTP-3 vaccination<br>Measles-1 vaccination<br>Rotavirus-2 vaccination*<br>Pneumococcal-3 vaccination* | Child's age<br>Child's sex<br>Birthweight<br>ITN use<br>Education level of household head*<br>Household wealth<br>Child has a health immunisation card<br>Vitamin A in the past 6 months<br>DTP-3 vaccination<br>Measles-1 vaccination<br>Rotavirus-2 vaccination*<br>Pneumococcal-3 vaccination* |

*Variables that are infrequently recorded will be considered optional in the analysis. All other variables are required. Surveys will be excluded where missing data for required variables is >10%.
