# Supplementary material for: Housing and child health in sub-Saharan Africa: A cross-sectional analysis
Source: PLoS Med. 2020 Mar 23;17(3):e1003055. doi: 10.1371/journal.pmed.1003055 (PMC7089421; doi:10.1371/journal.pmed.1003055)
Supplement: S2 Text — (PDF) [file pmed.1003055.s002.pdf]

S2 Text. Classification of housing

Housing was categorized into two variables, as in a recent analysis that mapped changes in housing in sub-Saharan Africa from 2000 to 2015[1]:

1) *House construction materials*, comparing houses built from finished materials (e.g. cement or carpet floor) *versus* natural or unfinished materials (e.g. earth floor).[2]
2) *House type*, a United Nations definition of housing that compares houses with an improved water supply, improved sanitation, sufficient living area and finished materials with all other houses.[3]

House construction materials

The United Nations estimation procedure uses the building materials for the roof, walls and/or floor to measure the durability of housing.[4] DHS, MIS and AIS surveys classify wall, roof and floor materials as 'natural', 'rudimentary' or 'finished'[2] (Table S2a). Here, houses were classified as 'built with finished materials' if at least two out of three of the wall, roof and floor materials were finished, and as 'built with natural or unfinished materials' if this criterion was not met.

House type

We used the same categorization of house type as the Millennium Development Goals and Sustainable Development Goals.[3,4] We considered unimproved housing to have at least one of four characteristics: (1) unimproved water supply (as defined by the World Health Organization and United Nations International Children's Fund Joint Monitoring Programme (WHO-JMP)[5]); (2) unimproved sanitation (as defined by WHO-JMP[5]), (3) more than three people per bedroom and (4) house made of natural or unfinished material. Details of these components are below. Following UN protocol, we excluded a fifth characteristic of unimproved housing from our definition (insecurity of tenure) due to a lack of internationally comparable data.[3,4]

Drinking-water source and sanitation facility

We used the standard drinking-water and sanitation categories developed by WHO-JMP (Table S2b).[5] An 'improved' drinking-water source is considered one that adequately protects the source from outside contamination, particularly faecal matter. An 'improved' sanitation facility is one that hygienically separates human excreta from human contact.

Living area

Aligning with United Nations (UN) criteria, a house was considered to provide a sufficient living area for household members if not more than three people share the same sleeping room.[4]

References

1. Tusting LS, Bisanzio D, Alabaster G, et al. Mapping changes in housing in sub-Saharan Africa from 2000 to 2015. *Nature* 2019; **568**: 391-4.
2. ICF International. Demographic and Health Surveys Methodology. Calverton, Maryland: ICF International, 2011.
3. UN-Habitat. Concepts and definitions: SDG Indicator 11.1.1: Proportion of urban population living in slums, informal settlements or inadequate housing. Nairobi, Kenya: UN-Habitat, 2018.
4. UN. Indicators for Monitoring the Millennium Development Goals: 7.10 Proportion of urban population living in slums. 2012. http://mdgs.un.org/unsd/mi/wiki/7-10-Proportion-of-urban-population-living-in-slums.ashx (accessed 7 April 2018).
5. World Health Organization and United Nations International Children's Fund Joint Monitoring Programme. Drinking-water and sanitation categories for monitoring purposes. 2016. http://www.wssinfo.org/definitions-methods/watsan-categories/ (accessed 18 August 2016).

Table A. Classification of wall, roof and floor materials

**Wall**		**Roof**		**Floor**	
Natural or rudimentary	*Finished*	*Natural or rudimentary*	*Finished*	*Natural or rudimentary*	*Finished*
Animal dung	Baked bricks	Animal skin	Asbestos	Adobe	Brick
Bamboo	Burnt bricks	Bamboo	Brick tiles	Bamboo	Brick tiles
Bark	Burnt bricks with cement	Bark	Cement	Broken bricks	Carpet
Burnt bricks with mud	Cement	Basket weave	Cement fiber	Clay	Cement
Cane	Cement blocks	Cardboard	Ceramic tiles	Clay with sand	Cement screed
Cardboard	Ceramic	Dung	Clay tiles	Compressed earth	Cement tiles
Carton	Concrete	Earth	Concrete	Dung	Ceramic tiles
Clay	Corrugated asbestos	Grass	Corrugate metal	Earth	Concrete
Clay blocks	Corrugated metal	Leaves	Finished wood planks	Leaves	Granite
Dirt	Covered adobe	Makuti	Mud tiles	Matting	Linoleum
Grass	Finished brick	Matting	Roofing shingles	Mud	Marble
Mud blocks	Finished stone with cement	Mud	Roofing tiles	Palm	Other finished
Mud bricks	Finished wood planks	Mulch	Sheet metal	Reed	Parquet
No walls	Metal	No roof	Shingles	Rudimentary wood	Polished wood
Palm	Metal sheets	Palm	Slate	Sand	Rug
Palm branches	Semi-durable	Plastic sheet	Stone slabs	Stones	Tiles
Plastic	Stone with cement	Reed	Tiles	Wood batons	Vinyl
Recovered wood	Stone with lime	Rudimentary wood	Zinc/cement fiber	Wood planks	
Rudimentary - plywood	Stone with whitewash	Rustic mat			
Sand bricks	Tiles	Sod			
Shells (casca)	Tin	Sticks/mud/dung			
Sod	Unburnt bricks with cement	Straw			
Stone with clay		Tarpaulin			
Stone with mud		Thatch			
Straw		Tin cans			
Sun-dried bricks					
Tarpaulin					
Thatched mat					
Unburnt bricks					
Uncovered adobe					
Wattle-and-daub					
Wood and grass					
Wood planks					
Wood timber					
Wood with mud					
Wooden board					
Wooden plank					

Table B. World Health Organization Joint Monitoring Programme classification of drinking-water source and sanitation facilities[5]

	Drinking-water source	Sanitation facility
Improved	Piped water into dwelling<br>Piped water to yard/plot<br>Public tap or standpipe<br>Tubewell or borehole<br>Protected dug well<br>Protected spring<br>Rainwater	Flush toilet<br>Piped sewer system<br>Septic tank<br>Flush/pour flush to pit latrine<br>Ventilated improved pit latrine (VIP)<br>Pit latrine with slab<br>Composting toilet<br>Special case
Unimproved	Unprotected spring<br>Unprotected dug well<br>Cart with small tank/drum<br>Tanker-truck<br>Surface water<br>Bottled water	Flush/pour flush to elsewhere<br>Pit latrine without slab<br>Bucket<br>Hanging toilet or hanging latrine<br>Shared sanitation<br>No facilities or bush or field
