# Supplementary material for: Housing and child health in sub-Saharan Africa: A cross-sectional analysis
Source: PLoS Med. 2020 Mar 23;17(3):e1003055. doi: 10.1371/journal.pmed.1003055 (PMC7089421; doi:10.1371/journal.pmed.1003055)
Supplement: S3 Text — (PDF) [file pmed.1003055.s003.pdf]

**S3 Text.** Surveys included and variables adjusted for in the analysis

**Table A.** Surveys included (n=77) and covariables adjusted for in the analysis of house construction materials.

| Survey | Malaria infection (microscopy) | Malaria infection (RDT) | Diarrhoea | Acute respiratory infection | Low height-for-age | Low weight-for-height | Low weight-for-age | Anaemia |
|---|---|---|---|---|---|---|---|---|
| Angola 2011 MIS | Child's age, sex, ITN use, household wealth | Child's age, sex, ITN use, household wealth | - | - | - | - | - | - |
| Angola 2015 DHS | - | Child's age, sex, ITN use, IRS in the past 12 months, household wealth, education level of the household head | - | - | Child's age, sex, birthweight, healthcard ownership, DPT-3 vaccination, measles-1 vaccination, ITN use, drinking water source, sanitation facility, household wealth, education level of the household head | Child's age, sex, birthweight, healthcard ownership, DPT-3 vaccination, measles-1 vaccination, ITN use, drinking water source, sanitation facility, household wealth, education level of the household head | Child's age, sex, birthweight, healthcard ownership, DPT-3 vaccination, measles-1 vaccination, ITN use, drinking water source, sanitation facility, household wealth, education level of the household head | - |
| Benin 2001 DHS | - | - | Child's age, sex, birthweight, healthcard ownership, receipt of vitamin A, DPT-3 vaccination, measles-1 vaccination, drinking water source, sanitation facility, household wealth, education level of the household head | - | - | - | - | - |

**Table A.** Surveys included (n=77) and covariables adjusted for in the analysis of house construction materials.

| Survey | Malaria infection (microscopy) | Malaria infection (RDT) | Diarrhoea | Acute respiratory infection | Low height-for-age | Low weight-for-height | Low weight-for-age | Anaemia |
|---|---|---|---|---|---|---|---|---|
| Benin 2006 DHS | - | - | Child's age, sex, birthweight, healthcard ownership, receipt of vitamin A, DPT-3 vaccination, measles-1 vaccination, drinking water source, sanitation facility, household wealth, education level of the household head | Child's age, sex, birthweight, healthcard ownership, receipt of vitamin A, DPT-3 vaccination, measles-1 vaccination, main household cooking fuel, household crowding, household wealth, education level of the household head | Child's age, sex, birthweight, healthcard ownership, DPT-3 vaccination, measles-1 vaccination, ITN use, drinking water source, sanitation facility, household wealth, education level of the household head | Child's age, sex, birthweight, healthcard ownership, DPT-3 vaccination, measles-1 vaccination, ITN use, drinking water source, sanitation facility, household wealth, education level of the household head | Child's age, sex, birthweight, healthcard ownership, DPT-3 vaccination, measles-1 vaccination, ITN use, drinking water source, sanitation facility, household wealth, education level of the household head | Child's age, sex, birthweight, ITN use, healthcard ownership, receipt of vitamin A, DPT-3 vaccination, measles-1 vaccination, drinking water source, sanitation facility, household wealth, education level of the household head |
| Benin 2012 DHS | Child's age, sex, ITN use, IRS in the past 12 months, household wealth, education level of the household head | Child's age, sex, ITN use, IRS in the past 12 months, household wealth, education level of the household head | Child's age, sex, birthweight, healthcard ownership, receipt of vitamin A, DPT-3 vaccination, measles-1 vaccination, drinking water source, sanitation facility, household wealth, education level of the household head | - | Child's age, sex, birthweight, healthcard ownership, DPT-3 vaccination, measles-1 vaccination, ITN use, drinking water source, sanitation facility, household wealth, education level of the household head | Child's age, sex, birthweight, healthcard ownership, DPT-3 vaccination, measles-1 vaccination, ITN use, drinking water source, sanitation facility, household wealth, education level of the household head | Child's age, sex, birthweight, healthcard ownership, DPT-3 vaccination, measles-1 vaccination, ITN use, drinking water source, sanitation facility, household wealth, education level of the household head | Child's age, sex, birthweight, ITN use, healthcard ownership, receipt of vitamin A, DPT-3 vaccination, measles-1 vaccination, drinking water source, sanitation facility, household wealth, education level of the household head |

**Table A.** Surveys included (n=77) and covariables adjusted for in the analysis of house construction materials.

| **Survey** | **Malaria infection (microscopy)** | **Malaria infection (RDT)** | **Diarrhoea** | **Acute respiratory infection** | **Low height-for-age** | **Low weight-for-height** | **Low weight-for-age** | **Anaemia** |
|---|---|---|---|---|---|---|---|---|
| Burkina Faso 2010 DHS | Child's age, sex, ITN use, IRS in the past 12 months, household wealth, education level of the household head | Child's age, sex, ITN use, IRS in the past 12 months, household wealth, education level of the household head | Child's age, sex, birthweight, healthcard ownership, receipt of vitamin A, DPT-3 vaccination, measles-1 vaccination, drinking water source, sanitation facility, household wealth, education level of the household head | - | Child's age, sex, birthweight, healthcard ownership, DPT-3 vaccination, measles-1 vaccination, ITN use, drinking water source, sanitation facility, household wealth, education level of the household head | Child's age, sex, birthweight, healthcard ownership, DPT-3 vaccination, measles-1 vaccination, ITN use, drinking water source, sanitation facility, household wealth, education level of the household head | Child's age, sex, birthweight, healthcard ownership, DPT-3 vaccination, measles-1 vaccination, ITN use, drinking water source, sanitation facility, household wealth, education level of the household head | Child's age, sex, birthweight, ITN use, healthcard ownership, receipt of vitamin A, DPT-3 vaccination, measles-1 vaccination, drinking water source, sanitation facility, household wealth, education level of the household head |
| Burkina Faso 2014 MIS | Child's age, sex, ITN use, IRS in the past 12 months, household wealth | Child's age, sex, ITN use, IRS in the past 12 months, household wealth | - | - | - | - | - | - |
| Burundi 2010 DHS | - | - | Child's age, sex, birthweight, healthcard ownership, receipt of vitamin A, DPT-3 vaccination, measles-1 vaccination, drinking water source, sanitation facility, household wealth, education level of the household head | Child's age, sex, birthweight, healthcard ownership, receipt of vitamin A, DPT-3 vaccination, measles-1 vaccination, smoking in house, main household cooking fuel, household crowding, household wealth, education level of the household head | Child's age, sex, birthweight, healthcard ownership, DPT-3 vaccination, measles-1 vaccination, ITN use, drinking water source, sanitation facility, household wealth, education level of the household head | Child's age, sex, birthweight, healthcard ownership, DPT-3 vaccination, measles-1 vaccination, ITN use, drinking water source, sanitation facility, household wealth, education level of the household head | Child's age, sex, birthweight, healthcard ownership, DPT-3 vaccination, measles-1 vaccination, ITN use, drinking water source, sanitation facility, household wealth, education level of the household head | Child's age, sex, birthweight, ITN use, healthcard ownership, receipt of vitamin A, DPT-3 vaccination, measles-1 vaccination, drinking water source, sanitation facility, household wealth, education level of the household head |

**Table A.** Surveys included (n=77) and covariables adjusted for in the analysis of house construction materials.

| Survey | Malaria infection (microscopy) | Malaria infection (RDT) | Diarrhoea | Acute respiratory infection | Low height-for-age | Low weight-for-height | Low weight-for-age | Anaemia |
|---|---|---|---|---|---|---|---|---|
| Burundi 2012 MIS | Child's age, sex, ITN use, IRS in the past 12 months, household wealth, education level of the household head | Child's age, sex, ITN use, IRS in the past 12 months, household wealth, education level of the household head | - | - | - | - | - | - |
| Burundi 2016 DHS | Child's age, sex, ITN use, IRS in the past 12 months, household wealth, education level of the household head | Child's age, sex, ITN use, IRS in the past 12 months, household wealth, education level of the household head | Child's age, sex, birthweight, healthcard ownership, receipt of vitamin A, DPT-3 vaccination, measles-1 vaccination, rotavirus-2 vaccination, drinking water source, sanitation facility, household wealth, education level of the household head | Child's age, sex, birthweight, healthcard ownership, receipt of vitamin A, DPT-3 vaccination, measles-1 vaccination, pneumococcal-3 vaccination, smoking in house, main household cooking fuel, household crowding, household wealth, education level of the household head | Child's age, sex, birthweight, healthcard ownership, DPT-3 vaccination, measles-1 vaccination, ITN use, drinking water source, sanitation facility, household wealth, education level of the household head | Child's age, sex, birthweight, healthcard ownership, DPT-3 vaccination, measles-1 vaccination, ITN use, drinking water source, sanitation facility, household wealth, education level of the household head | Child's age, sex, birthweight, healthcard ownership, DPT-3 vaccination, measles-1 vaccination, ITN use, drinking water source, sanitation facility, household wealth, education level of the household head | Child's age, sex, birthweight, ITN use, healthcard ownership, receipt of vitamin A, DPT-3 vaccination, measles-1 vaccination, rotavirus-2 vaccination, pneumococcal-3 vaccination, drinking water source, sanitation facility, household wealth, education level of the household head |

**Table A.** Surveys included (n=77) and covariables adjusted for in the analysis of house construction materials.

| Survey | Malaria infection (microscopy) | Malaria infection (RDT) | Diarrhoea | Acute respiratory infection | Low height-for-age | Low weight-for-height | Low weight-for-age | Anaemia |
|---|---|---|---|---|---|---|---|---|
| Cameroon 2011 DHS | - | Child's age, sex, ITN use, IRS in the past 12 months, household wealth, education level of the household head | Child's age, sex, birthweight, healthcard ownership, receipt of vitamin A, DPT-3 vaccination, measles-1 vaccination, drinking water source, sanitation facility, household wealth, education level of the household head | Child's age, sex, birthweight, healthcard ownership, receipt of vitamin A, DPT-3 vaccination, measles-1 vaccination, main household cooking fuel, household crowding, household wealth, education level of the household head | Child's age, sex, birthweight, healthcard ownership, DPT-3 vaccination, measles-1 vaccination, ITN use, drinking water source, sanitation facility, household wealth, education level of the household head | Child's age, sex, birthweight, healthcard ownership, DPT-3 vaccination, measles-1 vaccination, ITN use, drinking water source, sanitation facility, household wealth, education level of the household head | Child's age, sex, birthweight, healthcard ownership, DPT-3 vaccination, measles-1 vaccination, ITN use, drinking water source, sanitation facility, household wealth, education level of the household head | Child's age, sex, birthweight, ITN use, healthcard ownership, receipt of vitamin A, DPT-3 vaccination, measles-1 vaccination, drinking water source, sanitation facility, household wealth, education level of the household head |
| Comoros 2012 DHS | - | - | Child's age, sex, birthweight, healthcard ownership, receipt of vitamin A, DPT-3 vaccination, measles-1 vaccination, drinking water source, sanitation facility, household wealth, education level of the household head | Child's age, sex, birthweight, healthcard ownership, receipt of vitamin A, DPT-3 vaccination, measles-1 vaccination, smoking in house, main household cooking fuel, household crowding, household wealth, education level of the household head | Child's age, sex, birthweight, healthcard ownership, DPT-3 vaccination, measles-1 vaccination, ITN use, drinking water source, sanitation facility, household wealth, education level of the household head | Child's age, sex, birthweight, healthcard ownership, DPT-3 vaccination, measles-1 vaccination, ITN use, drinking water source, sanitation facility, household wealth, education level of the household head | Child's age, sex, birthweight, healthcard ownership, DPT-3 vaccination, measles-1 vaccination, ITN use, drinking water source, sanitation facility, household wealth, education level of the household head | - |

**Table A.** Surveys included (n=77) and covariables adjusted for in the analysis of house construction materials.

| Survey | Malaria infection (microscopy) | Malaria infection (RDT) | Diarrhoea | Acute respiratory infection | Low height-for-age | Low weight-for-height | Low weight-for-age | Anaemia |
|---|---|---|---|---|---|---|---|---|
| Congo 2005 DHS | - | - | Child's age, sex, birthweight, healthcard ownership, receipt of vitamin A, DPT-3 vaccination, measles-1 vaccination, drinking water source, sanitation facility, household wealth, education level of the household head | Child's age, sex, birthweight, healthcard ownership, receipt of vitamin A, DPT-3 vaccination, measles-1 vaccination, main household cooking fuel, household crowding, household wealth, education level of the household head | Child's age, sex, birthweight, healthcard ownership, DPT-3 vaccination, measles-1 vaccination, ITN use, drinking water source, sanitation facility, household wealth, education level of the household head | Child's age, sex, birthweight, healthcard ownership, DPT-3 vaccination, measles-1 vaccination, ITN use, drinking water source, sanitation facility, household wealth, education level of the household head | Child's age, sex, birthweight, healthcard ownership, DPT-3 vaccination, measles-1 vaccination, ITN use, drinking water source, sanitation facility, household wealth, education level of the household head | Child's age, sex, birthweight, ITN use, healthcard ownership, receipt of vitamin A, DPT-3 vaccination, measles-1 vaccination, drinking water source, sanitation facility, household wealth, education level of the household head |
| Congo 2011 DHS | - | - | Child's age, sex, birthweight, healthcard ownership, receipt of vitamin A, DPT-3 vaccination, measles-1 vaccination, drinking water source, sanitation facility, household wealth, education level of the household head | Child's age, sex, birthweight, healthcard ownership, receipt of vitamin A, DPT-3 vaccination, measles-1 vaccination, smoking in house, main household cooking fuel, household crowding, household wealth, education level of the household head | Child's age, sex, birthweight, healthcard ownership, DPT-3 vaccination, measles-1 vaccination, ITN use, drinking water source, sanitation facility, household wealth, education level of the household head | Child's age, sex, birthweight, healthcard ownership, DPT-3 vaccination, measles-1 vaccination, ITN use, drinking water source, sanitation facility, household wealth, education level of the household head | Child's age, sex, birthweight, healthcard ownership, DPT-3 vaccination, measles-1 vaccination, ITN use, drinking water source, sanitation facility, household wealth, education level of the household head | Child's age, sex, birthweight, ITN use, healthcard ownership, receipt of vitamin A, DPT-3 vaccination, measles-1 vaccination, drinking water source, sanitation facility, household wealth, education level of the household head |

**Table A.** Surveys included (n=77) and covariables adjusted for in the analysis of house construction materials.

| Survey | Malaria infection (microscopy) | Malaria infection (RDT) | Diarrhoea | Acute respiratory infection | Low height-for-age | Low weight-for-height | Low weight-for-age | Anaemia |
|---|---|---|---|---|---|---|---|---|
| Cote d'Ivoire 2012 DHS | Child's age, sex, ITN use, IRS in the past 12 months, household wealth, education level of the household head | Child's age, sex, ITN use, IRS in the past 12 months, household wealth, education level of the household head | Child's age, sex, birthweight, healthcard ownership, receipt of vitamin A, DPT-3 vaccination, measles-1 vaccination, drinking water source, sanitation facility, household wealth, education level of the household head | Child's age, sex, birthweight, healthcard ownership, receipt of vitamin A, DPT-3 vaccination, measles-1 vaccination, smoking in house, main household cooking fuel, household crowding, household wealth, education level of the household head | Child's age, sex, birthweight, healthcard ownership, DPT-3 vaccination, measles-1 vaccination, ITN use, drinking water source, sanitation facility, household wealth, education level of the household head | Child's age, sex, birthweight, healthcard ownership, DPT-3 vaccination, measles-1 vaccination, ITN use, drinking water source, sanitation facility, household wealth, education level of the household head | Child's age, sex, birthweight, healthcard ownership, DPT-3 vaccination, measles-1 vaccination, ITN use, drinking water source, sanitation facility, household wealth, education level of the household head | Child's age, sex, birthweight, ITN use, healthcard ownership, receipt of vitamin A, DPT-3 vaccination, measles-1 vaccination, drinking water source, sanitation facility, household wealth, education level of the household head |
| DRC 2013 DHS | Child's age, sex, ITN use, household wealth, education level of the household head | Child's age, sex, ITN use, household wealth, education level of the household head | Child's age, sex, birthweight, healthcard ownership, receipt of vitamin A, DPT-3 vaccination, measles-1 vaccination, drinking water source, sanitation facility, household wealth, education level of the household head | Child's age, sex, birthweight, healthcard ownership, receipt of vitamin A, DPT-3 vaccination, measles-1 vaccination, smoking in house, main household cooking fuel, household crowding, household wealth, education level of the household head | Child's age, sex, birthweight, healthcard ownership, DPT-3 vaccination, measles-1 vaccination, ITN use, drinking water source, sanitation facility, household wealth, education level of the household head | Child's age, sex, birthweight, healthcard ownership, DPT-3 vaccination, measles-1 vaccination, ITN use, drinking water source, sanitation facility, household wealth, education level of the household head | Child's age, sex, birthweight, healthcard ownership, DPT-3 vaccination, measles-1 vaccination, ITN use, drinking water source, sanitation facility, household wealth, education level of the household head | Child's age, sex, birthweight, ITN use, healthcard ownership, receipt of vitamin A, DPT-3 vaccination, measles-1 vaccination, drinking water source, sanitation facility, household wealth, education level of the household head |

**Table A.** Surveys included (n=77) and covariables adjusted for in the analysis of house construction materials.

| Survey | Malaria infection (microscopy) | Malaria infection (RDT) | Diarrhoea | Acute respiratory infection | Low height-for-age | Low weight-for-height | Low weight-for-age | Anaemia |
|---|---|---|---|---|---|---|---|---|
| Eswatini 2006 DHS | - | - | Child's age, sex, birthweight, healthcard ownership, receipt of vitamin A, DPT-3 vaccination, measles-1 vaccination, drinking water source, sanitation facility, household wealth, education level of the household head | Child's age, sex, birthweight, healthcard ownership, receipt of vitamin A, DPT-3 vaccination, measles-1 vaccination, main household cooking fuel, household crowding, household wealth, education level of the household head | Child's age, sex, birthweight, healthcard ownership, DPT-3 vaccination, measles-1 vaccination, ITN use, drinking water source, sanitation facility, household wealth, education level of the household head | Child's age, sex, birthweight, healthcard ownership, DPT-3 vaccination, measles-1 vaccination, ITN use, drinking water source, sanitation facility, household wealth, education level of the household head | Child's age, sex, birthweight, healthcard ownership, DPT-3 vaccination, measles-1 vaccination, ITN use, drinking water source, sanitation facility, household wealth, education level of the household head | Child's age, sex, birthweight, ITN use, healthcard ownership, receipt of vitamin A, DPT-3 vaccination, measles-1 vaccination, drinking water source, sanitation facility, household wealth, education level of the household head |
| Ethiopia 2016 DHS | - | - | Child's age, sex, birthweight, healthcard ownership, receipt of vitamin A, DPT-3 vaccination, measles-1 vaccination, rotavirus-2 vaccination, drinking water source, sanitation facility, household wealth, education level of the household head | Child's age, sex, birthweight, healthcard ownership, receipt of vitamin A, DPT-3 vaccination, measles-1 vaccination, pneumococcal-3 vaccination, smoking in house, main household cooking fuel, household crowding, household wealth, education level of the household head | - | - | - | - |

**Table A.** Surveys included (n=77) and covariables adjusted for in the analysis of house construction materials.

| Survey | Malaria infection (microscopy) | Malaria infection (RDT) | Diarrhoea | Acute respiratory infection | Low height-for-age | Low weight-for-height | Low weight-for-age | Anaemia |
|---|---|---|---|---|---|---|---|---|
| Gabon 2012 DHS | - | - | Child's age, sex, birthweight, healthcard ownership, receipt of vitamin A, DPT-3 vaccination, measles-1 vaccination, drinking water source, sanitation facility, household wealth, education level of the household head | Child's age, sex, birthweight, healthcard ownership, receipt of vitamin A, DPT-3 vaccination, measles-1 vaccination, smoking in house, main household cooking fuel, household crowding, household wealth, education level of the household head | Child's age, sex, birthweight, healthcard ownership, DPT-3 vaccination, measles-1 vaccination, ITN use, drinking water source, sanitation facility, household wealth, education level of the household head | Child's age, sex, birthweight, healthcard ownership, DPT-3 vaccination, measles-1 vaccination, ITN use, drinking water source, sanitation facility, household wealth, education level of the household head | Child's age, sex, birthweight, healthcard ownership, DPT-3 vaccination, measles-1 vaccination, ITN use, drinking water source, sanitation facility, household wealth, education level of the household head | Child's age, sex, birthweight, ITN use, healthcard ownership, receipt of vitamin A, DPT-3 vaccination, measles-1 vaccination, drinking water source, sanitation facility, household wealth, education level of the household head |
| Gambia 2013 DHS | Child's age, sex, ITN use, IRS in the past 12 months, household wealth, education level of the household head | Child's age, sex, ITN use, IRS in the past 12 months, household wealth, education level of the household head | Child's age, sex, birthweight, healthcard ownership, receipt of vitamin A, DPT-3 vaccination, measles-1 vaccination, drinking water source, sanitation facility, household wealth, education level of the household head | - | Child's age, sex, birthweight, healthcard ownership, DPT-3 vaccination, measles-1 vaccination, ITN use, drinking water source, sanitation facility, household wealth, education level of the household head | Child's age, sex, birthweight, healthcard ownership, DPT-3 vaccination, measles-1 vaccination, ITN use, drinking water source, sanitation facility, household wealth, education level of the household head | Child's age, sex, birthweight, healthcard ownership, DPT-3 vaccination, measles-1 vaccination, ITN use, drinking water source, sanitation facility, household wealth, education level of the household head | Child's age, sex, birthweight, ITN use, healthcard ownership, receipt of vitamin A, DPT-3 vaccination, measles-1 vaccination, drinking water source, sanitation facility, household wealth, education level of the household head |

**Table A.** Surveys included (n=77) and covariables adjusted for in the analysis of house construction materials.

| Survey | Malaria infection (microscopy) | Malaria infection (RDT) | Diarrhoea | Acute respiratory infection | Low height-for-age | Low weight-for-height | Low weight-for-age | Anaemia |
|---|---|---|---|---|---|---|---|---|
| Ghana 2008 DHS | - | - | Child's age, sex, birthweight, healthcard ownership, receipt of vitamin A, DPT-3 vaccination, measles-1 vaccination, drinking water source, sanitation facility, household wealth, education level of the household head | - | Child's age, sex, birthweight, healthcard ownership, DPT-3 vaccination, measles-1 vaccination, ITN use, drinking water source, sanitation facility, household wealth, education level of the household head | Child's age, sex, birthweight, healthcard ownership, DPT-3 vaccination, measles-1 vaccination, ITN use, drinking water source, sanitation facility, household wealth, education level of the household head | Child's age, sex, birthweight, healthcard ownership, DPT-3 vaccination, measles-1 vaccination, ITN use, drinking water source, sanitation facility, household wealth, education level of the household head | Child's age, sex, birthweight, ITN use, healthcard ownership, receipt of vitamin A, DPT-3 vaccination, measles-1 vaccination, drinking water source, sanitation facility, household wealth, education level of the household head |
| Ghana 2014 DHS | Child's age, sex, ITN use, IRS in the past 12 months, household wealth, education level of the household head | Child's age, sex, ITN use, IRS in the past 12 months, household wealth, education level of the household head | Child's age, sex, birthweight, healthcard ownership, receipt of vitamin A, DPT-3 vaccination, measles-1 vaccination, drinking water source, sanitation facility, household wealth, education level of the household head | Child's age, sex, birthweight, healthcard ownership, receipt of vitamin A, DPT-3 vaccination, measles-1 vaccination, smoking in house, main household cooking fuel, household crowding, household wealth, education level of the household head | Child's age, sex, birthweight, healthcard ownership, DPT-3 vaccination, measles-1 vaccination, ITN use, drinking water source, sanitation facility, household wealth, education level of the household head | Child's age, sex, birthweight, healthcard ownership, DPT-3 vaccination, measles-1 vaccination, ITN use, drinking water source, sanitation facility, household wealth, education level of the household head | Child's age, sex, birthweight, healthcard ownership, DPT-3 vaccination, measles-1 vaccination, ITN use, drinking water source, sanitation facility, household wealth, education level of the household head | Child's age, sex, birthweight, ITN use, healthcard ownership, receipt of vitamin A, DPT-3 vaccination, measles-1 vaccination, drinking water source, sanitation facility, household wealth, education level of the household head |
| Ghana 2016 MIS | Child's age, sex, ITN use, IRS in the past 12 months, household wealth | Child's age, sex, ITN use, IRS in the past 12 months, household wealth | - | - | - | - | - | - |

**Table A.** Surveys included (n=77) and covariables adjusted for in the analysis of house construction materials.

| Survey | Malaria infection (microscopy) | Malaria infection (RDT) | Diarrhoea | Acute respiratory infection | Low height-for-age | Low weight-for-height | Low weight-for-age | Anaemia |
|---|---|---|---|---|---|---|---|---|
| Guinea 2012 DHS | Child's age, sex, ITN use, IRS in the past 12 months, household wealth, education level of the household head | Child's age, sex, ITN use, IRS in the past 12 months, household wealth, education level of the household head | Child's age, sex, birthweight, healthcard ownership, receipt of vitamin A, DPT-3 vaccination, measles-1 vaccination, drinking water source, sanitation facility, household wealth, education level of the household head | Child's age, sex, birthweight, healthcard ownership, receipt of vitamin A, DPT-3 vaccination, measles-1 vaccination, smoking in house, main household cooking fuel, household crowding, household wealth, education level of the household head | Child's age, sex, birthweight, healthcard ownership, DPT-3 vaccination, measles-1 vaccination, ITN use, drinking water source, sanitation facility, household wealth, education level of the household head | Child's age, sex, birthweight, healthcard ownership, DPT-3 vaccination, measles-1 vaccination, ITN use, drinking water source, sanitation facility, household wealth, education level of the household head | Child's age, sex, birthweight, healthcard ownership, DPT-3 vaccination, measles-1 vaccination, ITN use, drinking water source, sanitation facility, household wealth, education level of the household head | Child's age, sex, birthweight, ITN use, healthcard ownership, receipt of vitamin A, DPT-3 vaccination, measles-1 vaccination, drinking water source, sanitation facility, household wealth, education level of the household head |
| Kenya 2008 DHS | - | - | Child's age, sex, birthweight, healthcard ownership, receipt of vitamin A, DPT-3 vaccination, measles-1 vaccination, drinking water source, sanitation facility, household wealth, education level of the household head | Child's age, sex, birthweight, healthcard ownership, receipt of vitamin A, DPT-3 vaccination, measles-1 vaccination, main household cooking fuel, household crowding, household wealth, education level of the household head | Child's age, sex, birthweight, healthcard ownership, DPT-3 vaccination, measles-1 vaccination, ITN use, drinking water source, sanitation facility, household wealth, education level of the household head | Child's age, sex, birthweight, healthcard ownership, DPT-3 vaccination, measles-1 vaccination, ITN use, drinking water source, sanitation facility, household wealth, education level of the household head | Child's age, sex, birthweight, healthcard ownership, DPT-3 vaccination, measles-1 vaccination, ITN use, drinking water source, sanitation facility, household wealth, education level of the household head | - |

**Table A.** Surveys included (n=77) and covariables adjusted for in the analysis of house construction materials.

| Survey | Malaria infection (microscopy) | Malaria infection (RDT) | Diarrhoea | Acute respiratory infection | Low height-for-age | Low weight-for-height | Low weight-for-age | Anaemia |
|---|---|---|---|---|---|---|---|---|
| Kenya 2014 DHS | - | - | Child's age, sex, birthweight, healthcard ownership, receipt of vitamin A, DPT-3 vaccination, measles-1 vaccination, drinking water source, sanitation facility, household wealth, education level of the household head | Child's age, sex, birthweight, healthcard ownership, receipt of vitamin A, DPT-3 vaccination, measles-1 vaccination, smoking in house, main household cooking fuel, household crowding, household wealth, education level of the household head | Child's age, sex, birthweight, healthcard ownership, DPT-3 vaccination, measles-1 vaccination, ITN use, drinking water source, sanitation facility, household wealth, education level of the household head | Child's age, sex, birthweight, healthcard ownership, DPT-3 vaccination, measles-1 vaccination, ITN use, drinking water source, sanitation facility, household wealth, education level of the household head | Child's age, sex, birthweight, healthcard ownership, DPT-3 vaccination, measles-1 vaccination, ITN use, drinking water source, sanitation facility, household wealth, education level of the household head | - |
| Kenya 2015 MIS | Child's age, sex, ITN use, household wealth | Child's age, sex, ITN use, household wealth | - | - | - | - | - | - |
| Lesotho 2009 DHS | - | - | Child's age, sex, birthweight, healthcard ownership, receipt of vitamin A, DPT-3 vaccination, measles-1 vaccination, drinking water source, sanitation facility, household wealth, education level of the household head | - | - | - | - | - |

**Table A.** Surveys included (n=77) and covariables adjusted for in the analysis of house construction materials.

| Survey | Malaria infection (microscopy) | Malaria infection (RDT) | Diarrhoea | Acute respiratory infection | Low height-for-age | Low weight-for-height | Low weight-for-age | Anaemia |
|---|---|---|---|---|---|---|---|---|
| Lesotho 2014 DHS | - | - | Child's age, sex, birthweight, healthcard ownership, receipt of vitamin A, DPT-3 vaccination, measles-1 vaccination, drinking water source, sanitation facility, household wealth, education level of the household head | Child's age, sex, birthweight, healthcard ownership, receipt of vitamin A, DPT-3 vaccination, measles-1 vaccination, smoking in house, main household cooking fuel, household crowding, household wealth, education level of the household head | - | - | - | - |
| Liberia 2011 MIS | Child's age, sex, ITN use, IRS in the past 12 months, household wealth | Child's age, sex, ITN use, IRS in the past 12 months, household wealth | - | - | - | - | - | - |
| Liberia 2013 DHS | - | - | Child's age, sex, birthweight, healthcard ownership, receipt of vitamin A, DPT-3 vaccination, measles-1 vaccination, drinking water source, sanitation facility, household wealth, education level of the household head | Child's age, sex, birthweight, healthcard ownership, receipt of vitamin A, DPT-3 vaccination, measles-1 vaccination, smoking in house, main household cooking fuel, household crowding, household wealth, education level of the household head | Child's age, sex, birthweight, healthcard ownership, DPT-3 vaccination, measles-1 vaccination, ITN use, drinking water source, sanitation facility, household wealth, education level of the household head | Child's age, sex, birthweight, healthcard ownership, DPT-3 vaccination, measles-1 vaccination, ITN use, drinking water source, sanitation facility, household wealth, education level of the household head | Child's age, sex, birthweight, healthcard ownership, DPT-3 vaccination, measles-1 vaccination, ITN use, drinking water source, sanitation facility, household wealth, education level of the household head | - |

**Table A.** Surveys included (n=77) and covariables adjusted for in the analysis of house construction materials.

| Survey | Malaria infection (microscopy) | Malaria infection (RDT) | Diarrhoea | Acute respiratory infection | Low height-for-age | Low weight-for-height | Low weight-for-age | Anaemia |
|---|---|---|---|---|---|---|---|---|
| Liberia 2016 MIS | - | Child's age, sex, ITN use, IRS in the past 12 months, household wealth | - | - | - | - | - | - |
| Madagascar 2008 DHS | - | - | Child's age, sex, birthweight, healthcard ownership, receipt of vitamin A, DPT-3 vaccination, measles-1 vaccination, drinking water source, sanitation facility, household wealth, education level of the household head | - | Child's age, sex, birthweight, healthcard ownership, DPT-3 vaccination, measles-1 vaccination, ITN use, drinking water source, sanitation facility, household wealth, education level of the household head | - | - | Child's age, sex, birthweight, ITN use, healthcard ownership, receipt of vitamin A, DPT-3 vaccination, measles-1 vaccination, drinking water source, sanitation facility, household wealth, education level of the household head |
| Madagascar 2011 MIS | Child's age, sex, ITN use, IRS in the past 12 months, household wealth | Child's age, sex, ITN use, IRS in the past 12 months, household wealth | - | - | - | - | - | - |
| Madagascar 2013 MIS | Child's age, sex, ITN use, IRS in the past 12 months, household wealth | Child's age, sex, ITN use, IRS in the past 12 months, household wealth | - | - | - | - | - | - |

**Table A.** Surveys included (n=77) and covariables adjusted for in the analysis of house construction materials.

| Survey | Malaria infection (microscopy) | Malaria infection (RDT) | Diarrhoea | Acute respiratory infection | Low height-for-age | Low weight-for-height | Low weight-for-age | Anaemia |
|---|---|---|---|---|---|---|---|---|
| Malawi 2010 DHS | - | - | Child's age, sex, birthweight, healthcard ownership, receipt of vitamin A, DPT-3 vaccination, measles-1 vaccination, drinking water source, sanitation facility, household wealth, education level of the household head | Child's age, sex, birthweight, healthcard ownership, receipt of vitamin A, DPT-3 vaccination, measles-1 vaccination, main household cooking fuel, household crowding, household wealth, education level of the household head | Child's age, sex, birthweight, healthcard ownership, DPT-3 vaccination, measles-1 vaccination, ITN use, drinking water source, sanitation facility, household wealth, education level of the household head | Child's age, sex, birthweight, healthcard ownership, DPT-3 vaccination, measles-1 vaccination, ITN use, drinking water source, sanitation facility, household wealth, education level of the household head | Child's age, sex, birthweight, healthcard ownership, DPT-3 vaccination, measles-1 vaccination, ITN use, drinking water source, sanitation facility, household wealth, education level of the household head | Child's age, sex, birthweight, ITN use, healthcard ownership, receipt of vitamin A, DPT-3 vaccination, measles-1 vaccination, drinking water source, sanitation facility, household wealth, education level of the household head |
| Malawi 2012 MIS | Child's age, sex, ITN use, IRS in the past 12 months, household wealth | Child's age, sex, ITN use, IRS in the past 12 months, household wealth | - | - | - | - | - | - |
| Malawi 2014 MIS | Child's age, sex, ITN use, IRS in the past 12 months, household wealth | Child's age, sex, ITN use, IRS in the past 12 months, household wealth | - | - | - | - | - | - |

**Table A.** Surveys included (n=77) and covariables adjusted for in the analysis of house construction materials.

| **Survey** | **Malaria infection (microscopy)** | **Malaria infection (RDT)** | **Diarrhoea** | **Acute respiratory infection** | **Low height-for-age** | **Low weight-for-height** | **Low weight-for-age** | **Anaemia** |
|---|---|---|---|---|---|---|---|---|
| Malawi 2015 DHS | - | - | Child's age, sex, birthweight, healthcard ownership, receipt of vitamin A, DPT-3 vaccination, measles-1 vaccination, rotavirus-2 vaccination, drinking water source, sanitation facility, household wealth, education level of the household head | Child's age, sex, birthweight, healthcard ownership, receipt of vitamin A, DPT-3 vaccination, measles-1 vaccination, pneumococcal-3 vaccination, smoking in house, main household cooking fuel, household crowding, household wealth, education level of the household head | Child's age, sex, birthweight, healthcard ownership, DPT-3 vaccination, measles-1 vaccination, ITN use, drinking water source, sanitation facility, household wealth, education level of the household head | Child's age, sex, birthweight, healthcard ownership, DPT-3 vaccination, measles-1 vaccination, ITN use, drinking water source, sanitation facility, household wealth, education level of the household head | Child's age, sex, birthweight, healthcard ownership, DPT-3 vaccination, measles-1 vaccination, ITN use, drinking water source, sanitation facility, household wealth, education level of the household head | Child's age, sex, birthweight, ITN use, healthcard ownership, receipt of vitamin A, DPT-3 vaccination, measles-1 vaccination, rotavirus-2 vaccination, pneumococcal-3 vaccination, drinking water source, sanitation facility, household wealth, education level of the household head |
| Malawi 2017 MIS | Child's age, sex, ITN use, household wealth | Child's age, sex, ITN use, household wealth | - | - | - | - | - | - |
| Mali 2012 DHS | Child's age, sex, ITN use, IRS in the past 12 months, household wealth, education level of the household head | Child's age, sex, ITN use, IRS in the past 12 months, household wealth, education level of the household head | Child's age, sex, birthweight, healthcard ownership, receipt of vitamin A, DPT-3 vaccination, measles-1 vaccination, drinking water source, sanitation facility, household wealth, education level of the household head | - | Child's age, sex, birthweight, healthcard ownership, DPT-3 vaccination, measles-1 vaccination, ITN use, drinking water source, sanitation facility, household wealth, education level of the household head | Child's age, sex, birthweight, healthcard ownership, DPT-3 vaccination, measles-1 vaccination, ITN use, drinking water source, sanitation facility, household wealth, education level of the household head | Child's age, sex, birthweight, healthcard ownership, DPT-3 vaccination, measles-1 vaccination, ITN use, drinking water source, sanitation facility, household wealth, education level of the household head | Child's age, sex, birthweight, ITN use, healthcard ownership, receipt of vitamin A, DPT-3 vaccination, measles-1 vaccination, drinking water source, sanitation facility, household wealth, education level of the household head |

**Table A.** Surveys included (n=77) and covariables adjusted for in the analysis of house construction materials.

| Survey | Malaria infection (microscopy) | Malaria infection (RDT) | Diarrhoea | Acute respiratory infection | Low height-for-age | Low weight-for-height | Low weight-for-age | Anaemia |
|---|---|---|---|---|---|---|---|---|
| Mali 2015 MIS | Child's age, sex, ITN use, IRS in the past 12 months, household wealth | Child's age, sex, ITN use, IRS in the past 12 months, household wealth | - | - | - | - | - | - |
| Mozambique 2011 DHS | Child's age, sex, ITN use, IRS in the past 12 months, household wealth, education level of the household head | Child's age, sex, ITN use, IRS in the past 12 months, household wealth, education level of the household head | Child's age, sex, birthweight, healthcard ownership, receipt of vitamin A, DPT-3 vaccination, measles-1 vaccination, drinking water source, sanitation facility, household wealth, education level of the household head | - | Child's age, sex, birthweight, healthcard ownership, DPT-3 vaccination, measles-1 vaccination, ITN use, drinking water source, sanitation facility, household wealth, education level of the household head | Child's age, sex, birthweight, healthcard ownership, DPT-3 vaccination, measles-1 vaccination, ITN use, drinking water source, sanitation facility, household wealth, education level of the household head | Child's age, sex, birthweight, healthcard ownership, DPT-3 vaccination, measles-1 vaccination, ITN use, drinking water source, sanitation facility, household wealth, education level of the household head | Child's age, sex, birthweight, ITN use, healthcard ownership, receipt of vitamin A, DPT-3 vaccination, measles-1 vaccination, drinking water source, sanitation facility, household wealth, education level of the household head |
| Mozambique 2015 AIS | - | Child's age, sex, ITN use, IRS in the past 12 months, household wealth, education level of the household head | - | - | - | - | - | - |

**Table A.** Surveys included (n=77) and covariables adjusted for in the analysis of house construction materials.

| Survey | Malaria infection (microscopy) | Malaria infection (RDT) | Diarrhoea | Acute respiratory infection | Low height-for-age | Low weight-for-height | Low weight-for-age | Anaemia |
|---|---|---|---|---|---|---|---|---|
| Namibia 2006 DHS | - | - | Child's age, sex, birthweight, healthcard ownership, receipt of vitamin A, DPT-3 vaccination, measles-1 vaccination, drinking water source, sanitation facility, household wealth, education level of the household head | Child's age, sex, birthweight, healthcard ownership, receipt of vitamin A, DPT-3 vaccination, measles-1 vaccination, main household cooking fuel, household crowding, household wealth, education level of the household head | Child's age, sex, birthweight, healthcard ownership, DPT-3 vaccination, measles-1 vaccination, ITN use, drinking water source, sanitation facility, household wealth, education level of the household head | Child's age, sex, birthweight, healthcard ownership, DPT-3 vaccination, measles-1 vaccination, ITN use, drinking water source, sanitation facility, household wealth, education level of the household head | Child's age, sex, birthweight, healthcard ownership, DPT-3 vaccination, measles-1 vaccination, ITN use, drinking water source, sanitation facility, household wealth, education level of the household head | - |
| Namibia 2013 DHS | - | - | Child's age, sex, birthweight, healthcard ownership, receipt of vitamin A, DPT-3 vaccination, measles-1 vaccination, drinking water source, sanitation facility, household wealth, education level of the household head | Child's age, sex, birthweight, healthcard ownership, receipt of vitamin A, DPT-3 vaccination, measles-1 vaccination, smoking in house, main household cooking fuel, household crowding, household wealth, education level of the household head | Child's age, sex, birthweight, healthcard ownership, DPT-3 vaccination, measles-1 vaccination, ITN use, drinking water source, sanitation facility, household wealth, education level of the household head | Child's age, sex, birthweight, healthcard ownership, DPT-3 vaccination, measles-1 vaccination, ITN use, drinking water source, sanitation facility, household wealth, education level of the household head | Child's age, sex, birthweight, healthcard ownership, DPT-3 vaccination, measles-1 vaccination, ITN use, drinking water source, sanitation facility, household wealth, education level of the household head | Child's age, sex, birthweight, ITN use, healthcard ownership, receipt of vitamin A, DPT-3 vaccination, measles-1 vaccination, drinking water source, sanitation facility, household wealth, education level of the household head |

**Table A.** Surveys included (n=77) and covariables adjusted for in the analysis of house construction materials.

| **Survey** | **Malaria infection (microscopy)** | **Malaria infection (RDT)** | **Diarrhoea** | **Acute respiratory infection** | **Low height-for-age** | **Low weight-for-height** | **Low weight-for-age** | **Anaemia** |
|---|---|---|---|---|---|---|---|---|
| Niger 2012 DHS | - | - | Child's age, sex, birthweight, healthcard ownership, receipt of vitamin A, DPT-3 vaccination, measles-1 vaccination, drinking water source, sanitation facility, household wealth, education level of the household head | Child's age, sex, birthweight, healthcard ownership, receipt of vitamin A, DPT-3 vaccination, measles-1 vaccination, main household cooking fuel, household crowding, household wealth, education level of the household head | Child's age, sex, birthweight, healthcard ownership, DPT-3 vaccination, measles-1 vaccination, ITN use, drinking water source, sanitation facility, household wealth, education level of the household head | Child's age, sex, birthweight, healthcard ownership, DPT-3 vaccination, measles-1 vaccination, ITN use, drinking water source, sanitation facility, household wealth, education level of the household head | Child's age, sex, birthweight, healthcard ownership, DPT-3 vaccination, measles-1 vaccination, ITN use, drinking water source, sanitation facility, household wealth, education level of the household head | Child's age, sex, birthweight, ITN use, healthcard ownership, receipt of vitamin A, DPT-3 vaccination, measles-1 vaccination, drinking water source, sanitation facility, household wealth, education level of the household head |
| Nigeria 2008 DHS | - | - | Child's age, sex, birthweight, healthcard ownership, receipt of vitamin A, DPT-3 vaccination, measles-1 vaccination, drinking water source, sanitation facility, household wealth, education level of the household head | - | Child's age, sex, birthweight, healthcard ownership, DPT-3 vaccination, measles-1 vaccination, ITN use, drinking water source, sanitation facility, household wealth, education level of the household head | Child's age, sex, birthweight, healthcard ownership, DPT-3 vaccination, measles-1 vaccination, ITN use, drinking water source, sanitation facility, household wealth, education level of the household head | Child's age, sex, birthweight, healthcard ownership, DPT-3 vaccination, measles-1 vaccination, ITN use, drinking water source, sanitation facility, household wealth, education level of the household head | - |
| Nigeria 2010 MIS | Child's age, sex, ITN use, IRS in the past 12 months, household wealth, education level of the household head | Child's age, sex, ITN use, IRS in the past 12 months, household wealth, education level of the household head | - | - | - | - | - | - |

**Table A.** Surveys included (n=77) and covariables adjusted for in the analysis of house construction materials.

| Survey | Malaria infection (microscopy) | Malaria infection (RDT) | Diarrhoea | Acute respiratory infection | Low height-for-age | Low weight-for-height | Low weight-for-age | Anaemia |
|---|---|---|---|---|---|---|---|---|
| Nigeria 2013 DHS | - | - | Child's age, sex, birthweight, healthcard ownership, receipt of vitamin A, DPT-3 vaccination, measles-1 vaccination, drinking water source, sanitation facility, household wealth, education level of the household head | - | Child's age, sex, birthweight, healthcard ownership, DPT-3 vaccination, measles-1 vaccination, ITN use, drinking water source, sanitation facility, household wealth, education level of the household head | Child's age, sex, birthweight, healthcard ownership, DPT-3 vaccination, measles-1 vaccination, ITN use, drinking water source, sanitation facility, household wealth, education level of the household head | Child's age, sex, birthweight, healthcard ownership, DPT-3 vaccination, measles-1 vaccination, ITN use, drinking water source, sanitation facility, household wealth, education level of the household head | - |
| Nigeria 2015 MIS | Child's age, sex, ITN use, IRS in the past 12 months, household wealth, education level of the household head | Child's age, sex, ITN use, IRS in the past 12 months, household wealth, education level of the household head | - | - | - | - | - | - |
| Rwanda 2010 DHS | Child's age, sex, ITN use, household wealth, education level of the household head | Child's age, sex, ITN use, household wealth, education level of the household head | Child's age, sex, birthweight, healthcard ownership, receipt of vitamin A, DPT-3 vaccination, measles-1 vaccination, drinking water source, sanitation facility, household wealth, education level of the household head | Child's age, sex, birthweight, healthcard ownership, receipt of vitamin A, DPT-3 vaccination, measles-1 vaccination, smoking in house, main household cooking fuel, household crowding, household wealth, education level of the household head | Child's age, sex, birthweight, healthcard ownership, DPT-3 vaccination, measles-1 vaccination, ITN use, drinking water source, sanitation facility, household wealth, education level of the household head | Child's age, sex, birthweight, healthcard ownership, DPT-3 vaccination, measles-1 vaccination, ITN use, drinking water source, sanitation facility, household wealth, education level of the household head | Child's age, sex, birthweight, healthcard ownership, DPT-3 vaccination, measles-1 vaccination, ITN use, drinking water source, sanitation facility, household wealth, education level of the household head | Child's age, sex, birthweight, ITN use, healthcard ownership, receipt of vitamin A, DPT-3 vaccination, measles-1 vaccination, drinking water source, sanitation facility, household wealth, education level of the household head |

**Table A.** Surveys included (n=77) and covariables adjusted for in the analysis of house construction materials.

| Survey | Malaria infection (microscopy) | Malaria infection (RDT) | Diarrhoea | Acute respiratory infection | Low height-for-age | Low weight-for-height | Low weight-for-age | Anaemia |
|---|---|---|---|---|---|---|---|---|
| Rwanda 2015 DHS | Child's age, sex, ITN use, household wealth, education level of the household head | Child's age, sex, ITN use, household wealth, education level of the household head | Child's age, sex, birthweight, healthcard ownership, receipt of vitamin A, DPT-3 vaccination, measles-1 vaccination, rotavirus-2 vaccination, drinking water source, sanitation facility, household wealth, education level of the household head | Child's age, sex, birthweight, healthcard ownership, receipt of vitamin A, DPT-3 vaccination, measles-1 vaccination, smoking in house, main household cooking fuel, household crowding, household wealth, education level of the household head | Child's age, sex, birthweight, healthcard ownership, DPT-3 vaccination, measles-1 vaccination, ITN use, drinking water source, sanitation facility, household wealth, education level of the household head | Child's age, sex, birthweight, healthcard ownership, DPT-3 vaccination, measles-1 vaccination, ITN use, drinking water source, sanitation facility, household wealth, education level of the household head | Child's age, sex, birthweight, healthcard ownership, DPT-3 vaccination, measles-1 vaccination, ITN use, drinking water source, sanitation facility, household wealth, education level of the household head | Child's age, sex, birthweight, ITN use, healthcard ownership, receipt of vitamin A, DPT-3 vaccination, measles-1 vaccination, rotavirus-2 vaccination, drinking water source, sanitation facility, household wealth, education level of the household head |
| Rwanda 2017 MIS | Child's age, sex, ITN use, IRS in the past 12 months, household wealth | Child's age, sex, ITN use, IRS in the past 12 months, household wealth | - | - | - | - | - | - |
| Senegal 2008 MIS | Child's age, sex, ITN use, household wealth | Child's age, sex, ITN use, household wealth | - | - | - | - | - | - |

**Table A.** Surveys included (n=77) and covariables adjusted for in the analysis of house construction materials.

| Survey | Malaria infection (microscopy) | Malaria infection (RDT) | Diarrhoea | Acute respiratory infection | Low height-for-age | Low weight-for-height | Low weight-for-age | Anaemia |
|---|---|---|---|---|---|---|---|---|
| Senegal 2010 DHS | Child's age, sex, ITN use, IRS in the past 12 months, household wealth, education level of the household head | Child's age, sex, ITN use, IRS in the past 12 months, household wealth, education level of the household head | Child's age, sex, birthweight, healthcard ownership, receipt of vitamin A, DPT-3 vaccination, measles-1 vaccination, drinking water source, sanitation facility, household wealth, education level of the household head | Child's age, sex, birthweight, healthcard ownership, receipt of vitamin A, DPT-3 vaccination, measles-1 vaccination, main household cooking fuel, household crowding, household wealth, education level of the household head | Child's age, sex, birthweight, healthcard ownership, DPT-3 vaccination, measles-1 vaccination, ITN use, drinking water source, sanitation facility, household wealth, education level of the household head | Child's age, sex, birthweight, healthcard ownership, DPT-3 vaccination, measles-1 vaccination, ITN use, drinking water source, sanitation facility, household wealth, education level of the household head | Child's age, sex, birthweight, healthcard ownership, DPT-3 vaccination, measles-1 vaccination, ITN use, drinking water source, sanitation facility, household wealth, education level of the household head | Child's age, sex, birthweight, ITN use, healthcard ownership, receipt of vitamin A, DPT-3 vaccination, measles-1 vaccination, drinking water source, sanitation facility, household wealth, education level of the household head |
| Senegal 2012 DHS | Child's age, sex, ITN use, IRS in the past 12 months, household wealth, education level of the household head | Child's age, sex, ITN use, IRS in the past 12 months, household wealth, education level of the household head | Child's age, sex, birthweight, healthcard ownership, receipt of vitamin A, DPT-3 vaccination, measles-1 vaccination, drinking water source, sanitation facility, household wealth, education level of the household head | - | Child's age, sex, birthweight, healthcard ownership, DPT-3 vaccination, measles-1 vaccination, ITN use, drinking water source, sanitation facility, household wealth, education level of the household head | Child's age, sex, birthweight, healthcard ownership, DPT-3 vaccination, measles-1 vaccination, ITN use, drinking water source, sanitation facility, household wealth, education level of the household head | Child's age, sex, birthweight, healthcard ownership, DPT-3 vaccination, measles-1 vaccination, ITN use, drinking water source, sanitation facility, household wealth, education level of the household head | Child's age, sex, birthweight, ITN use, healthcard ownership, receipt of vitamin A, DPT-3 vaccination, measles-1 vaccination, drinking water source, sanitation facility, household wealth, education level of the household head |

**Table A.** Surveys included (n=77) and covariables adjusted for in the analysis of house construction materials.

| Survey | Malaria infection (microscopy) | Malaria infection (RDT) | Diarrhoea | Acute respiratory infection | Low height-for-age | Low weight-for-height | Low weight-for-age | Anaemia |
|---|---|---|---|---|---|---|---|---|
| Senegal 2014 DHS | Child's age, sex, ITN use, IRS in the past 12 months, household wealth, education level of the household head | Child's age, sex, ITN use, IRS in the past 12 months, household wealth, education level of the household head | Child's age, sex, birthweight, healthcard ownership, receipt of vitamin A, DPT-3 vaccination, measles-1 vaccination, drinking water source, sanitation facility, household wealth, education level of the household head | - | Child's age, sex, birthweight, healthcard ownership, DPT-3 vaccination, measles-1 vaccination, ITN use, drinking water source, sanitation facility, household wealth, education level of the household head | Child's age, sex, birthweight, healthcard ownership, DPT-3 vaccination, measles-1 vaccination, ITN use, drinking water source, sanitation facility, household wealth, education level of the household head | Child's age, sex, birthweight, healthcard ownership, DPT-3 vaccination, measles-1 vaccination, ITN use, drinking water source, sanitation facility, household wealth, education level of the household head | Child's age, sex, birthweight, ITN use, healthcard ownership, receipt of vitamin A, DPT-3 vaccination, measles-1 vaccination, drinking water source, sanitation facility, household wealth, education level of the household head |
| Senegal 2015 DHS | Child's age, sex, ITN use, IRS in the past 12 months, household wealth, education level of the household head | Child's age, sex, ITN use, IRS in the past 12 months, household wealth, education level of the household head | Child's age, sex, birthweight, healthcard ownership, receipt of vitamin A, DPT-3 vaccination, measles-1 vaccination, drinking water source, sanitation facility, household wealth, education level of the household head | - | Child's age, sex, birthweight, healthcard ownership, DPT-3 vaccination, measles-1 vaccination, ITN use, drinking water source, sanitation facility, household wealth, education level of the household head | Child's age, sex, birthweight, healthcard ownership, DPT-3 vaccination, measles-1 vaccination, ITN use, drinking water source, sanitation facility, household wealth, education level of the household head | Child's age, sex, birthweight, healthcard ownership, DPT-3 vaccination, measles-1 vaccination, ITN use, drinking water source, sanitation facility, household wealth, education level of the household head | Child's age, sex, birthweight, ITN use, healthcard ownership, receipt of vitamin A, DPT-3 vaccination, measles-1 vaccination, drinking water source, sanitation facility, household wealth, education level of the household head |

**Table A.** Surveys included (n=77) and covariables adjusted for in the analysis of house construction materials.

| Survey | Malaria infection (microscopy) | Malaria infection (RDT) | Diarrhoea | Acute respiratory infection | Low height-for-age | Low weight-for-height | Low weight-for-age | Anaemia |
|---|---|---|---|---|---|---|---|---|
| Senegal 2016 DHS | Child's age, sex, ITN use, IRS in the past 12 months, household wealth, education level of the household head | Child's age, sex, ITN use, IRS in the past 12 months, household wealth, education level of the household head | Child's age, sex, birthweight, healthcard ownership, receipt of vitamin A, DPT-3 vaccination, measles-1 vaccination, drinking water source, sanitation facility, household wealth, education level of the household head | - | Child's age, sex, birthweight, healthcard ownership, DPT-3 vaccination, measles-1 vaccination, ITN use, drinking water source, sanitation facility, household wealth, education level of the household head | Child's age, sex, birthweight, healthcard ownership, DPT-3 vaccination, measles-1 vaccination, ITN use, drinking water source, sanitation facility, household wealth, education level of the household head | Child's age, sex, birthweight, healthcard ownership, DPT-3 vaccination, measles-1 vaccination, ITN use, drinking water source, sanitation facility, household wealth, education level of the household head | Child's age, sex, birthweight, ITN use, healthcard ownership, receipt of vitamin A, DPT-3 vaccination, measles-1 vaccination, drinking water source, sanitation facility, household wealth, education level of the household head |
| Sierra Leone 2008 DHS | - | - | Child's age, sex, birthweight, healthcard ownership, receipt of vitamin A, DPT-3 vaccination, measles-1 vaccination, drinking water source, sanitation facility, household wealth, education level of the household head | - | Child's age, sex, birthweight, healthcard ownership, DPT-3 vaccination, measles-1 vaccination, ITN use, drinking water source, sanitation facility, household wealth, education level of the household head | Child's age, sex, birthweight, healthcard ownership, DPT-3 vaccination, measles-1 vaccination, ITN use, drinking water source, sanitation facility, household wealth, education level of the household head | Child's age, sex, birthweight, healthcard ownership, DPT-3 vaccination, measles-1 vaccination, ITN use, drinking water source, sanitation facility, household wealth, education level of the household head | Child's age, sex, birthweight, ITN use, healthcard ownership, receipt of vitamin A, DPT-3 vaccination, measles-1 vaccination, drinking water source, sanitation facility, household wealth, education level of the household head |

**Table A.** Surveys included (n=77) and covariables adjusted for in the analysis of house construction materials.

| Survey | Malaria infection (microscopy) | Malaria infection (RDT) | Diarrhoea | Acute respiratory infection | Low height-for-age | Low weight-for-height | Low weight-for-age | Anaemia |
|---|---|---|---|---|---|---|---|---|
| Sierra Leone 2013 DHS | - | - | Child's age, sex, birthweight, healthcard ownership, receipt of vitamin A, DPT-3 vaccination, measles-1 vaccination, drinking water source, sanitation facility, household wealth, education level of the household head | Child's age, sex, birthweight, healthcard ownership, receipt of vitamin A, DPT-3 vaccination, measles-1 vaccination, smoking in house, main household cooking fuel, household crowding, household wealth, education level of the household head | Child's age, sex, birthweight, healthcard ownership, DPT-3 vaccination, measles-1 vaccination, ITN use, drinking water source, sanitation facility, household wealth, education level of the household head | Child's age, sex, birthweight, healthcard ownership, DPT-3 vaccination, measles-1 vaccination, ITN use, drinking water source, sanitation facility, household wealth, education level of the household head | Child's age, sex, birthweight, healthcard ownership, DPT-3 vaccination, measles-1 vaccination, ITN use, drinking water source, sanitation facility, household wealth, education level of the household head | Child's age, sex, birthweight, ITN use, healthcard ownership, receipt of vitamin A, DPT-3 vaccination, measles-1 vaccination, drinking water source, sanitation facility, household wealth, education level of the household head |
| Sierra Leone 2016 MIS | Child's age, sex, ITN use, IRS in the past 12 months, household wealth | Child's age, sex, ITN use, IRS in the past 12 months, household wealth | - | - | - | - | - | - |
| Tanzania 2004 DHS | - | - | Child's age, sex, birthweight, healthcard ownership, receipt of vitamin A, DPT-3 vaccination, measles-1 vaccination, drinking water source, sanitation facility, household wealth, education level of the household head | - | Child's age, sex, birthweight, healthcard ownership, DPT-3 vaccination, measles-1 vaccination, ITN use, drinking water source, sanitation facility, household wealth, education level of the household head | Child's age, sex, birthweight, healthcard ownership, DPT-3 vaccination, measles-1 vaccination, ITN use, drinking water source, sanitation facility, household wealth, education level of the household head | Child's age, sex, birthweight, healthcard ownership, DPT-3 vaccination, measles-1 vaccination, ITN use, drinking water source, sanitation facility, household wealth, education level of the household head | Child's age, sex, birthweight, ITN use, healthcard ownership, receipt of vitamin A, DPT-3 vaccination, measles-1 vaccination, drinking water source, sanitation facility, household wealth, education level of the household head |

**Table A.** Surveys included (n=77) and covariables adjusted for in the analysis of house construction materials.

| Survey | Malaria infection (microscopy) | Malaria infection (RDT) | Diarrhoea | Acute respiratory infection | Low height-for-age | Low weight-for-height | Low weight-for-age | Anaemia |
|---|---|---|---|---|---|---|---|---|
| Tanzania 2010 DHS | - | - | Child's age, sex, birthweight, healthcard ownership, receipt of vitamin A, DPT-3 vaccination, measles-1 vaccination, drinking water source, sanitation facility, household wealth, education level of the household head | Child's age, sex, birthweight, healthcard ownership, receipt of vitamin A, DPT-3 vaccination, measles-1 vaccination, main household cooking fuel, household crowding, household wealth, education level of the household head | Child's age, sex, birthweight, healthcard ownership, DPT-3 vaccination, measles-1 vaccination, ITN use, drinking water source, sanitation facility, household wealth, education level of the household head | Child's age, sex, birthweight, healthcard ownership, DPT-3 vaccination, measles-1 vaccination, ITN use, drinking water source, sanitation facility, household wealth, education level of the household head | Child's age, sex, birthweight, healthcard ownership, DPT-3 vaccination, measles-1 vaccination, ITN use, drinking water source, sanitation facility, household wealth, education level of the household head | Child's age, sex, birthweight, ITN use, healthcard ownership, receipt of vitamin A, DPT-3 vaccination, measles-1 vaccination, drinking water source, sanitation facility, household wealth, education level of the household head |
| Tanzania 2012 AIS | Child's age, sex, ITN use, IRS in the past 12 months, household wealth, education level of the household head | Child's age, sex, ITN use, IRS in the past 12 months, household wealth, education level of the household head | - | - | - | - | - | - |
| Tanzania 2017 MIS | - | Child's age, sex, ITN use, household wealth | - | - | - | - | - | - |

**Table A.** Surveys included (n=77) and covariables adjusted for in the analysis of house construction materials.

| Survey | Malaria infection (microscopy) | Malaria infection (RDT) | Diarrhoea | Acute respiratory infection | Low height-for-age | Low weight-for-height | Low weight-for-age | Anaemia |
|---|---|---|---|---|---|---|---|---|
| Togo 2013 DHS | Child's age, sex, ITN use, household wealth, education level of the household head | Child's age, sex, ITN use, household wealth, education level of the household head | Child's age, sex, birthweight, healthcard ownership, receipt of vitamin A, DPT-3 vaccination, measles-1 vaccination, drinking water source, sanitation facility, household wealth, education level of the household head | Child's age, sex, birthweight, healthcard ownership, receipt of vitamin A, DPT-3 vaccination, measles-1 vaccination, smoking in house, main household cooking fuel, household crowding, household wealth, education level of the household head | Child's age, sex, birthweight, healthcard ownership, DPT-3 vaccination, measles-1 vaccination, ITN use, drinking water source, sanitation facility, household wealth, education level of the household head | Child's age, sex, birthweight, healthcard ownership, DPT-3 vaccination, measles-1 vaccination, ITN use, drinking water source, sanitation facility, household wealth, education level of the household head | Child's age, sex, birthweight, healthcard ownership, DPT-3 vaccination, measles-1 vaccination, ITN use, drinking water source, sanitation facility, household wealth, education level of the household head | Child's age, sex, birthweight, ITN use, healthcard ownership, receipt of vitamin A, DPT-3 vaccination, measles-1 vaccination, drinking water source, sanitation facility, household wealth, education level of the household head |
| Uganda 2006 DHS | - | - | Child's age, sex, birthweight, healthcard ownership, receipt of vitamin A, DPT-3 vaccination, measles-1 vaccination, drinking water source, sanitation facility, household wealth, education level of the household head | Child's age, sex, birthweight, healthcard ownership, receipt of vitamin A, DPT-3 vaccination, measles-1 vaccination, main household cooking fuel, household crowding, household wealth, education level of the household head | Child's age, sex, birthweight, healthcard ownership, DPT-3 vaccination, measles-1 vaccination, ITN use, drinking water source, sanitation facility, household wealth, education level of the household head | Child's age, sex, birthweight, healthcard ownership, DPT-3 vaccination, measles-1 vaccination, ITN use, drinking water source, sanitation facility, household wealth, education level of the household head | Child's age, sex, birthweight, healthcard ownership, DPT-3 vaccination, measles-1 vaccination, ITN use, drinking water source, sanitation facility, household wealth, education level of the household head | Child's age, sex, birthweight, ITN use, healthcard ownership, receipt of vitamin A, DPT-3 vaccination, measles-1 vaccination, drinking water source, sanitation facility, household wealth, education level of the household head |
| Uganda 2009 MIS | Child's age, sex, ITN use, household wealth | Child's age, sex, ITN use, household wealth | - | - | - | - | - | - |

**Table A.** Surveys included (n=77) and covariables adjusted for in the analysis of house construction materials.

| Survey | Malaria infection (microscopy) | Malaria infection (RDT) | Diarrhoea | Acute respiratory infection | Low height-for-age | Low weight-for-height | Low weight-for-age | Anaemia |
|---|---|---|---|---|---|---|---|---|
| Uganda 2014 MIS | Child's age, sex, ITN use, IRS in the past 12 months, household wealth | Child's age, sex, ITN use, IRS in the past 12 months, household wealth | - | - | - | - | - | - |
| Uganda 2016 DHS | - | Child's age, sex, ITN use, household wealth, education level of the household head | Child's age, sex, birthweight, healthcard ownership, receipt of vitamin A, DPT-3 vaccination, measles-1 vaccination, rotavirus-2 vaccination, drinking water source, sanitation facility, household wealth, education level of the household head | Child's age, sex, birthweight, healthcard ownership, receipt of vitamin A, DPT-3 vaccination, measles-1 vaccination, pneumococcal-3 vaccination, smoking in house, main household cooking fuel, household crowding, household wealth, education level of the household head | Child's age, sex, birthweight, healthcard ownership, DPT-3 vaccination, measles-1 vaccination, ITN use, drinking water source, sanitation facility, household wealth, education level of the household head | Child's age, sex, birthweight, healthcard ownership, DPT-3 vaccination, measles-1 vaccination, ITN use, drinking water source, sanitation facility, household wealth, education level of the household head | Child's age, sex, birthweight, healthcard ownership, DPT-3 vaccination, measles-1 vaccination, ITN use, drinking water source, sanitation facility, household wealth, education level of the household head | Child's age, sex, birthweight, ITN use, healthcard ownership, receipt of vitamin A, DPT-3 vaccination, measles-1 vaccination, rotavirus-2 vaccination, pneumococcal-3 vaccination, drinking water source, sanitation facility, household wealth, education level of the household head |
| Zambia 2007 DHS | - | - | Child's age, sex, birthweight, healthcard ownership, receipt of vitamin A, DPT-3 vaccination, measles-1 vaccination, drinking water source, sanitation facility, household wealth, education level of the household head | Child's age, sex, birthweight, healthcard ownership, receipt of vitamin A, DPT-3 vaccination, measles-1 vaccination, main household cooking fuel, household crowding, household wealth, education level of the household head | Child's age, sex, birthweight, healthcard ownership, DPT-3 vaccination, measles-1 vaccination, ITN use, drinking water source, sanitation facility, household wealth, education level of the household head | Child's age, sex, birthweight, healthcard ownership, DPT-3 vaccination, measles-1 vaccination, ITN use, drinking water source, sanitation facility, household wealth, education level of the household head | Child's age, sex, birthweight, healthcard ownership, DPT-3 vaccination, measles-1 vaccination, ITN use, drinking water source, sanitation facility, household wealth, education level of the household head | - |

**Table A.** Surveys included (n=77) and covariables adjusted for in the analysis of house construction materials.

| Survey | Malaria infection (microscopy) | Malaria infection (RDT) | Diarrhoea | Acute respiratory infection | Low height-for-age | Low weight-for-height | Low weight-for-age | Anaemia |
|---|---|---|---|---|---|---|---|---|
| Zambia 2013 DHS | - | - | Child's age, sex, birthweight, healthcard ownership, receipt of vitamin A, DPT-3 vaccination, measles-1 vaccination, drinking water source, sanitation facility, household wealth, education level of the household head | Child's age, sex, birthweight, healthcard ownership, receipt of vitamin A, DPT-3 vaccination, measles-1 vaccination, smoking in house, main household cooking fuel, household crowding, household wealth, education level of the household head | Child's age, sex, birthweight, healthcard ownership, DPT-3 vaccination, measles-1 vaccination, ITN use, drinking water source, sanitation facility, household wealth, education level of the household head | Child's age, sex, birthweight, healthcard ownership, DPT-3 vaccination, measles-1 vaccination, ITN use, drinking water source, sanitation facility, household wealth, education level of the household head | Child's age, sex, birthweight, healthcard ownership, DPT-3 vaccination, measles-1 vaccination, ITN use, drinking water source, sanitation facility, household wealth, education level of the household head | - |
| Zimbabwe 2005 DHS | - | - | Child's age, sex, birthweight, healthcard ownership, receipt of vitamin A, DPT-3 vaccination, measles-1 vaccination, drinking water source, sanitation facility, household wealth, education level of the household head | Child's age, sex, birthweight, healthcard ownership, receipt of vitamin A, DPT-3 vaccination, measles-1 vaccination, main household cooking fuel, household crowding, household wealth, education level of the household head | Child's age, sex, birthweight, healthcard ownership, DPT-3 vaccination, measles-1 vaccination, ITN use, drinking water source, sanitation facility, household wealth, education level of the household head | Child's age, sex, birthweight, healthcard ownership, DPT-3 vaccination, measles-1 vaccination, ITN use, drinking water source, sanitation facility, household wealth, education level of the household head | Child's age, sex, birthweight, healthcard ownership, DPT-3 vaccination, measles-1 vaccination, ITN use, drinking water source, sanitation facility, household wealth, education level of the household head | Child's age, sex, birthweight, ITN use, healthcard ownership, receipt of vitamin A, DPT-3 vaccination, measles-1 vaccination, drinking water source, sanitation facility, household wealth, education level of the household head |

**Table A.** Surveys included (n=77) and covariables adjusted for in the analysis of house construction materials.

| Survey | Malaria infection (microscopy) | Malaria infection (RDT) | Diarrhoea | Acute respiratory infection | Low height-for-age | Low weight-for-height | Low weight-for-age | Anaemia |
|---|---|---|---|---|---|---|---|---|
| Zimbabwe 2010 DHS | - | - | Child's age, sex, birthweight, healthcard ownership, receipt of vitamin A, DPT-3 vaccination, measles-1 vaccination, drinking water source, sanitation facility, household wealth, education level of the household head | Child's age, sex, birthweight, healthcard ownership, receipt of vitamin A, DPT-3 vaccination, measles-1 vaccination, smoking in house, main household cooking fuel, household crowding, household wealth, education level of the household head | Child's age, sex, birthweight, healthcard ownership, DPT-3 vaccination, measles-1 vaccination, ITN use, drinking water source, sanitation facility, household wealth, education level of the household head | Child's age, sex, birthweight, healthcard ownership, DPT-3 vaccination, measles-1 vaccination, ITN use, drinking water source, sanitation facility, household wealth, education level of the household head | Child's age, sex, birthweight, healthcard ownership, DPT-3 vaccination, measles-1 vaccination, ITN use, drinking water source, sanitation facility, household wealth, education level of the household head | Child's age, sex, birthweight, ITN use, healthcard ownership, receipt of vitamin A, DPT-3 vaccination, measles-1 vaccination, drinking water source, sanitation facility, household wealth, education level of the household head |
| Zimbabwe 2015 DHS | - | - | Child's age, sex, birthweight, healthcard ownership, receipt of vitamin A, DPT-3 vaccination, measles-1 vaccination, rotavirus-2 vaccination, drinking water source, sanitation facility, household wealth, education level of the household head | Child's age, sex, birthweight, healthcard ownership, receipt of vitamin A, DPT-3 vaccination, measles-1 vaccination, pneumococcal-3 vaccination, smoking in house, main household cooking fuel, household crowding, household wealth, education level of the household head | Child's age, sex, birthweight, healthcard ownership, DPT-3 vaccination, measles-1 vaccination, ITN use, drinking water source, sanitation facility, household wealth, education level of the household head | Child's age, sex, birthweight, healthcard ownership, DPT-3 vaccination, measles-1 vaccination, ITN use, drinking water source, sanitation facility, household wealth, education level of the household head | Child's age, sex, birthweight, healthcard ownership, DPT-3 vaccination, measles-1 vaccination, ITN use, drinking water source, sanitation facility, household wealth, education level of the household head | Child's age, sex, birthweight, ITN use, healthcard ownership, receipt of vitamin A, DPT-3 vaccination, measles-1 vaccination, rotavirus-2 vaccination, pneumococcal-3 vaccination, drinking water source, sanitation facility, household wealth, education level of the household head |

Birthweight was modelled as a categorical variable comparing normal (2500g or greater) with low (less than 2500g) birthweight.
DPT: diphtheria-pertussis-tetanus; ITN: insecticide-treated net; RDT: rapid diagnostic test.

**Table B.** Surveys included (n=74) and covariables adjusted for in the analysis of house type

| Survey | Malaria infection (microscopy) | Malaria infection (RDT) | Diarrhoea | Acute respiratory infection | Low height-for-age | Low weight-for-height | Low weight-for-age | Anaemia |
|---|---|---|---|---|---|---|---|---|
| Angola 2011 MIS | Child's age, sex, ITN use, household wealth | Child's age, sex, ITN use, household wealth | - | - | - | - | - | - |
| Angola 2015 DHS | - | Child's age, sex, ITN use, IRS in the past 12 months, household wealth, education level of the household head | - | - | Child's age, sex, birthweight, healthcard ownership, DPT-3 vaccination, measles-1 vaccination, ITN use, household wealth, education level of the household head | Child's age, sex, birthweight, healthcard ownership, DPT-3 vaccination, measles-1 vaccination, ITN use, household wealth, education level of the household head | Child's age, sex, birthweight, healthcard ownership, DPT-3 vaccination, measles-1 vaccination, ITN use, household wealth, education level of the household head | - |
| Benin 2006 DHS | - | - | Child's age, sex, birthweight, healthcard ownership, receipt of vitamin A, DPT-3 vaccination, measles-1 vaccination, household wealth, education level of the household head | Child's age, sex, birthweight, healthcard ownership, receipt of vitamin A, DPT-3 vaccination, measles-1 vaccination, main household cooking fuel, household wealth, education level of the household head | Child's age, sex, birthweight, healthcard ownership, DPT-3 vaccination, measles-1 vaccination, ITN use, household wealth, education level of the household head | Child's age, sex, birthweight, healthcard ownership, DPT-3 vaccination, measles-1 vaccination, ITN use, household wealth, education level of the household head | Child's age, sex, birthweight, healthcard ownership, DPT-3 vaccination, measles-1 vaccination, ITN use, household wealth, education level of the household head | Child's age, sex, birthweight, ITN use, healthcard ownership, receipt of vitamin A, DPT-3 vaccination, measles-1 vaccination, household wealth, education level of the household head |
| Benin 2012 DHS | Child's age, sex, ITN use, IRS in the past 12 months, household wealth, education level of the household head | Child's age, sex, ITN use, IRS in the past 12 months, household wealth, education level of the household head | Child's age, sex, birthweight, healthcard ownership, receipt of vitamin A, DPT-3 vaccination, measles-1 vaccination, household wealth, education level of the household head | - | Child's age, sex, birthweight, healthcard ownership, DPT-3 vaccination, measles-1 vaccination, ITN use, household wealth, education level of the household head | Child's age, sex, birthweight, healthcard ownership, DPT-3 vaccination, measles-1 vaccination, ITN use, household wealth, education level of the household head | Child's age, sex, birthweight, healthcard ownership, DPT-3 vaccination, measles-1 vaccination, ITN use, household wealth, education level of the household head | Child's age, sex, birthweight, ITN use, healthcard ownership, receipt of vitamin A, DPT-3 vaccination, measles-1 vaccination, household wealth, education level of the household head |

**Table B.** Surveys included (n=74) and covariables adjusted for in the analysis of house type

| **Survey** | **Malaria infection (microscopy)** | **Malaria infection (RDT)** | **Diarrhoea** | **Acute respiratory infection** | **Low height-for-age** | **Low weight-for-height** | **Low weight-for-age** | **Anaemia** |
|---|---|---|---|---|---|---|---|---|
| Burkina Faso 2010 DHS | Child's age, sex, ITN use, IRS in the past 12 months, household wealth, education level of the household head | Child's age, sex, ITN use, IRS in the past 12 months, household wealth, education level of the household head | Child's age, sex, birthweight, healthcard ownership, receipt of vitamin A, DPT-3 vaccination, measles-1 vaccination, household wealth, education level of the household head | - | Child's age, sex, birthweight, healthcard ownership, DPT-3 vaccination, measles-1 vaccination, ITN use, household wealth, education level of the household head | Child's age, sex, birthweight, healthcard ownership, DPT-3 vaccination, measles-1 vaccination, ITN use, household wealth, education level of the household head | Child's age, sex, birthweight, healthcard ownership, DPT-3 vaccination, measles-1 vaccination, ITN use, household wealth, education level of the household head | Child's age, sex, birthweight, ITN use, healthcard ownership, receipt of vitamin A, DPT-3 vaccination, measles-1 vaccination, household wealth, education level of the household head |
| Burkina Faso 2014 MIS | Child's age, sex, ITN use, IRS in the past 12 months, household wealth | Child's age, sex, ITN use, IRS in the past 12 months, household wealth | - | - | - | - | - | - |
| Burundi 2010 DHS | - | - | Child's age, sex, birthweight, healthcard ownership, receipt of vitamin A, DPT-3 vaccination, measles-1 vaccination, household wealth, education level of the household head | Child's age, sex, birthweight, healthcard ownership, receipt of vitamin A, DPT-3 vaccination, measles-1 vaccination, smoking in house, main household cooking fuel, household wealth, education level of the household head | Child's age, sex, birthweight, healthcard ownership, DPT-3 vaccination, measles-1 vaccination, ITN use, household wealth, education level of the household head | Child's age, sex, birthweight, healthcard ownership, DPT-3 vaccination, measles-1 vaccination, ITN use, household wealth, education level of the household head | Child's age, sex, birthweight, healthcard ownership, DPT-3 vaccination, measles-1 vaccination, ITN use, household wealth, education level of the household head | Child's age, sex, birthweight, ITN use, healthcard ownership, receipt of vitamin A, DPT-3 vaccination, measles-1 vaccination, household wealth, education level of the household head |
| Burundi 2012 MIS | Child's age, sex, ITN use, IRS in the past 12 months, household wealth, education level of the household head | Child's age, sex, ITN use, IRS in the past 12 months, household wealth, education level of the household head | - | - | - | - | - | - |

**Table B.** Surveys included (n=74) and covariables adjusted for in the analysis of house type

| Survey | Malaria infection (microscopy) | Malaria infection (RDT) | Diarrhoea | Acute respiratory infection | Low height-for-age | Low weight-for-height | Low weight-for-age | Anaemia |
|---|---|---|---|---|---|---|---|---|
| Burundi 2016 DHS | Child's age, sex, ITN use, IRS in the past 12 months, household wealth, education level of the household head | Child's age, sex, ITN use, IRS in the past 12 months, household wealth, education level of the household head | Child's age, sex, birthweight, healthcard ownership, receipt of vitamin A, DPT-3 vaccination, measles-1 vaccination, rotavirus-2 vaccination, household wealth, education level of the household head | Child's age, sex, birthweight, healthcard ownership, receipt of vitamin A, DPT-3 vaccination, measles-1 vaccination, pneumococcal-3 vaccination, smoking in house, main household cooking fuel, household wealth, education level of the household head | Child's age, sex, birthweight, healthcard ownership, DPT-3 vaccination, measles-1 vaccination, ITN use, household wealth, education level of the household head | Child's age, sex, birthweight, healthcard ownership, DPT-3 vaccination, measles-1 vaccination, ITN use, household wealth, education level of the household head | Child's age, sex, birthweight, healthcard ownership, DPT-3 vaccination, measles-1 vaccination, ITN use, household wealth, education level of the household head | Child's age, sex, birthweight, ITN use, healthcard ownership, receipt of vitamin A, DPT-3 vaccination, measles-1 vaccination, rotavirus-2 vaccination, pneumococcal-3 vaccination, household wealth, education level of the household head |
| Cameroon 2011 DHS | - | Child's age, sex, ITN use, IRS in the past 12 months, household wealth, education level of the household head | Child's age, sex, birthweight, healthcard ownership, receipt of vitamin A, DPT-3 vaccination, measles-1 vaccination, household wealth, education level of the household head | Child's age, sex, birthweight, healthcard ownership, receipt of vitamin A, DPT-3 vaccination, measles-1 vaccination, main household cooking fuel, household wealth, education level of the household head | Child's age, sex, birthweight, healthcard ownership, DPT-3 vaccination, measles-1 vaccination, ITN use, household wealth, education level of the household head | Child's age, sex, birthweight, healthcard ownership, DPT-3 vaccination, measles-1 vaccination, ITN use, household wealth, education level of the household head | Child's age, sex, birthweight, healthcard ownership, DPT-3 vaccination, measles-1 vaccination, ITN use, household wealth, education level of the household head | Child's age, sex, birthweight, ITN use, healthcard ownership, receipt of vitamin A, DPT-3 vaccination, measles-1 vaccination, household wealth, education level of the household head |

**Table B.** Surveys included (n=74) and covariables adjusted for in the analysis of house type

| **Survey** | **Malaria infection (microscopy)** | **Malaria infection (RDT)** | **Diarrhoea** | **Acute respiratory infection** | **Low height-for-age** | **Low weight-for-height** | **Low weight-for-age** | **Anaemia** |
|---|---|---|---|---|---|---|---|---|
| Comoros 2012 DHS | - | - | Child's age, sex, birthweight, healthcard ownership, receipt of vitamin A, DPT-3 vaccination, measles-1 vaccination, household wealth, education level of the household head | Child's age, sex, birthweight, healthcard ownership, receipt of vitamin A, DPT-3 vaccination, measles-1 vaccination, smoking in house, main household cooking fuel, household wealth, education level of the household head | Child's age, sex, birthweight, healthcard ownership, DPT-3 vaccination, measles-1 vaccination, ITN use, household wealth, education level of the household head | Child's age, sex, birthweight, healthcard ownership, DPT-3 vaccination, measles-1 vaccination, ITN use, household wealth, education level of the household head | Child's age, sex, birthweight, healthcard ownership, DPT-3 vaccination, measles-1 vaccination, ITN use, household wealth, education level of the household head | - |
| Congo 2005 DHS | - | - | Child's age, sex, birthweight, healthcard ownership, receipt of vitamin A, DPT-3 vaccination, measles-1 vaccination, household wealth, education level of the household head | Child's age, sex, birthweight, healthcard ownership, receipt of vitamin A, DPT-3 vaccination, measles-1 vaccination, main household cooking fuel, household wealth, education level of the household head | Child's age, sex, birthweight, healthcard ownership, DPT-3 vaccination, measles-1 vaccination, ITN use, household wealth, education level of the household head | Child's age, sex, birthweight, healthcard ownership, DPT-3 vaccination, measles-1 vaccination, ITN use, household wealth, education level of the household head | Child's age, sex, birthweight, healthcard ownership, DPT-3 vaccination, measles-1 vaccination, ITN use, household wealth, education level of the household head | Child's age, sex, birthweight, ITN use, healthcard ownership, receipt of vitamin A, DPT-3 vaccination, measles-1 vaccination, household wealth, education level of the household head |

**Table B.** Surveys included (n=74) and covariables adjusted for in the analysis of house type

| Survey | Malaria infection (microscopy) | Malaria infection (RDT) | Diarrhoea | Acute respiratory infection | Low height-for-age | Low weight-for-height | Low weight-for-age | Anaemia |
|---|---|---|---|---|---|---|---|---|
| Congo 2011 DHS | - | - | Child's age, sex, birthweight, healthcard ownership, receipt of vitamin A, DPT-3 vaccination, measles-1 vaccination, household wealth, education level of the household head | Child's age, sex, birthweight, healthcard ownership, receipt of vitamin A, DPT-3 vaccination, measles-1 vaccination, smoking in house, main household cooking fuel, household wealth, education level of the household head | Child's age, sex, birthweight, healthcard ownership, DPT-3 vaccination, measles-1 vaccination, ITN use, household wealth, education level of the household head | Child's age, sex, birthweight, healthcard ownership, DPT-3 vaccination, measles-1 vaccination, ITN use, household wealth, education level of the household head | Child's age, sex, birthweight, healthcard ownership, DPT-3 vaccination, measles-1 vaccination, ITN use, household wealth, education level of the household head | Child's age, sex, birthweight, ITN use, healthcard ownership, receipt of vitamin A, DPT-3 vaccination, measles-1 vaccination, household wealth, education level of the household head |
| Cote d'Ivoire 2012 DHS | Child's age, sex, ITN use, IRS in the past 12 months, household wealth, education level of the household head | Child's age, sex, ITN use, IRS in the past 12 months, household wealth, education level of the household head | Child's age, sex, birthweight, healthcard ownership, receipt of vitamin A, DPT-3 vaccination, measles-1 vaccination, household wealth, education level of the household head | Child's age, sex, birthweight, healthcard ownership, receipt of vitamin A, DPT-3 vaccination, measles-1 vaccination, smoking in house, main household cooking fuel, household wealth, education level of the household head | Child's age, sex, birthweight, healthcard ownership, DPT-3 vaccination, measles-1 vaccination, ITN use, household wealth, education level of the household head | Child's age, sex, birthweight, healthcard ownership, DPT-3 vaccination, measles-1 vaccination, ITN use, household wealth, education level of the household head | Child's age, sex, birthweight, healthcard ownership, DPT-3 vaccination, measles-1 vaccination, ITN use, household wealth, education level of the household head | Child's age, sex, birthweight, ITN use, healthcard ownership, receipt of vitamin A, DPT-3 vaccination, measles-1 vaccination, household wealth, education level of the household head |

**Table B.** Surveys included (n=74) and covariables adjusted for in the analysis of house type

| Survey | Malaria infection (microscopy) | Malaria infection (RDT) | Diarrhoea | Acute respiratory infection | Low height-for-age | Low weight-for-height | Low weight-for-age | Anaemia |
|---|---|---|---|---|---|---|---|---|
| DRC 2013 DHS | Child's age, sex, ITN use, household wealth, education level of the household head | Child's age, sex, ITN use, household wealth, education level of the household head | Child's age, sex, birthweight, healthcard ownership, receipt of vitamin A, DPT-3 vaccination, measles-1 vaccination, household wealth, education level of the household head | Child's age, sex, birthweight, healthcard ownership, receipt of vitamin A, DPT-3 vaccination, measles-1 vaccination, smoking in house, main household cooking fuel, household wealth, education level of the household head | Child's age, sex, birthweight, healthcard ownership, DPT-3 vaccination, measles-1 vaccination, ITN use, household wealth, education level of the household head | Child's age, sex, birthweight, healthcard ownership, DPT-3 vaccination, measles-1 vaccination, ITN use, household wealth, education level of the household head | Child's age, sex, birthweight, healthcard ownership, DPT-3 vaccination, measles-1 vaccination, ITN use, household wealth, education level of the household head | Child's age, sex, birthweight, ITN use, healthcard ownership, receipt of vitamin A, DPT-3 vaccination, measles-1 vaccination, household wealth, education level of the household head |
| Eswatini 2006 DHS | - | - | Child's age, sex, birthweight, healthcard ownership, receipt of vitamin A, DPT-3 vaccination, measles-1 vaccination, household wealth, education level of the household head | Child's age, sex, birthweight, healthcard ownership, receipt of vitamin A, DPT-3 vaccination, measles-1 vaccination, main household cooking fuel, household wealth, education level of the household head | Child's age, sex, birthweight, healthcard ownership, DPT-3 vaccination, measles-1 vaccination, ITN use, household wealth, education level of the household head | Child's age, sex, birthweight, healthcard ownership, DPT-3 vaccination, measles-1 vaccination, ITN use, household wealth, education level of the household head | Child's age, sex, birthweight, healthcard ownership, DPT-3 vaccination, measles-1 vaccination, ITN use, household wealth, education level of the household head | Child's age, sex, birthweight, ITN use, healthcard ownership, receipt of vitamin A, DPT-3 vaccination, measles-1 vaccination, household wealth, education level of the household head |

**Table B.** Surveys included (n=74) and covariables adjusted for in the analysis of house type

| Survey | Malaria infection (microscopy) | Malaria infection (RDT) | Diarrhoea | Acute respiratory infection | Low height-for-age | Low weight-for-height | Low weight-for-age | Anaemia |
|---|---|---|---|---|---|---|---|---|
| Ethiopia 2016 DHS | - | - | Child's age, sex, birthweight, healthcard ownership, receipt of vitamin A, DPT-3 vaccination, measles-1 vaccination, rotavirus-2 vaccination, household wealth, education level of the household head | Child's age, sex, birthweight, healthcard ownership, receipt of vitamin A, DPT-3 vaccination, measles-1 vaccination, pneumococcal-3 vaccination, smoking in house, main household cooking fuel, household wealth, education level of the household head | - | - | - | - |
| Gabon 2012 DHS | - | - | Child's age, sex, birthweight, healthcard ownership, receipt of vitamin A, DPT-3 vaccination, measles-1 vaccination, household wealth, education level of the household head | Child's age, sex, birthweight, healthcard ownership, receipt of vitamin A, DPT-3 vaccination, measles-1 vaccination, smoking in house, main household cooking fuel, household wealth, education level of the household head | Child's age, sex, birthweight, healthcard ownership, DPT-3 vaccination, measles-1 vaccination, ITN use, household wealth, education level of the household head | Child's age, sex, birthweight, healthcard ownership, DPT-3 vaccination, measles-1 vaccination, ITN use, household wealth, education level of the household head | Child's age, sex, birthweight, healthcard ownership, DPT-3 vaccination, measles-1 vaccination, ITN use, household wealth, education level of the household head | Child's age, sex, birthweight, ITN use, healthcard ownership, receipt of vitamin A, DPT-3 vaccination, measles-1 vaccination, household wealth, education level of the household head |

**Table B.** Surveys included (n=74) and covariables adjusted for in the analysis of house type

| Survey | Malaria infection (microscopy) | Malaria infection (RDT) | Diarrhoea | Acute respiratory infection | Low height-for-age | Low weight-for-height | Low weight-for-age | Anaemia |
|---|---|---|---|---|---|---|---|---|
| Gambia 2013 DHS | - | Child's age, sex, ITN use, IRS in the past 12 months, household wealth, education level of the household head | Child's age, sex, birthweight, healthcard ownership, receipt of vitamin A, DPT-3 vaccination, measles-1 vaccination, household wealth, education level of the household head | - | Child's age, sex, birthweight, healthcard ownership, DPT-3 vaccination, measles-1 vaccination, ITN use, household wealth, education level of the household head | Child's age, sex, birthweight, healthcard ownership, DPT-3 vaccination, measles-1 vaccination, ITN use, household wealth, education level of the household head | Child's age, sex, birthweight, healthcard ownership, DPT-3 vaccination, measles-1 vaccination, ITN use, household wealth, education level of the household head | Child's age, sex, birthweight, ITN use, healthcard ownership, receipt of vitamin A, DPT-3 vaccination, measles-1 vaccination, household wealth, education level of the household head |
| Ghana 2008 DHS | - | - | Child's age, sex, birthweight, healthcard ownership, receipt of vitamin A, DPT-3 vaccination, measles-1 vaccination, household wealth, education level of the household head | - | Child's age, sex, birthweight, healthcard ownership, DPT-3 vaccination, measles-1 vaccination, ITN use, household wealth, education level of the household head | Child's age, sex, birthweight, healthcard ownership, DPT-3 vaccination, measles-1 vaccination, ITN use, household wealth, education level of the household head | Child's age, sex, birthweight, healthcard ownership, DPT-3 vaccination, measles-1 vaccination, ITN use, household wealth, education level of the household head | Child's age, sex, birthweight, ITN use, healthcard ownership, receipt of vitamin A, DPT-3 vaccination, measles-1 vaccination, household wealth, education level of the household head |
| Ghana 2014 DHS | Child's age, sex, ITN use, IRS in the past 12 months, household wealth, education level of the household head | Child's age, sex, ITN use, IRS in the past 12 months, household wealth, education level of the household head | Child's age, sex, birthweight, healthcard ownership, receipt of vitamin A, DPT-3 vaccination, measles-1 vaccination, household wealth, education level of the household head | Child's age, sex, birthweight, healthcard ownership, receipt of vitamin A, DPT-3 vaccination, measles-1 vaccination, smoking in house, main household cooking fuel, household wealth, education level of the household head | Child's age, sex, birthweight, healthcard ownership, DPT-3 vaccination, measles-1 vaccination, ITN use, household wealth, education level of the household head | Child's age, sex, birthweight, healthcard ownership, DPT-3 vaccination, measles-1 vaccination, ITN use, household wealth, education level of the household head | Child's age, sex, birthweight, healthcard ownership, DPT-3 vaccination, measles-1 vaccination, ITN use, household wealth, education level of the household head | Child's age, sex, birthweight, ITN use, healthcard ownership, receipt of vitamin A, DPT-3 vaccination, measles-1 vaccination, household wealth, education level of the household head |

**Table B.** Surveys included (n=74) and covariables adjusted for in the analysis of house type

| Survey | Malaria infection (microscopy) | Malaria infection (RDT) | Diarrhoea | Acute respiratory infection | Low height-for-age | Low weight-for-height | Low weight-for-age | Anaemia |
|---|---|---|---|---|---|---|---|---|
| Ghana 2016 MIS | Child's age, sex, ITN use, IRS in the past 12 months, household wealth | Child's age, sex, ITN use, IRS in the past 12 months, household wealth | - | - | - | - | - | - |
| Guinea 2012 DHS | Child's age, sex, ITN use, IRS in the past 12 months, household wealth, education level of the household head | Child's age, sex, ITN use, IRS in the past 12 months, household wealth, education level of the household head | Child's age, sex, birthweight, healthcard ownership, receipt of vitamin A, DPT-3 vaccination, measles-1 vaccination, household wealth, education level of the household head | Child's age, sex, birthweight, healthcard ownership, receipt of vitamin A, DPT-3 vaccination, measles-1 vaccination, smoking in house, main household cooking fuel, household wealth, education level of the household head | Child's age, sex, birthweight, healthcard ownership, DPT-3 vaccination, measles-1 vaccination, ITN use, household wealth, education level of the household head | Child's age, sex, birthweight, healthcard ownership, DPT-3 vaccination, measles-1 vaccination, ITN use, household wealth, education level of the household head | Child's age, sex, birthweight, healthcard ownership, DPT-3 vaccination, measles-1 vaccination, ITN use, household wealth, education level of the household head | Child's age, sex, birthweight, ITN use, healthcard ownership, receipt of vitamin A, DPT-3 vaccination, measles-1 vaccination, household wealth, education level of the household head |
| Kenya 2008 DHS | - | - | Child's age, sex, birthweight, healthcard ownership, receipt of vitamin A, DPT-3 vaccination, measles-1 vaccination, household wealth, education level of the household head | Child's age, sex, birthweight, healthcard ownership, receipt of vitamin A, DPT-3 vaccination, measles-1 vaccination, main household cooking fuel, household wealth, education level of the household head | Child's age, sex, birthweight, healthcard ownership, DPT-3 vaccination, measles-1 vaccination, ITN use, household wealth, education level of the household head | Child's age, sex, birthweight, healthcard ownership, DPT-3 vaccination, measles-1 vaccination, ITN use, household wealth, education level of the household head | Child's age, sex, birthweight, healthcard ownership, DPT-3 vaccination, measles-1 vaccination, ITN use, household wealth, education level of the household head | - |

**Table B.** Surveys included (n=74) and covariables adjusted for in the analysis of house type

| Survey | Malaria infection (microscopy) | Malaria infection (RDT) | Diarrhoea | Acute respiratory infection | Low height-for-age | Low weight-for-height | Low weight-for-age | Anaemia |
|---|---|---|---|---|---|---|---|---|
| Kenya 2014 DHS | - | - | Child's age, sex, birthweight, healthcard ownership, receipt of vitamin A, DPT-3 vaccination, measles-1 vaccination, household wealth, education level of the household head | Child's age, sex, birthweight, healthcard ownership, receipt of vitamin A, DPT-3 vaccination, measles-1 vaccination, smoking in house, main household cooking fuel, household wealth, education level of the household head | Child's age, sex, birthweight, healthcard ownership, DPT-3 vaccination, measles-1 vaccination, ITN use, household wealth, education level of the household head | Child's age, sex, birthweight, healthcard ownership, DPT-3 vaccination, measles-1 vaccination, ITN use, household wealth, education level of the household head | Child's age, sex, birthweight, healthcard ownership, DPT-3 vaccination, measles-1 vaccination, ITN use, household wealth, education level of the household head | - |
| Kenya 2015 MIS | - | Child's age, sex, ITN use, household wealth | - | - | - | - | - | - |
| Lesotho 2009 DHS | - | - | Child's age, sex, birthweight, healthcard ownership, receipt of vitamin A, DPT-3 vaccination, measles-1 vaccination, household wealth, education level of the household head | - | - | - | - | - |

**Table B.** Surveys included (n=74) and covariables adjusted for in the analysis of house type

| Survey | Malaria infection (microscopy) | Malaria infection (RDT) | Diarrhoea | Acute respiratory infection | Low height-for-age | Low weight-for-height | Low weight-for-age | Anaemia |
|---|---|---|---|---|---|---|---|---|
| Lesotho 2014 DHS | - | - | Child's age, sex, birthweight, healthcard ownership, receipt of vitamin A, DPT-3 vaccination, measles-1 vaccination, household wealth, education level of the household head | Child's age, sex, birthweight, healthcard ownership, receipt of vitamin A, DPT-3 vaccination, measles-1 vaccination, smoking in house, main household cooking fuel, household wealth, education level of the household head | - | - | - | - |
| Liberia 2011 MIS | Child's age, sex, ITN use, IRS in the past 12 months, household wealth | Child's age, sex, ITN use, IRS in the past 12 months, household wealth | - | - | - | - | - | - |
| Liberia 2013 DHS | - | - | Child's age, sex, birthweight, healthcard ownership, receipt of vitamin A, DPT-3 vaccination, measles-1 vaccination, household wealth, education level of the household head | Child's age, sex, birthweight, healthcard ownership, receipt of vitamin A, DPT-3 vaccination, measles-1 vaccination, smoking in house, main household cooking fuel, household wealth, education level of the household head | Child's age, sex, birthweight, healthcard ownership, DPT-3 vaccination, measles-1 vaccination, ITN use, household wealth, education level of the household head | Child's age, sex, birthweight, healthcard ownership, DPT-3 vaccination, measles-1 vaccination, ITN use, household wealth, education level of the household head | Child's age, sex, birthweight, healthcard ownership, DPT-3 vaccination, measles-1 vaccination, ITN use, household wealth, education level of the household head | - |
| Liberia 2016 MIS | - | Child's age, sex, ITN use, IRS in the past 12 months, household wealth | - | - | - | - | - | - |

**Table B.** Surveys included (n=74) and covariables adjusted for in the analysis of house type

| Survey | Malaria infection (microscopy) | Malaria infection (RDT) | Diarrhoea | Acute respiratory infection | Low height-for-age | Low weight-for-height | Low weight-for-age | Anaemia |
|---|---|---|---|---|---|---|---|---|
| Madagascar 2008 DHS | - | - | Child's age, sex, birthweight, healthcard ownership, receipt of vitamin A, DPT-3 vaccination, measles-1 vaccination, household wealth, education level of the household head | - | Child's age, sex, birthweight, healthcard ownership, DPT-3 vaccination, measles-1 vaccination, ITN use, household wealth, education level of the household head | - | - | Child's age, sex, birthweight, ITN use, healthcard ownership, receipt of vitamin A, DPT-3 vaccination, measles-1 vaccination, household wealth, education level of the household head |
| Madagascar 2013 MIS | Child's age, sex, ITN use, IRS in the past 12 months, household wealth | Child's age, sex, ITN use, IRS in the past 12 months, household wealth | - | - | - | - | - | - |
| Malawi 2010 DHS | - | - | Child's age, sex, birthweight, healthcard ownership, receipt of vitamin A, DPT-3 vaccination, measles-1 vaccination, household wealth, education level of the household head | Child's age, sex, birthweight, healthcard ownership, receipt of vitamin A, DPT-3 vaccination, measles-1 vaccination, main household cooking fuel, household wealth, education level of the household head | Child's age, sex, birthweight, healthcard ownership, DPT-3 vaccination, measles-1 vaccination, ITN use, household wealth, education level of the household head | Child's age, sex, birthweight, healthcard ownership, DPT-3 vaccination, measles-1 vaccination, ITN use, household wealth, education level of the household head | Child's age, sex, birthweight, healthcard ownership, DPT-3 vaccination, measles-1 vaccination, ITN use, household wealth, education level of the household head | Child's age, sex, birthweight, ITN use, healthcard ownership, receipt of vitamin A, DPT-3 vaccination, measles-1 vaccination, household wealth, education level of the household head |
| Malawi 2012 MIS | Child's age, sex, ITN use, IRS in the past 12 months, household wealth | Child's age, sex, ITN use, IRS in the past 12 months, household wealth | - | - | - | - | - | - |
| Malawi 2014 MIS | Child's age, sex, ITN use, IRS in the past 12 months, household wealth | Child's age, sex, ITN use, IRS in the past 12 months, household wealth | - | - | - | - | - | - |

**Table B.** Surveys included (n=74) and covariables adjusted for in the analysis of house type

| Survey | Malaria infection (microscopy) | Malaria infection (RDT) | Diarrhoea | Acute respiratory infection | Low height-for-age | Low weight-for-height | Low weight-for-age | Anaemia |
|---|---|---|---|---|---|---|---|---|
| Malawi 2015 DHS | - | - | Child's age, sex, birthweight, healthcard ownership, receipt of vitamin A, DPT-3 vaccination, measles-1 vaccination, rotavirus-2 vaccination, household wealth, education level of the household head | Child's age, sex, birthweight, healthcard ownership, receipt of vitamin A, DPT-3 vaccination, measles-1 vaccination, pneumococcal-3 vaccination, smoking in house, main household cooking fuel, household wealth, education level of the household head | Child's age, sex, birthweight, healthcard ownership, DPT-3 vaccination, measles-1 vaccination, ITN use, household wealth, education level of the household head | Child's age, sex, birthweight, healthcard ownership, DPT-3 vaccination, measles-1 vaccination, ITN use, household wealth, education level of the household head | Child's age, sex, birthweight, healthcard ownership, DPT-3 vaccination, measles-1 vaccination, ITN use, household wealth, education level of the household head | Child's age, sex, birthweight, ITN use, healthcard ownership, receipt of vitamin A, DPT-3 vaccination, measles-1 vaccination, rotavirus-2 vaccination, pneumococcal-3 vaccination, household wealth, education level of the household head |
| Malawi 2017 MIS | Child's age, sex, ITN use, household wealth | Child's age, sex, ITN use, household wealth | - | - | - | - | - | - |
| Mali 2012 DHS | Child's age, sex, ITN use, IRS in the past 12 months, household wealth, education level of the household head | Child's age, sex, ITN use, IRS in the past 12 months, household wealth, education level of the household head | Child's age, sex, birthweight, healthcard ownership, receipt of vitamin A, DPT-3 vaccination, measles-1 vaccination, household wealth, education level of the household head | - | Child's age, sex, birthweight, healthcard ownership, DPT-3 vaccination, measles-1 vaccination, ITN use, household wealth, education level of the household head | Child's age, sex, birthweight, healthcard ownership, DPT-3 vaccination, measles-1 vaccination, ITN use, household wealth, education level of the household head | Child's age, sex, birthweight, healthcard ownership, DPT-3 vaccination, measles-1 vaccination, ITN use, household wealth, education level of the household head | Child's age, sex, birthweight, ITN use, healthcard ownership, receipt of vitamin A, DPT-3 vaccination, measles-1 vaccination, household wealth, education level of the household head |
| Mali 2015 MIS | Child's age, sex, ITN use, IRS in the past 12 months, household wealth | Child's age, sex, ITN use, IRS in the past 12 months, household wealth | - | - | - | - | - | - |

**Table B.** Surveys included (n=74) and covariables adjusted for in the analysis of house type

| Survey | Malaria infection (microscopy) | Malaria infection (RDT) | Diarrhoea | Acute respiratory infection | Low height-for-age | Low weight-for-height | Low weight-for-age | Anaemia |
|---|---|---|---|---|---|---|---|---|
| Mozambique 2011 DHS | Child's age, sex, ITN use, IRS in the past 12 months, household wealth, education level of the household head | Child's age, sex, ITN use, IRS in the past 12 months, household wealth, education level of the household head | Child's age, sex, birthweight, healthcard ownership, receipt of vitamin A, DPT-3 vaccination, measles-1 vaccination, household wealth, education level of the household head | - | Child's age, sex, birthweight, healthcard ownership, DPT-3 vaccination, measles-1 vaccination, ITN use, household wealth, education level of the household head | Child's age, sex, birthweight, healthcard ownership, DPT-3 vaccination, measles-1 vaccination, ITN use, household wealth, education level of the household head | Child's age, sex, birthweight, healthcard ownership, DPT-3 vaccination, measles-1 vaccination, ITN use, household wealth, education level of the household head | Child's age, sex, birthweight, ITN use, healthcard ownership, receipt of vitamin A, DPT-3 vaccination, measles-1 vaccination, household wealth, education level of the household head |
| Mozambique 2015 AIS | - | Child's age, sex, ITN use, IRS in the past 12 months, household wealth, education level of the household head | - | - | - | - | - | - |
| Namibia 2006 DHS | - | - | Child's age, sex, birthweight, healthcard ownership, receipt of vitamin A, DPT-3 vaccination, measles-1 vaccination, household wealth, education level of the household head | Child's age, sex, birthweight, healthcard ownership, receipt of vitamin A, DPT-3 vaccination, measles-1 vaccination, main household cooking fuel, household wealth, education level of the household head | Child's age, sex, birthweight, healthcard ownership, DPT-3 vaccination, measles-1 vaccination, ITN use, household wealth, education level of the household head | Child's age, sex, birthweight, healthcard ownership, DPT-3 vaccination, measles-1 vaccination, ITN use, household wealth, education level of the household head | Child's age, sex, birthweight, healthcard ownership, DPT-3 vaccination, measles-1 vaccination, ITN use, household wealth, education level of the household head | - |

**Table B.** Surveys included (n=74) and covariables adjusted for in the analysis of house type

| Survey | Malaria infection (microscopy) | Malaria infection (RDT) | Diarrhoea | Acute respiratory infection | Low height-for-age | Low weight-for-height | Low weight-for-age | Anaemia |
|---|---|---|---|---|---|---|---|---|
| Namibia 2013 DHS | - | - | Child's age, sex, birthweight, healthcard ownership, receipt of vitamin A, DPT-3 vaccination, measles-1 vaccination, household wealth, education level of the household head | Child's age, sex, birthweight, healthcard ownership, receipt of vitamin A, DPT-3 vaccination, measles-1 vaccination, smoking in house, main household cooking fuel, household wealth, education level of the household head | Child's age, sex, birthweight, healthcard ownership, DPT-3 vaccination, measles-1 vaccination, ITN use, household wealth, education level of the household head | Child's age, sex, birthweight, healthcard ownership, DPT-3 vaccination, measles-1 vaccination, ITN use, household wealth, education level of the household head | Child's age, sex, birthweight, healthcard ownership, DPT-3 vaccination, measles-1 vaccination, ITN use, household wealth, education level of the household head | Child's age, sex, birthweight, ITN use, healthcard ownership, receipt of vitamin A, DPT-3 vaccination, measles-1 vaccination, household wealth, education level of the household head |
| Niger 2012 DHS | - | - | Child's age, sex, birthweight, healthcard ownership, receipt of vitamin A, DPT-3 vaccination, measles-1 vaccination, household wealth, education level of the household head | Child's age, sex, birthweight, healthcard ownership, receipt of vitamin A, DPT-3 vaccination, measles-1 vaccination, main household cooking fuel, household wealth, education level of the household head | Child's age, sex, birthweight, healthcard ownership, DPT-3 vaccination, measles-1 vaccination, ITN use, household wealth, education level of the household head | Child's age, sex, birthweight, healthcard ownership, DPT-3 vaccination, measles-1 vaccination, ITN use, household wealth, education level of the household head | Child's age, sex, birthweight, healthcard ownership, DPT-3 vaccination, measles-1 vaccination, ITN use, household wealth, education level of the household head | Child's age, sex, birthweight, ITN use, healthcard ownership, receipt of vitamin A, DPT-3 vaccination, measles-1 vaccination, household wealth, education level of the household head |
| Nigeria 2008 DHS | - | - | Child's age, sex, birthweight, healthcard ownership, receipt of vitamin A, DPT-3 vaccination, measles-1 vaccination, household wealth, education level of the household head | - | Child's age, sex, birthweight, healthcard ownership, DPT-3 vaccination, measles-1 vaccination, ITN use, household wealth, education level of the household head | Child's age, sex, birthweight, healthcard ownership, DPT-3 vaccination, measles-1 vaccination, ITN use, household wealth, education level of the household head | Child's age, sex, birthweight, healthcard ownership, DPT-3 vaccination, measles-1 vaccination, ITN use, household wealth, education level of the household head | - |

**Table B.** Surveys included (n=74) and covariables adjusted for in the analysis of house type

| Survey | Malaria infection (microscopy) | Malaria infection (RDT) | Diarrhoea | Acute respiratory infection | Low height-for-age | Low weight-for-height | Low weight-for-age | Anaemia |
|---|---|---|---|---|---|---|---|---|
| Nigeria 2010 MIS | Child's age, sex, ITN use, IRS in the past 12 months, household wealth, education level of the household head | Child's age, sex, ITN use, IRS in the past 12 months, household wealth, education level of the household head | - | - | - | - | - | - |
| Nigeria 2013 DHS | - | - | Child's age, sex, birthweight, healthcard ownership, receipt of vitamin A, DPT-3 vaccination, measles-1 vaccination, household wealth, education level of the household head | - | Child's age, sex, birthweight, healthcard ownership, DPT-3 vaccination, measles-1 vaccination, ITN use, household wealth, education level of the household head | Child's age, sex, birthweight, healthcard ownership, DPT-3 vaccination, measles-1 vaccination, ITN use, household wealth, education level of the household head | Child's age, sex, birthweight, healthcard ownership, DPT-3 vaccination, measles-1 vaccination, ITN use, household wealth, education level of the household head | - |
| Nigeria 2015 MIS | Child's age, sex, ITN use, IRS in the past 12 months, household wealth, education level of the household head | Child's age, sex, ITN use, IRS in the past 12 months, household wealth, education level of the household head | - | - | - | - | - | - |
| Rwanda 2010 DHS | Child's age, sex, ITN use, household wealth, education level of the household head | Child's age, sex, ITN use, household wealth, education level of the household head | Child's age, sex, birthweight, healthcard ownership, receipt of vitamin A, DPT-3 vaccination, measles-1 vaccination, household wealth, education level of the household head | Child's age, sex, birthweight, healthcard ownership, receipt of vitamin A, DPT-3 vaccination, measles-1 vaccination, smoking in house, main household cooking fuel, household wealth, education level of the household head | Child's age, sex, birthweight, healthcard ownership, DPT-3 vaccination, measles-1 vaccination, ITN use, household wealth, education level of the household head | Child's age, sex, birthweight, healthcard ownership, DPT-3 vaccination, measles-1 vaccination, ITN use, household wealth, education level of the household head | Child's age, sex, birthweight, healthcard ownership, DPT-3 vaccination, measles-1 vaccination, ITN use, household wealth, education level of the household head | Child's age, sex, birthweight, ITN use, healthcard ownership, receipt of vitamin A, DPT-3 vaccination, measles-1 vaccination, household wealth, education level of the household head |

**Table B.** Surveys included (n=74) and covariables adjusted for in the analysis of house type

| Survey | Malaria infection (microscopy) | Malaria infection (RDT) | Diarrhoea | Acute respiratory infection | Low height-for-age | Low weight-for-height | Low weight-for-age | Anaemia |
|---|---|---|---|---|---|---|---|---|
| Rwanda 2015 DHS | Child's age, sex, ITN use, household wealth, education level of the household head | Child's age, sex, ITN use, household wealth, education level of the household head | Child's age, sex, birthweight, healthcard ownership, receipt of vitamin A, DPT-3 vaccination, measles-1 vaccination, rotavirus-2 vaccination, household wealth, education level of the household head | Child's age, sex, birthweight, healthcard ownership, receipt of vitamin A, DPT-3 vaccination, measles-1 vaccination, smoking in house, main household cooking fuel, household wealth, education level of the household head | Child's age, sex, birthweight, healthcard ownership, DPT-3 vaccination, measles-1 vaccination, ITN use, household wealth, education level of the household head | Child's age, sex, birthweight, healthcard ownership, DPT-3 vaccination, measles-1 vaccination, ITN use, household wealth, education level of the household head | Child's age, sex, birthweight, healthcard ownership, DPT-3 vaccination, measles-1 vaccination, ITN use, household wealth, education level of the household head | Child's age, sex, birthweight, ITN use, healthcard ownership, receipt of vitamin A, DPT-3 vaccination, measles-1 vaccination, rotavirus-2 vaccination, household wealth, education level of the household head |
| Rwanda 2017 MIS | Child's age, sex, ITN use, IRS in the past 12 months, household wealth | Child's age, sex, ITN use, IRS in the past 12 months, household wealth | - | - | - | - | - | - |
| Senegal 2008 MIS | Child's age, sex, ITN use, household wealth | Child's age, sex, ITN use, household wealth | - | - | - | - | - | - |
| Senegal 2010 DHS | Child's age, sex, ITN use, IRS in the past 12 months, household wealth, education level of the household head | Child's age, sex, ITN use, IRS in the past 12 months, household wealth, education level of the household head | Child's age, sex, birthweight, healthcard ownership, receipt of vitamin A, DPT-3 vaccination, measles-1 vaccination, household wealth, education level of the household head | Child's age, sex, birthweight, healthcard ownership, receipt of vitamin A, DPT-3 vaccination, measles-1 vaccination, main household cooking fuel, household wealth, education level of the household head | Child's age, sex, birthweight, healthcard ownership, DPT-3 vaccination, measles-1 vaccination, ITN use, household wealth, education level of the household head | Child's age, sex, birthweight, healthcard ownership, DPT-3 vaccination, measles-1 vaccination, ITN use, household wealth, education level of the household head | Child's age, sex, birthweight, healthcard ownership, DPT-3 vaccination, measles-1 vaccination, ITN use, household wealth, education level of the household head | Child's age, sex, birthweight, ITN use, healthcard ownership, receipt of vitamin A, DPT-3 vaccination, measles-1 vaccination, household wealth, education level of the household head |

**Table B.** Surveys included (n=74) and covariables adjusted for in the analysis of house type

| Survey | Malaria infection (microscopy) | Malaria infection (RDT) | Diarrhoea | Acute respiratory infection | Low height-for-age | Low weight-for-height | Low weight-for-age | Anaemia |
|---|---|---|---|---|---|---|---|---|
| Senegal 2012 DHS | Child's age, sex, ITN use, IRS in the past 12 months, household wealth, education level of the household head | Child's age, sex, ITN use, IRS in the past 12 months, household wealth, education level of the household head | Child's age, sex, birthweight, healthcard ownership, receipt of vitamin A, DPT-3 vaccination, measles-1 vaccination, household wealth, education level of the household head | - | Child's age, sex, birthweight, healthcard ownership, DPT-3 vaccination, measles-1 vaccination, ITN use, household wealth, education level of the household head | Child's age, sex, birthweight, healthcard ownership, DPT-3 vaccination, measles-1 vaccination, ITN use, household wealth, education level of the household head | Child's age, sex, birthweight, healthcard ownership, DPT-3 vaccination, measles-1 vaccination, ITN use, household wealth, education level of the household head | Child's age, sex, birthweight, ITN use, healthcard ownership, receipt of vitamin A, DPT-3 vaccination, measles-1 vaccination, household wealth, education level of the household head |
| Senegal 2014 DHS | Child's age, sex, ITN use, IRS in the past 12 months, household wealth, education level of the household head | Child's age, sex, ITN use, IRS in the past 12 months, household wealth, education level of the household head | Child's age, sex, birthweight, healthcard ownership, receipt of vitamin A, DPT-3 vaccination, measles-1 vaccination, household wealth, education level of the household head | - | Child's age, sex, birthweight, healthcard ownership, DPT-3 vaccination, measles-1 vaccination, ITN use, household wealth, education level of the household head | Child's age, sex, birthweight, healthcard ownership, DPT-3 vaccination, measles-1 vaccination, ITN use, household wealth, education level of the household head | Child's age, sex, birthweight, healthcard ownership, DPT-3 vaccination, measles-1 vaccination, ITN use, household wealth, education level of the household head | Child's age, sex, birthweight, ITN use, healthcard ownership, receipt of vitamin A, DPT-3 vaccination, measles-1 vaccination, household wealth, education level of the household head |
| Senegal 2015 DHS | Child's age, sex, ITN use, IRS in the past 12 months, household wealth, education level of the household head | Child's age, sex, ITN use, IRS in the past 12 months, household wealth, education level of the household head | Child's age, sex, birthweight, healthcard ownership, receipt of vitamin A, DPT-3 vaccination, measles-1 vaccination, household wealth, education level of the household head | - | Child's age, sex, birthweight, healthcard ownership, DPT-3 vaccination, measles-1 vaccination, ITN use, household wealth, education level of the household head | Child's age, sex, birthweight, healthcard ownership, DPT-3 vaccination, measles-1 vaccination, ITN use, household wealth, education level of the household head | Child's age, sex, birthweight, healthcard ownership, DPT-3 vaccination, measles-1 vaccination, ITN use, household wealth, education level of the household head | Child's age, sex, birthweight, ITN use, healthcard ownership, receipt of vitamin A, DPT-3 vaccination, measles-1 vaccination, household wealth, education level of the household head |

**Table B.** Surveys included (n=74) and covariables adjusted for in the analysis of house type

| Survey | Malaria infection (microscopy) | Malaria infection (RDT) | Diarrhoea | Acute respiratory infection | Low height-for-age | Low weight-for-height | Low weight-for-age | Anaemia |
|---|---|---|---|---|---|---|---|---|
| Senegal 2016 DHS | Child's age, sex, ITN use, IRS in the past 12 months, household wealth, education level of the household head | Child's age, sex, ITN use, IRS in the past 12 months, household wealth, education level of the household head | Child's age, sex, birthweight, healthcard ownership, receipt of vitamin A, DPT-3 vaccination, measles-1 vaccination, household wealth, education level of the household head | - | Child's age, sex, birthweight, healthcard ownership, DPT-3 vaccination, measles-1 vaccination, ITN use, household wealth, education level of the household head | Child's age, sex, birthweight, healthcard ownership, DPT-3 vaccination, measles-1 vaccination, ITN use, household wealth, education level of the household head | Child's age, sex, birthweight, healthcard ownership, DPT-3 vaccination, measles-1 vaccination, ITN use, household wealth, education level of the household head | Child's age, sex, birthweight, ITN use, healthcard ownership, receipt of vitamin A, DPT-3 vaccination, measles-1 vaccination, household wealth, education level of the household head |
| Sierra Leone 2008 DHS | - | - | Child's age, sex, birthweight, healthcard ownership, receipt of vitamin A, DPT-3 vaccination, measles-1 vaccination, household wealth, education level of the household head | - | Child's age, sex, birthweight, healthcard ownership, DPT-3 vaccination, measles-1 vaccination, ITN use, household wealth, education level of the household head | Child's age, sex, birthweight, healthcard ownership, DPT-3 vaccination, measles-1 vaccination, ITN use, household wealth, education level of the household head | Child's age, sex, birthweight, healthcard ownership, DPT-3 vaccination, measles-1 vaccination, ITN use, household wealth, education level of the household head | Child's age, sex, birthweight, ITN use, healthcard ownership, receipt of vitamin A, DPT-3 vaccination, measles-1 vaccination, household wealth, education level of the household head |
| Sierra Leone 2013 DHS | - | - | Child's age, sex, birthweight, healthcard ownership, receipt of vitamin A, DPT-3 vaccination, measles-1 vaccination, household wealth, education level of the household head | Child's age, sex, birthweight, healthcard ownership, receipt of vitamin A, DPT-3 vaccination, measles-1 vaccination, smoking in house, main household cooking fuel, household wealth, education level of the household head | Child's age, sex, birthweight, healthcard ownership, DPT-3 vaccination, measles-1 vaccination, ITN use, household wealth, education level of the household head | Child's age, sex, birthweight, healthcard ownership, DPT-3 vaccination, measles-1 vaccination, ITN use, household wealth, education level of the household head | Child's age, sex, birthweight, healthcard ownership, DPT-3 vaccination, measles-1 vaccination, ITN use, household wealth, education level of the household head | Child's age, sex, birthweight, ITN use, healthcard ownership, receipt of vitamin A, DPT-3 vaccination, measles-1 vaccination, household wealth, education level of the household head |

**Table B.** Surveys included (n=74) and covariables adjusted for in the analysis of house type

| **Survey** | **Malaria infection (microscopy)** | **Malaria infection (RDT)** | **Diarrhoea** | **Acute respiratory infection** | **Low height-for-age** | **Low weight-for-height** | **Low weight-for-age** | **Anaemia** |
|---|---|---|---|---|---|---|---|---|
| Sierra Leone 2016 MIS | Child's age, sex, ITN use, IRS in the past 12 months, household wealth | Child's age, sex, ITN use, IRS in the past 12 months, household wealth | - | - | - | - | - | - |
| Tanzania 2010 DHS | - | - | Child's age, sex, birthweight, healthcard ownership, receipt of vitamin A, DPT-3 vaccination, measles-1 vaccination, household wealth, education level of the household head | Child's age, sex, birthweight, healthcard ownership, receipt of vitamin A, DPT-3 vaccination, measles-1 vaccination, main household cooking fuel, household wealth, education level of the household head | Child's age, sex, birthweight, healthcard ownership, DPT-3 vaccination, measles-1 vaccination, ITN use, household wealth, education level of the household head | Child's age, sex, birthweight, healthcard ownership, DPT-3 vaccination, measles-1 vaccination, ITN use, household wealth, education level of the household head | Child's age, sex, birthweight, healthcard ownership, DPT-3 vaccination, measles-1 vaccination, ITN use, household wealth, education level of the household head | Child's age, sex, birthweight, ITN use, healthcard ownership, receipt of vitamin A, DPT-3 vaccination, measles-1 vaccination, household wealth, education level of the household head |
| Tanzania 2012 AIS | Child's age, sex, ITN use, IRS in the past 12 months, household wealth, education level of the household head | Child's age, sex, ITN use, IRS in the past 12 months, household wealth, education level of the household head | - | - | - | - | - | - |
| Tanzania 2017 MIS | - | Child's age, sex, ITN use, household wealth | - | - | - | - | - | - |
| Togo 2013 DHS | Child's age, sex, ITN use, household wealth, education level of the household head | Child's age, sex, ITN use, household wealth, education level of the household head | Child's age, sex, birthweight, healthcard ownership, receipt of vitamin A, DPT-3 vaccination, measles-1 vaccination, household wealth, education level of the household head | Child's age, sex, birthweight, healthcard ownership, receipt of vitamin A, DPT-3 vaccination, measles-1 vaccination, smoking in house, main household cooking fuel, household wealth, | Child's age, sex, birthweight, healthcard ownership, DPT-3 vaccination, measles-1 vaccination, ITN use, household wealth, education level of the household head | Child's age, sex, birthweight, healthcard ownership, DPT-3 vaccination, measles-1 vaccination, ITN use, household wealth, education level of the household head | Child's age, sex, birthweight, healthcard ownership, DPT-3 vaccination, measles-1 vaccination, ITN use, household wealth, education level of the household head | Child's age, sex, birthweight, ITN use, healthcard ownership, receipt of vitamin A, DPT-3 vaccination, measles-1 vaccination, household wealth, education level of the household head |

**Table B.** Surveys included (n=74) and covariables adjusted for in the analysis of house type

| Survey | Malaria infection (microscopy) | Malaria infection (RDT) | Diarrhoea | Acute respiratory infection | Low height-for-age | Low weight-for-height | Low weight-for-age | Anaemia |
|---|---|---|---|---|---|---|---|---|
| | | | | education level of the household head | | | | |
| Uganda 2006 DHS | - | - | Child's age, sex, birthweight, healthcard ownership, receipt of vitamin A, DPT-3 vaccination, measles-1 vaccination, household wealth, education level of the household head | Child's age, sex, birthweight, healthcard ownership, receipt of vitamin A, DPT-3 vaccination, measles-1 vaccination, main household cooking fuel, household wealth, education level of the household head | Child's age, sex, birthweight, healthcard ownership, DPT-3 vaccination, measles-1 vaccination, ITN use, household wealth, education level of the household head | Child's age, sex, birthweight, healthcard ownership, DPT-3 vaccination, measles-1 vaccination, ITN use, household wealth, education level of the household head | Child's age, sex, birthweight, healthcard ownership, DPT-3 vaccination, measles-1 vaccination, ITN use, household wealth, education level of the household head | Child's age, sex, birthweight, ITN use, healthcard ownership, receipt of vitamin A, DPT-3 vaccination, measles-1 vaccination, household wealth, education level of the household head |
| Uganda 2009 MIS | Child's age, sex, ITN use, household wealth | Child's age, sex, ITN use, household wealth | - | - | - | - | - | - |
| Uganda 2014 MIS | Child's age, sex, ITN use, IRS in the past 12 months, household wealth | Child's age, sex, ITN use, IRS in the past 12 months, household wealth | - | - | - | - | - | - |

**Table B.** Surveys included (n=74) and covariables adjusted for in the analysis of house type

| Survey | Malaria infection (microscopy) | Malaria infection (RDT) | Diarrhoea | Acute respiratory infection | Low height-for-age | Low weight-for-height | Low weight-for-age | Anaemia |
|---|---|---|---|---|---|---|---|---|
| Uganda 2016 DHS | - | Child's age, sex, ITN use, household wealth, education level of the household head | Child's age, sex, birthweight, healthcard ownership, receipt of vitamin A, DPT-3 vaccination, measles-1 vaccination, rotavirus-2 vaccination, household wealth, education level of the household head | Child's age, sex, birthweight, healthcard ownership, receipt of vitamin A, DPT-3 vaccination, measles-1 vaccination, pneumococcal-3 vaccination, smoking in house, main household cooking fuel, household wealth, education level of the household head | Child's age, sex, birthweight, healthcard ownership, DPT-3 vaccination, measles-1 vaccination, ITN use, household wealth, education level of the household head | Child's age, sex, birthweight, healthcard ownership, DPT-3 vaccination, measles-1 vaccination, ITN use, household wealth, education level of the household head | Child's age, sex, birthweight, healthcard ownership, DPT-3 vaccination, measles-1 vaccination, ITN use, household wealth, education level of the household head | Child's age, sex, birthweight, ITN use, healthcard ownership, receipt of vitamin A, DPT-3 vaccination, measles-1 vaccination, rotavirus-2 vaccination, pneumococcal-3 vaccination, household wealth, education level of the household head |
| Zambia 2007 DHS | - | - | Child's age, sex, birthweight, healthcard ownership, receipt of vitamin A, DPT-3 vaccination, measles-1 vaccination, household wealth, education level of the household head | Child's age, sex, birthweight, healthcard ownership, receipt of vitamin A, DPT-3 vaccination, measles-1 vaccination, main household cooking fuel, household wealth, education level of the household head | Child's age, sex, birthweight, healthcard ownership, DPT-3 vaccination, measles-1 vaccination, ITN use, household wealth, education level of the household head | Child's age, sex, birthweight, healthcard ownership, DPT-3 vaccination, measles-1 vaccination, ITN use, household wealth, education level of the household head | Child's age, sex, birthweight, healthcard ownership, DPT-3 vaccination, measles-1 vaccination, ITN use, household wealth, education level of the household head | - |

**Table B.** Surveys included (n=74) and covariables adjusted for in the analysis of house type

| Survey | Malaria infection (microscopy) | Malaria infection (RDT) | Diarrhoea | Acute respiratory infection | Low height-for-age | Low weight-for-height | Low weight-for-age | Anaemia |
|---|---|---|---|---|---|---|---|---|
| Zambia 2013 DHS | - | - | Child's age, sex, birthweight, healthcard ownership, receipt of vitamin A, DPT-3 vaccination, measles-1 vaccination, household wealth, education level of the household head | Child's age, sex, birthweight, healthcard ownership, receipt of vitamin A, DPT-3 vaccination, measles-1 vaccination, smoking in house, main household cooking fuel, household wealth, education level of the household head | Child's age, sex, birthweight, healthcard ownership, DPT-3 vaccination, measles-1 vaccination, ITN use, household wealth, education level of the household head | Child's age, sex, birthweight, healthcard ownership, DPT-3 vaccination, measles-1 vaccination, ITN use, household wealth, education level of the household head | Child's age, sex, birthweight, healthcard ownership, DPT-3 vaccination, measles-1 vaccination, ITN use, household wealth, education level of the household head | - |
| Zimbabwe 2005 DHS | - | - | Child's age, sex, birthweight, healthcard ownership, receipt of vitamin A, DPT-3 vaccination, measles-1 vaccination, household wealth, education level of the household head | Child's age, sex, birthweight, healthcard ownership, receipt of vitamin A, DPT-3 vaccination, measles-1 vaccination, main household cooking fuel, household wealth, education level of the household head | Child's age, sex, birthweight, healthcard ownership, DPT-3 vaccination, measles-1 vaccination, ITN use, household wealth, education level of the household head | Child's age, sex, birthweight, healthcard ownership, DPT-3 vaccination, measles-1 vaccination, ITN use, household wealth, education level of the household head | Child's age, sex, birthweight, healthcard ownership, DPT-3 vaccination, measles-1 vaccination, ITN use, household wealth, education level of the household head | Child's age, sex, birthweight, ITN use, healthcard ownership, receipt of vitamin A, DPT-3 vaccination, measles-1 vaccination, household wealth, education level of the household head |

**Table B.** Surveys included (n=74) and covariables adjusted for in the analysis of house type

| **Survey** | **Malaria infection (microscopy)** | **Malaria infection (RDT)** | **Diarrhoea** | **Acute respiratory infection** | **Low height-for-age** | **Low weight-for-height** | **Low weight-for-age** | **Anaemia** |
|---|---|---|---|---|---|---|---|---|
| Zimbabwe 2010 DHS | - | - | Child's age, sex, birthweight, healthcard ownership, receipt of vitamin A, DPT-3 vaccination, measles-1 vaccination, household wealth, education level of the household head | Child's age, sex, birthweight, healthcard ownership, receipt of vitamin A, DPT-3 vaccination, measles-1 vaccination, smoking in house, main household cooking fuel, household wealth, education level of the household head | Child's age, sex, birthweight, healthcard ownership, DPT-3 vaccination, measles-1 vaccination, ITN use, household wealth, education level of the household head | Child's age, sex, birthweight, healthcard ownership, DPT-3 vaccination, measles-1 vaccination, ITN use, household wealth, education level of the household head | Child's age, sex, birthweight, healthcard ownership, DPT-3 vaccination, measles-1 vaccination, ITN use, household wealth, education level of the household head | Child's age, sex, birthweight, ITN use, healthcard ownership, receipt of vitamin A, DPT-3 vaccination, measles-1 vaccination, household wealth, education level of the household head |
| Zimbabwe 2015 DHS | - | - | Child's age, sex, birthweight, healthcard ownership, receipt of vitamin A, DPT-3 vaccination, measles-1 vaccination, rotavirus-2 vaccination, household wealth, education level of the household head | Child's age, sex, birthweight, healthcard ownership, receipt of vitamin A, DPT-3 vaccination, measles-1 vaccination, pneumococcal-3 vaccination, smoking in house, main household cooking fuel, household wealth, education level of the household head | Child's age, sex, birthweight, healthcard ownership, DPT-3 vaccination, measles-1 vaccination, ITN use, household wealth, education level of the household head | Child's age, sex, birthweight, healthcard ownership, DPT-3 vaccination, measles-1 vaccination, ITN use, household wealth, education level of the household head | Child's age, sex, birthweight, healthcard ownership, DPT-3 vaccination, measles-1 vaccination, ITN use, household wealth, education level of the household head | Child's age, sex, birthweight, ITN use, healthcard ownership, receipt of vitamin A, DPT-3 vaccination, measles-1 vaccination, rotavirus-2 vaccination, pneumococcal-3 vaccination, household wealth, education level of the household head |

Birthweight was modelled as a categorical variable comparing normal (2500g or greater) with low (less than 2500g) birthweight.
DPT: diphtheria-pertussis-tetanus; ITN: insecticide-treated net; RDT: rapid diagnostic test.
