# Supplementary material for: Housing and child health in sub-Saharan Africa: A cross-sectional analysis
Source: PLoS Med. 2020 Mar 23;17(3):e1003055. doi: 10.1371/journal.pmed.1003055 (PMC7089421; doi:10.1371/journal.pmed.1003055)
Supplement: S4 Text — (PDF) [file pmed.1003055.s004.pdf]

S4. Text. Characteristics of included surveys and prevalence of health outcomes by survey

Table A. Household-level characteristics of included surveys

Survey	N	Urban residence (%)	Improved drinking water source (%)	Improved sanitation (%)	House built with finished materials (%)	Improved house (%)	No smoking inside home (%)	Modern cooking fuel (%)	Not crowded (≤3 people / bedroom) (%)	IRS in past 12 months (%)	Household head attended secondary education (%)
Angola 2011 MIS	8391	39.5	48.1	44.9	45.6	15.0	-	39.9	48.8	-	-
Angola 2015 DHS	16109	55.1	50.4	56.1	43.7	15.0	79.9	40.3	58.4	1.5	37.1
Benin 2001 DHS	5769	35.2	53.3	17.0	54.3	-	-	0.8	-	-	17.0
Benin 2006 DHS	17511	41.3	70.3	17.2	56.6	7.6	-	3.5	60.4	-	19.2
Benin 2012 DHS	17422	40.8	77.2	29.1	60.8	14.3	88.3	4.6	53.3	7.5	20.4
Burkina Faso 2010 DHS	14424	30.6	79.0	32.9	48.7	18.7	72.9	5.1	67.6	1.5	10.9
Burkina Faso 2014 MIS	6448	20.4	76.8	42.6	45.1	17.1	-	6.7	60.9	0.8	-
Burundi 2010 DHS	8596	19.5	76.1	44.3	42.8	14.5	67.6	0.2	68.1	0.6	11.7
Burundi 2012 MIS	4866	18.1	80.5	77.7	42.9	23.7	-	0.0	71.2	4.8	11.2
Burundi 2016 DHS	15977	18.8	83.0	54.8	54.7	25.1	77.1	0.2	71.1	0.9	13.1
Cameroon 2011 DHS	14214	47.2	68.0	56.5	60.9	31.0	-	16.4	74.0	3.2	39.0
Comoros 2012 DHS	4482	42.2	89.3	38.2	80.4	20.4	74.7	4.4	67.0	14.2	32.5
Congo 2005 DHS	5879	65.5	71.5	20.5	70.5	12.0	-	12.6	59.3	-	63.0
Congo 2011 DHS	11632	27.1	53.1	23.1	53.5	10.9	76.4	7.5	60.1	-	58.6
Cote d'Ivoire 2012 DHS	9686	41.4	79.0	45.5	74.2	23.3	74.3	11.7	61.0	1.4	22.1
DRC 2013 DHS	18171	29.9	40.3	37.6	19.8	5.6	69.6	1.7	56.9	-	52.8
Ethiopia 2016 DHS	16650	31.4	69.2	25.4	27.5	9.9	82.9	9.4	36.9	-	19.7
Gabon 2012 DHS	9755	54.8	80.4	38.8	70.7	26.5	68.1	60.0	80.7	3.2	50.2
Gambia 2013 DHS	6217	49.8	90.4	58.2	79.1	33.0	71.6	2.3	67.2	42.0	25.4
Ghana 2008 DHS	11778	43.9	77.6	65.4	79.8	30.2	-	12.4	63.2	-	55.8
Ghana 2014 DHS	11835	50.2	65.9	68.0	89.3	25.8	86.1	21.0	65.9	18.4	57.6
Ghana 2016 MIS	5841	48.2	61.1	66.3	86.2	22.2	-	22.0	64.2	13.6	-
Guinea 2012 DHS	7109	35.2	73.4	45.0	58.7	24.8	75.1	0.4	64.9	1.7	19.3
Kenya 2008 DHS	9057	32.1	64.3	50.0	49.3	22.5	-	9.3	58.4	-	34.4
Kenya 2014 DHS	36430	38.2	64.5	47.9	49.0	19.9	84.9	7.3	57.6	2.5	33.5
Kenya 2015 MIS	6481	46.1	63.3	54.5	55.2	22.9	-	-	61.3	-	-

Table A. Household-level characteristics of included surveys

Survey	N	Urban residence (%)	Improved drinking water source (%)	Improved sanitation (%)	House built with finished materials (%)	Improved house (%)	No smoking inside home (%)	Modern cooking fuel (%)	Not crowded (≤3 people / bedroom) (%)	IRS in past 12 months (%)	Household head attended secondary education (%)
Lesotho 2009 DHS	9396	22.8	78.0	33.9	57.4	14.3	-	25.3	45.3	-	23.3
Lesotho 2014 DHS	9402	29.8	83.3	67.2	62.1	31.7	73.1	32.2	59.1	-	28.3
Liberia 2011 MIS	4162	46.0	70.4	28.1	46.9	9.4	-	0.2	47.7	10.7	-
Liberia 2013 DHS	9333	37.0	64.9	33.6	37.9	10.2	83.0	0.1	59.9	10.1	40.4
Liberia 2016 MIS	4218	46.8	67.5	36.9	49.8	10.0	-	0.4	54.4	0.8	-
Madagascar 2008 DHS	17857	25.2	43.8	7.4	29.8	3.7	-	1.1	34.2	-	29.1
Madagascar 2011 MIS	8094	25.6	46.8	15.1	31.1	5.9	-	0.8	34.7	46.1	-
Madagascar 2013 MIS	8574	25.6	45.3	16.4	29.0	6.1	-	0.9	38.5	35.5	-
Malawi 2010 DHS	24825	11.7	79.8	11.3	30.5	5.1	-	1.4	58.2	-	19.9
Malawi 2012 MIS	3404	31.0	82.6	25.1	41.2	12.2	-	4.1	60.6	8.5	-
Malawi 2014 MIS	3405	35.6	85.6	19.8	49.4	12.6	-	2.9	64.2	6.0	-
Malawi 2015 DHS	26361	18.9	87.0	83.3	46.6	29.6	83.7	2.0	64.5	6.1	27.6
Malawi 2017 MIS	3729	40.0	88.8	27.0	59.3	17.9	-	4.0	66.1	-	-
Mali 2012 DHS	10107	27.4	67.9	43.8	31.4	17.0	79.5	0.7	68.2	8.4	14.1
Mali 2015 MIS	4240	25.4	70.5	44.3	39.8	18.6	-	0.5	62.4	6.4	-
Mozambique 2011 DHS	13919	36.6	58.3	22.9	33.3	9.1	73.3	5.4	66.1	21.3	17.2
Mozambique 2015 AIS	7169	43.1	67.6	17.0	42.7	11.0	-	5.5	64.2	14.8	21.9
Namibia 2006 DHS	9200	42.3	88.1	44.0	60.4	30.8	-	36.2	74.7	-	44.4
Namibia 2013 DHS	9849	48.4	87.4	47.9	71.2	35.0	72.4	41.6	77.5	17.5	53.9
Niger 2012 DHS	10750	27.7	69.7	27.4	15.1	7.8	-	2.2	44.3	1.0	10.4
Nigeria 2008 DHS	34070	31.5	52.6	49.0	61.0	18.3	-	1.5	66.7	-	38.1
Nigeria 2010 MIS	5895	33.0	55.6	43.8	62.4	16.3	-	1.3	66.4	1.0	39.0
Nigeria 2013 DHS	38522	41.2	58.3	52.6	67.8	21.8	92.9	3.0	67.3	2.2	42.7
Nigeria 2015 MIS	7744	40.9	61.9	51.5	70.8	20.9	-	6.0	63.6	1.8	45.9
Rwanda 2010 DHS	12540	16.0	74.2	75.0	49.6	25.1	77.7	0.1	69.3	-	11.7
Rwanda 2015 DHS	12698	22.8	74.2	71.7	36.3	22.3	80.8	0.5	72.9	-	13.6
Rwanda 2017 MIS	5041	23.3	76.9	82.8	42.5	28.5	-	2.3	82.1	18.0	-

Table A. Household-level characteristics of included surveys

Survey	N	Urban residence (%)	Improved drinking water source (%)	Improved sanitation (%)	House built with finished materials (%)	Improved house (%)	No smoking inside home (%)	Modern cooking fuel (%)	Not crowded (≤3 people / bedroom) (%)	IRS in past 12 months (%)	Household head attended secondary education (%)
Senegal 2008 MIS	10651	31.0	65.4	46.6	52.2	21.2	-	17.2	59.6	-	-
Senegal 2010 DHS	7904	37.5	69.5	44.3	59.3	23.0	-	17.4	61.6	12.4	11.5
Senegal 2012 DHS	4177	39.2	66.9	51.3	63.4	24.3	58.4	14.5	59.3	15.5	12.8
Senegal 2014 DHS	4233	39.1	69.5	49.1	64.1	24.5	52.4	12.3	61.0	11.2	11.5
Senegal 2015 DHS	4511	38.9	65.5	49.1	69.4	25.9	-	12.4	62.5	6.9	12.0
Senegal 2016 DHS	4440	39.0	72.0	52.5	68.6	26.1	-	15.2	62.9	7.8	12.5
Sierra Leone 2008 DHS	7284	40.6	55.2	46.7	41.6	15.4	-	0.1	67.3	-	26.0
Sierra Leone 2013 DHS	12629	36.2	58.8	50.0	50.2	15.6	62.8	0.1	62.2	4.4	24.6
Sierra Leone 2016 MIS	6719	29.5	62.2	42.3	52.2	16.6	-	0.5	65.8	1.4	-
Swaziland 2006 DHS	4843	38.8	71.5	41.2	86.6	29.2	-	37.9	72.1	-	44.6
Tanzania 2004 DHS	9735	22.2	52.6	6.8	34.8	-	-	0.4	-	-	10.9
Tanzania 2010 DHS	9623	23.0	52.4	25.5	43.7	11.2	-	0.9	65.5	-	14.0
Tanzania 2012 AIS	10040	22.5	60.5	37.1	46.7	17.4	-	1.0	67.0	23.3	13.0
Tanzania 2017 MIS	9330	28.3	61.1	58.7	56.5	23.7	-	3.1	67.8	-	-
Togo 2013 DHS	9549	38.1	63.2	38.3	78.8	18.9	82.0	6.1	63.0	-	36.8
Uganda 2006 DHS	8870	15.7	69.3	27.1	27.7	8.1	-	0.3	46.0	-	22.7
Uganda 2009 MIS	4421	14.9	72.7	35.4	34.3	12.3	-	1.2	52.1	-	-
Uganda 2014 MIS	5345	20.9	77.1	31.2	38.5	12.6	-	0.7	51.0	8.1	-
Uganda 2016 DHS	19588	22.8	77.0	34.4	40.7	14.9	85.5	0.9	58.1	-	30.8
Zambia 2007 DHS	7164	37.6	42.5	33.0	41.3	12.3	-	13.5	46.9	-	37.9
Zambia 2013 DHS	15920	43.7	62.1	41.5	51.2	18.0	81.4	10.5	53.5	34.6	44.5
Zimbabwe 2005 DHS	9285	32.9	77.3	63.4	67.2	35.9	-	31.7	61.9	-	48.9
Zimbabwe 2010 DHS	9756	34.1	78.5	63.1	71.2	35.7	76.6	29.8	64.1	19.7	53.5
Zimbabwe 2015 DHS	10534	41.2	81.6	69.7	79.6	43.0	82.1	35.0	68.5	21.8	61.7

AIS: AIDS Indicator Survey; DHS: Demographic and Health Survey; DRC: Democratic Republic of the Congo; IRS: indoor residual spraying; MIS: Malaria Indicator Survey

Table B. Child-level characteristics of included surveys

Survey	N	Mean age in years	Male (%)	Slept under ITN previous night (%)	Low birth weight (<2500g) (%)	Has health card (%)	Received vitamin A (%)	Received DPT-3 (%)	Received measles-1 vaccine (%)	Received rotavirus-2 vaccine (%)	Received pneumococcal-3 vaccine (%)
Angola 2011 MIS	9681	2.4	49.9	24.5	-	-	-	-	-	-	-
Angola 2015 DHS	18311	2.5	49.6	21.1	10.7	53.3	-	30.1	39.5	28.7	23.3
Benin 2001 DHS	6250	2.5	50.1	-	13.0	85.4	18.5	61.5	54.8	-	-
Benin 2006 DHS	19444	2.5	50.4	30.9	12.0	85.2	61.0	59.9	53.9	-	-
Benin 2012 DHS	17489	2.6	51.0	69.8	12.3	86.5	47.8	59.1	60.6	-	-
Burkina Faso 2010 DHS	16969	2.4	50.9	48.5	12.9	86.0	61.4	79.2	71.6	-	-
Burkina Faso 2014 MIS	8419	2.5	50.9	75.2	-	-	-	-	-	-	-
Burundi 2010 DHS	9025	2.4	50.3	46.3	9.6	71.4	78.1	88.1	80.3	-	-
Burundi 2012 MIS	4985	2.4	49.7	53.2	-	-	-	-	-	-	-
Burundi 2016 DHS	15544	2.5	50.3	38.7	8.9	80.0	64.8	86.2	69.1	81.8	82.4
Cameroon 2011 DHS	14276	2.4	49.7	13.2	7.1	80.1	70.1	63.9	62.0	-	-
Comoros 2012 DHS	3933	2.4	50.0	44.2	16.1	88.7	47.1	61.5	61.9	-	-
Congo 2005 DHS	5753	2.4	50.3	6.3	11.3	78.7	68.1	62.9	57.6	-	-
Congo 2011 DHS	11145	2.4	50.6	10.6	9.9	66.9	61.7	48.5	58.2	-	-
Cote d'Ivoire 2012 DHS	9742	2.5	50.0	37.4	13.5	91.2	58.8	56.9	55.4	-	-
DRC 2013 DHS	22059	2.4	49.6	52.3	6.5	50.4	61.8	50.5	59.6	-	-
Eswatini 2006 DHS	3713	2.5	49.4	0.8	7.0	97.9	80.5	84.8	76.2	-	-
Ethiopia 2016 DHS	12794	2.5	51.1	-	11.0	55.6	44.8	45.2	41.1	46.4	41.3
Gabon 2012 DHS	7446	2.3	49.9	44.2	14.2	89.0	60.5	34.9	61.6	-	-
Gambia 2013 DHS	10701	2.4	50.9	45.3	10.9	96.1	63.6	77.2	73.1	-	-
Ghana 2008 DHS	7411	2.5	50.8	39.0	9.7	95.2	68.2	75.7	72.5	-	-
Ghana 2014 DHS	7341	2.4	51.9	47.4	9.6	96.2	59.3	78.0	73.9	-	-
Ghana 2016 MIS	4159	2.5	50.8	54.9	-	-	-	-	-	-	-
Guinea 2012 DHS	8531	2.5	51.4	27.1	8.2	76.8	38.7	44.4	54.8	-	-
Kenya 2008 DHS	7231	2.4	51.4	52.2	5.7	88.6	46.4	72.4	68.8	-	-
Kenya 2014 DHS	26253	2.5	50.6	52.4	7.2	89.6	67.0	81.5	72.4	-	-
Kenya 2015 MIS	4724	2.6	50.3	56.1	-	-	-	-	-	-	-

Table B. Child-level characteristics of included surveys

Survey	N	Mean age in years	Male (%)	Slept under ITN previous night (%)	Low birth weight (<2500g) (%)	Has health card (%)	Received vitamin A (%)	Received DPT-3 (%)	Received measles-1 vaccine (%)	Received rotavirus-2 vaccine (%)	Received pneumococcal-3 vaccine (%)
Lesotho 2009 DHS	5987	2.5	49.4	-	8.9	92.3	58.0	72.8	67.8	-	-
Lesotho 2014 DHS	5181	2.6	49.8	-	10.2	97.9	53.6	79.0	74.2	-	-
Liberia 2011 MIS	4340	2.5	50.2	36.5	-	-	-	-	-	-	-
Liberia 2013 DHS	9724	2.5	51.2	36.8	10.3	83.4	53.2	55.1	58.0	-	-
Liberia 2016 MIS	3926	2.5	49.9	42.3	-	77.4	-	-	50.3	25.3	54.0
Madagascar 2008 DHS	15763	2.5	50.6	46.4	11.9	78.9	88.6	65.3	59.9	-	-
Madagascar 2011 MIS	8109	2.5	50.9	74.7	-	-	-	-	-	-	-
Madagascar 2013 MIS	7306	2.5	51.0	53.8	-	-	-	-	-	-	-
Malawi 2010 DHS	24280	2.5	49.4	42.8	10.8	86.1	82.2	85.3	78.8	-	-
Malawi 2012 MIS	2813	2.4	47.5	57.2	-	-	-	-	-	-	-
Malawi 2014 MIS	2621	2.4	50.1	68.9	-	-	-	-	-	-	-
Malawi 2015 DHS	21414	2.6	49.9	43.8	11.8	84.4	61.9	82.1	68.4	83.6	79.0
Malawi 2017 MIS	2950	2.5	49.2	66.6	-	-	-	-	-	-	-
Mali 2012 DHS	12882	2.5	50.9	68.2	15.0	78.9	56.4	55.1	61.1	-	-
Mali 2015 MIS	9539	2.5	50.4	70.8	-	-	-	-	-	-	-
Mozambique 2011 DHS	12683	2.4	49.9	33.1	12.8	85.6	69.3	72.6	71.6	-	-
Mozambique 2015 AIS	6483	2.4	49.3	48.3	-	89.1	-	74.0	73.1	-	-
Namibia 2006 DHS	6774	2.4	49.8	10.9	12.9	93.8	75.6	77.1	69.7	-	-
Namibia 2013 DHS	6953	2.5	49.5	6.1	12.1	94.6	82.0	77.5	73.5	-	-
Niger 2012 DHS	15291	2.5	50.4	22.7	10.4	78.8	58.0	59.1	59.4	-	-
Nigeria 2008 DHS	31634	2.4	50.9	6.1	8.0	41.4	70.3	30.1	36.0	-	-
Nigeria 2010 MIS	6941	2.4	50.9	29.8	-	-	-	-	-	-	-
Nigeria 2013 DHS	35364	2.4	50.7	18.2	7.0	49.2	40.1	36.7	38.0	-	-
Nigeria 2015 MIS	8290	2.5	50.4	41.3	-	-	-	-	-	-	-
Rwanda 2010 DHS	10697	2.6	50.8	67.8	5.8	83.9	87.2	91.2	81.6	-	-
Rwanda 2015 DHS	9505	2.5	50.5	65.9	5.8	93.5	79.8	91.8	30.9	61.7	-
Rwanda 2017 MIS	3548	2.4	52.0	68.5	-	-	-	-	-	-	-

Table B. Child-level characteristics of included surveys

Survey	N	Mean age in years	Male (%)	Slept under ITN previous night (%)	Low birth weight (<2500g) (%)	Has health card (%)	Received vitamin A (%)	Received DPT-3 (%)	Received measles-1 vaccine (%)	Received rotavirus-2 vaccine (%)	Received pneumococcal-3 vaccine (%)
Senegal 2008 MIS	23105	2.5	51.1	32.1	-	-	-	-	-	-	-
Senegal 2010 DHS	15752	2.5	51.4	44.0	14.4	90.3	74.2	73.4	71.1	-	-
Senegal 2012 DHS	8746	2.5	50.0	48.6	13.3	88.1	77.3	79.4	68.0	-	-
Senegal 2014 DHS	8432	2.4	50.2	49.2	13.3	92.3	83.5	79.9	68.7	-	-
Senegal 2015 DHS	8553	2.4	49.9	56.3	12.1	93.7	83.8	79.7	66.5	-	-
Senegal 2016 DHS	8380	2.5	51.2	67.4	11.3	90.9	73.2	79.9	66.4	-	-
Sierra Leone 2008 DHS	7426	2.4	50.1	27.3	10.8	83.4	47.4	51.6	51.4	-	-
Sierra Leone 2013 DHS	14958	2.6	49.5	49.8	6.1	89.7	80.2	68.5	70.7	-	-
Sierra Leone 2016 MIS	8460	2.5	50.4	44.3	-	-	-	-	-	-	-
Tanzania 2004 DHS	10142	2.4	50.2	17.8	7.3	87.9	38.0	40.0	67.1	-	-
Tanzania 2010 DHS	10107	2.5	49.5	61.3	6.1	92.2	59.1	80.7	71.1	-	-
Tanzania 2012 AIS	10921	2.4	50.2	68.9	-	-	-	-	-	-	-
Tanzania 2017 MIS	9623	2.5	50.5	52.3	-	-	-	-	-	-	-
Togo 2013 DHS	8583	2.5	50.4	43.4	9.3	91.2	81.5	76.2	66.5	-	-
Uganda 2006 DHS	10064	2.5	49.1	11.9	10.0	80.2	54.2	22.8	62.7	-	-
Uganda 2009 MIS	4940	2.5	50.1	31.9	-	-	-	-	-	-	-
Uganda 2014 MIS	6108	2.5	49.0	73.6	-	-	-	-	-	-	-
Uganda 2016 DHS	19453	2.6	50.4	58.5	9.5	81.7	57.4	69.7	59.4	4.8	58.1
Zambia 2007 DHS	7404	2.3	49.3	32.9	8.4	89.0	77.0	34.0	70.9	-	-
Zambia 2013 DHS	16657	2.5	50.6	40.0	8.3	87.8	68.6	80.4	74.0	-	-
Zimbabwe 2005 DHS	7284	2.6	50.4	3.1	9.4	90.5	45.9	52.7	52.2	-	-
Zimbabwe 2010 DHS	7187	2.4	50.2	10.6	8.4	87.8	58.7	65.4	65.1	-	-
Zimbabwe 2015 DHS	8082	2.6	49.6	9.2	9.4	88.5	61.1	75.1	61.0	42.7	72.7

AIS: AIDS Indicator Survey; DHS: Demographic and Health Survey; DRC: Democratic Republic of the Congo; DPT: diphtheria-pertussis-tetanus; ITN: insecticide-treated net; MIS: Malaria Indicator Survey.

Table C. Prevalence of health outcomes by survey

Survey	N	Malaria positive by microscopy, % (n)	Malaria positive by RDT, % (n)	Diarrhoea in past two weeks, % (n)	Cough with short and rapid breathing in past two weeks, % (n)	Low height-for-age, % (n)	Low weight-for-height, % (n)	Low weight-for-age, % (n)	Any anaemia, % (n)	Moderate to severe anaemia, % (n)
Angola 2011 MIS	9681	9.8 (3431)	12.6 (3432)	-	-	-	-	-	52.8 (3418)	26.4 (3418)
Angola 2015 DHS	18311	-	17.2 (8317)	14.2 (12746)	4.6 (12743)	31.6 (7453)	4.5 (7587)	23.0 (7453)	64.1 (6854)	34.3 (6854)
Benin 2001 DHS	6250	-	-	14.1 (4559)	48.6 (1167)	31.1 (4085)	7.3 (4865)	23.1 (4085)	79.2 (2568)	57.6 (2568)
Benin 2006 DHS	19444	-	-	9.7 (14184)	48.2 (2853)	38.2 (13221)	7.4 (13677)	23.0 (13221)	76.4 (4823)	52.1 (4823)
Benin 2012 DHS	17489	30.5 (4638)	27.6 (4695)	6.6 (12035)	42.9 (944)	41.6 (8423)	14.7 (10486)	27.2 (8423)	58.3 (3784)	31.8 (3784)
Burkina Faso 2010 DHS	16969	65.0 (6102)	75.6 (6125)	15.1 (13422)	43.2 (1398)	29.5 (6878)	13.3 (6891)	29.6 (6878)	88.2 (6286)	69.6 (6286)
Burkina Faso 2014 MIS	8419	47.7 (6117)	64.4 (6154)	-	-	-	-	-	86.5 (6155)	69.7 (6155)
Burundi 2010 DHS	9025	-	-	25.0 (7111)	79.9 (2630)	48.5 (3624)	5.2 (3634)	32.6 (3624)	43.3 (3265)	20.2 (3265)
Burundi 2012 MIS	4985	16.1 (3722)	20.6 (3750)	-	-	-	-	-	48.9 (3754)	25.6 (3754)
Burundi 2016 DHS	15544	25.7 (6924)	35.7 (6913)	21.6 (12274)	12.5 (12272)	47.5 (6355)	4.0 (6358)	34.9 (6355)	59.1 (5767)	34.1 (5767)
Cameroon 2011 DHS	14276	-	33.5 (6605)	20.5 (9981)	45.6 (3564)	27.8 (5923)	4.4 (6001)	16.9 (5923)	61.4 (5476)	34.3 (5476)
Comoros 2012 DHS	3933	-	-	16.5 (2852)	38.9 (504)	24.7 (2705)	10.1 (2727)	18.3 (2705)	-	-
Congo 2005 DHS	5753	-	-	15.0 (4154)	27.4 (1159)	24.1 (4349)	6.7 (4417)	13.3 (4349)	65.0 (2268)	36.1 (2268)
Congo 2011 DHS	11145	-	-	17.9 (8403)	33.7 (2357)	23.1 (4979)	4.8 (4988)	17.3 (4979)	66.0 (4457)	34.6 (4457)
Cote d'Ivoire 2012 DHS	9742	17.7 (4044)	46.5 (4215)	18.7 (6606)	42.6 (1410)	24.0 (3781)	6.3 (3844)	19.3 (3781)	74.8 (3403)	49.9 (3403)
DRC 2013 DHS	22059	26.3 (8186)	36.0 (8219)	16.8 (16532)	39.8 (5219)	39.1 (8962)	6.9 (8979)	27.9 (8962)	62.8 (8266)	38.3 (8266)
Eswatini 2006 DHS	3713	-	-	15.1 (2222)	53.8 (651)	23.8 (2784)	2.3 (2820)	7.3 (2784)	41.6 (2540)	20.0 (2540)
Ethiopia 2016 DHS	12794	-	-	11.1 (9682)	8.1 (9695)	31.4 (9463)	10.8 (9617)	30.3 (9463)	59.9 (8525)	36.0 (8525)
Gabon 2012 DHS	7446	-	-	18.6 (5144)	41.8 (1984)	19.9 (4107)	3.3 (4142)	11.0 (4107)	62.9 (3849)	33.5 (3849)
Gambia 2013 DHS	10701	0.5 (3481)	1.8 (3298)	17.7 (7441)	63.7 (1042)	21.2 (3504)	10.0 (3556)	23.1 (3504)	73.1 (3422)	48.8 (3422)
Ghana 2008 DHS	7411	-	-	20.5 (2679)	50.4 (593)	23.6 (2665)	8.4 (2677)	18.3 (2665)	80.0 (2467)	57.8 (2467)
Ghana 2014 DHS	7341	30.7 (3197)	42.2 (3191)	12.3 (5399)	49.2 (738)	14.7 (3069)	4.8 (3074)	14.7 (3069)	68.6 (2736)	41.8 (2736)
Ghana 2016 MIS	4159	23.0 (3078)	32.4 (3078)	-	-	-	-	-	65.7 (3078)	38.2 (3078)
Guinea 2012 DHS	8531	43.4 (3220)	45.6 (3215)	17.3 (6153)	65.7 (1199)	26.4 (3525)	9.7 (3531)	22.0 (3525)	75.0 (3237)	51.2 (3237)
Kenya 2008 DHS	7231	-	-	17.1 (5481)	47.5 (1470)	29.1 (5588)	7.1 (5634)	20.8 (5588)	-	-
Kenya 2014 DHS	26253	-	-	15.0 (19314)	46.7 (6908)	21.5 (20699)	5.2 (20793)	17.4 (20699)	-	-

Table C. Prevalence of health outcomes by survey

Survey	N	Malaria positive by microscopy, % (n)	Malaria positive by RDT, % (n)	Diarrhoea in past two weeks, % (n)	Cough with short and rapid breathing in past two weeks, % (n)	Low height-for-age, % (n)	Low weight-for-height, % (n)	Low weight-for-age, % (n)	Any anaemia, % (n)	Moderate to severe anaemia, % (n)
Kenya 2015 MIS	4724	5.9 (4105)	10.0 (4095)	-	-	-	-	-	34.9 (4100)	16.0 (4100)
Lesotho 2009 DHS	5987	-	-	12.1 (3306)	44.3 (1012)	33.1 (2254)	3.8 (2257)	17.1 (2254)	49.2 (2084)	23.8 (2084)
Lesotho 2014 DHS	5181	-	-	11.9 (2658)	31.9 (753)	27.9 (1924)	2.7 (1929)	14.4 (1924)	51.0 (1766)	26.0 (1766)
Liberia 2011 MIS	4340	32.0 (3081)	51.3 (3187)	-	-	-	-	-	74.5 (3207)	48.8 (3207)
Liberia 2013 DHS	9724	-	-	25.2 (6429)	46.5 (1885)	27.7 (3877)	5.9 (3880)	20.2 (3877)	-	-
Liberia 2016 MIS	3926	-	50.5 (2788)	-	-	-	-	-	78.7 (2838)	52.0 (2838)
Madagascar 2008 DHS	15763	-	-	8.8 (11315)	44.0 (1367)	43.4 (5490)	-	-	51.0 (5408)	20.1 (5408)
Madagascar 2011 MIS	8109	4.3 (6836)	6.4 (6874)	-	-	-	-	-	50.7 (6238)	24.4 (6238)
Madagascar 2013 MIS	7306	7.3 (6151)	8.2 (6232)	-	-	-	-	-	49.2 (5430)	24.1 (5430)
Malawi 2010 DHS	24280	-	-	17.2 (17894)	53.9 (5152)	40.6 (4927)	3.7 (4935)	17.4 (4927)	64.0 (4542)	40.1 (4542)
Malawi 2012 MIS	2813	24.8 (2112)	38.4 (2115)	-	-	-	-	-	66.7 (2121)	41.0 (2121)
Malawi 2014 MIS	2621	26.6 (1928)	30.1 (1921)	-	-	-	-	-	51.9 (1942)	26.4 (1942)
Malawi 2015 DHS	21414	-	-	21.1 (15802)	10.3 (15792)	28.6 (5677)	2.5 (5719)	16.1 (5677)	62.6 (5276)	35.3 (5276)
Malawi 2017 MIS	2950	17.7 (2502)	26.9 (2504)	-	-	-	-	-	58.1 (2313)	32.7 (2313)
Mali 2012 DHS	12882	50.2 (5646)	45.0 (5706)	9.0 (9130)	44.5 (703)	33.0 (4861)	11.6 (4888)	30.3 (4861)	80.7 (4742)	59.4 (4742)
Mali 2015 MIS	9539	34.7 (7347)	31.4 (7302)	-	-	-	-	-	85.2 (7300)	63.4 (7300)
Mozambique 2011 DHS	12683	29.9 (4898)	33.9 (4916)	10.7 (9813)	46.0 (1055)	33.7 (10302)	4.2 (10413)	17.0 (10302)	65.4 (4923)	39.1 (4923)
Mozambique 2015 AIS	6483	-	31.6 (4476)	11.3 (4834)	53.9 (1389)	-	-	-	60.1 (4481)	33.6 (4481)
Namibia 2006 DHS	6774	-	-	13.8 (3951)	49.9 (720)	24.4 (5156)	7.0 (5206)	21.9 (5156)	-	-
Namibia 2013 DHS	6953	-	-	19.5 (3850)	43.7 (1265)	19.4 (2589)	6.7 (2619)	17.3 (2589)	48.8 (2342)	23.7 (2342)
Niger 2012 DHS	15291	-	-	14.0 (11256)	52.4 (1590)	35.7 (5176)	15.8 (5224)	40.7 (5176)	74.4 (4827)	48.2 (4827)
Nigeria 2008 DHS	31634	-	-	10.8 (24174)	40.9 (2850)	38.3 (20862)	12.8 (20936)	28.3 (20862)	-	-
Nigeria 2010 MIS	6941	38.1 (5137)	47.2 (5147)	-	-	-	-	-	69.4 (5074)	45.2 (5074)
Nigeria 2013 DHS	35364	-	-	10.6 (27463)	41.5 (2737)	32.3 (26424)	14.7 (26463)	31.0 (26424)	-	-
Nigeria 2015 MIS	8290	27.2 (5753)	43.6 (6025)	-	-	-	-	-	67.5 (6029)	42.1 (6029)
Rwanda 2010 DHS	10697	1.5 (4950)	2.6 (4893)	13.4 (8237)	42.4 (1952)	36.6 (4380)	2.5 (4396)	15.3 (4380)	37.7 (4068)	13.6 (4068)

Table C. Prevalence of health outcomes by survey

Survey	N	Malaria positive by microscopy, % (n)	Malaria positive by RDT, % (n)	Diarrhoea in past two weeks, % (n)	Cough with short and rapid breathing in past two weeks, % (n)	Low height-for-age, % (n)	Low weight-for-height, % (n)	Low weight-for-age, % (n)	Any anaemia, % (n)	Moderate to severe anaemia, % (n)
Rwanda 2015 DHS	9505	2.3 (4338)	7.8 (4230)	12.3 (7321)	43.7 (1980)	32.0 (3811)	1.8 (3828)	12.0 (3811)	35.8 (3531)	15.3 (3531)
Rwanda 2017 MIS	3548	7.2 (3207)	11.4 (3154)	-	-	-	-	-	-	-
Senegal 2008 MIS	23105	6.7 (4138)	12.0 (4032)	-	-	-	-	-	80.2 (4217)	57.7 (4217)
Senegal 2010 DHS	15752	4.0 (4698)	3.4 (4716)	19.6 (11045)	60.4 (2064)	24.9 (3953)	8.6 (4306)	24.8 (3953)	77.6 (3887)	53.8 (3887)
Senegal 2012 DHS	8746	4.2 (7266)	4.7 (7316)	15.5 (6235)	55.4 (726)	16.6 (6497)	9.1 (6636)	22.5 (6497)	71.5 (5793)	46.7 (5793)
Senegal 2014 DHS	8432	1.9 (6762)	1.8 (6762)	20.4 (6192)	46.7 (452)	17.1 (6714)	6.4 (6807)	19.6 (6714)	60.5 (6239)	34.5 (6239)
Senegal 2015 DHS	8553	0.5 (6883)	1.1 (6895)	21.5 (6285)	50.0 (682)	17.3 (6818)	7.6 (6877)	21.8 (6818)	70.2 (6241)	42.6 (6241)
Senegal 2016 DHS	8380	1.4 (6703)	1.8 (6703)	17.2 (6143)	55.0 (584)	14.7 (6637)	7.2 (6714)	20.0 (6637)	68.5 (6083)	39.7 (6083)
Sierra Leone 2008 DHS	7426	-	-	12.2 (4520)	50.1 (921)	31.9 (2648)	9.4 (2680)	24.2 (2648)	75.2 (2516)	46.6 (2516)
Sierra Leone 2013 DHS	14958	-	-	12.0 (9699)	59.8 (1943)	32.1 (5104)	7.9 (5143)	20.4 (5104)	79.9 (5290)	53.6 (5290)
Sierra Leone 2016 MIS	8460	43.4 (7677)	57.7 (7666)	-	-	-	-	-	77.9 (6671)	52.1 (6671)
Tanzania 2004 DHS	10142	-	-	13.6 (7430)	32.0 (2065)	36.7 (7975)	3.6 (8019)	22.5 (7975)	70.7 (8124)	45.6 (8124)
Tanzania 2010 DHS	10107	-	-	14.1 (7158)	35.0 (1522)	34.0 (7605)	5.5 (7611)	22.1 (7605)	52.6 (7788)	28.0 (7788)
Tanzania 2012 AIS	10921	4.7 (7555)	9.9 (7695)	-	-	-	-	-	56.7 (7793)	29.0 (7793)
Tanzania 2017 MIS	9623	-	8.4 (7249)	-	-	-	-	-	61.2 (7262)	32.6 (7262)
Togo 2013 DHS	8583	39.5 (3888)	41.2 (3868)	16.3 (6365)	54.2 (1687)	23.5 (3531)	6.3 (3541)	21.3 (3531)	70.3 (3240)	44.6 (3240)
Uganda 2006 DHS	10064	-	-	27.0 (7134)	53.0 (3212)	33.0 (2670)	5.4 (2715)	21.5 (2670)	72.8 (2445)	50.4 (2445)
Uganda 2009 MIS	4940	43.6 (4011)	53.1 (3998)	-	-	-	-	-	62.9 (3972)	41.7 (3972)
Uganda 2014 MIS	6108	19.7 (4939)	32.8 (4903)	-	-	-	-	-	53.2 (4946)	29.2 (4946)
Uganda 2016 DHS	19453	-	33.7 (5500)	21.0 (13484)	15.4 (13486)	23.5 (5180)	3.2 (5266)	13.6 (5180)	53.6 (4806)	29.7 (4806)
Zambia 2007 DHS	7404	-	-	16.0 (5606)	36.7 (1424)	38.2 (5639)	5.2 (5647)	19.2 (5639)	-	-
Zambia 2013 DHS	16657	-	-	16.5 (12273)	30.3 (2833)	33.7 (12474)	5.5 (12495)	19.7 (12474)	-	-
Zimbabwe 2005 DHS	7284	-	-	13.1 (4538)	54.9 (1005)	29.0 (4979)	6.2 (5008)	16.4 (4979)	57.7 (4436)	30.4 (4436)
Zimbabwe 2010 DHS	7187	-	-	13.8 (4752)	47.4 (1061)	25.9 (5355)	2.9 (5414)	13.6 (5355)	56.5 (4362)	29.6 (4362)
Zimbabwe 2015 DHS	8082	-	-	16.9 (5244)	8.1 (5240)	20.4 (6142)	3.0 (6142)	11.1 (6142)	36.5 (5326)	14.8 (5326)

AIS: AIDS Indicator Survey; DHS: Demographic and Health Survey; DRC: Democratic Republic of the Congo; MIS: Malaria Indicator Survey; RDT: rapid diagnostic test.
