# Supplementary material for: Housing and child health in sub-Saharan Africa: A cross-sectional analysis
Source: PLoS Med. 2020 Mar 23;17(3):e1003055. doi: 10.1371/journal.pmed.1003055 (PMC7089421; doi:10.1371/journal.pmed.1003055)
Supplement: S5 Text — (PDF) [file pmed.1003055.s005.pdf]

S5. Text. Association between housing conditions and health in children aged 0-5 years in sub-Saharan Africa

Table A. Association between finished house construction materials and malaria, diarrhoea and acute respiratory infection in children aged 0-5 years in sub-Saharan Africa (adjusted results)

Survey	Malaria positive by microscopy		Malaria positive by RDT		Diarrhoea in past two weeks		Cough with short and rapid breathing in past two weeks	
	OR (95% CI)	p	OR (95% CI)	p	OR (95% CI)	p	OR (95% CI)	p
Angola 2011 MIS	1.14 (0.59, 2.21)	0.69	0.72 (0.38, 1.37)	0.31	-	-	-	-
Angola 2015 DHS	-	-	0.86 (0.65, 1.14)	0.30	-	-	-	-
Benin 2001 DHS	-	-	-	-	1.10 (0.78, 1.55)	0.60	-	-
Benin 2006 DHS	-	-	-	-	1.03 (0.81, 1.31)	0.82	1.02 (0.70, 1.49)	0.91
Benin 2012 DHS	0.78 (0.62, 0.98)	0.03	0.82 (0.65, 1.04)	0.11	1.03 (0.76, 1.39)	0.85	-	-
Burkina Faso 2010 DHS	1.04 (0.87, 1.24)	0.65	0.94 (0.77, 1.15)	0.56	0.94 (0.80, 1.12)	0.50	-	-
Burkina Faso 2014 MIS	0.94 (0.81, 1.09)	0.43	0.98 (0.83, 1.14)	0.77	-	-	-	-
Burundi 2010 DHS	-	-	-	-	0.88 (0.72, 1.08)	0.22	1.00 (0.66, 1.50)	0.99
Burundi 2012 MIS	0.73 (0.52, 1.03)	0.07	0.56 (0.40, 0.77)	<0.001	-	-	-	-
Burundi 2016 DHS	0.74 (0.63, 0.87)	<0.001	0.72 (0.61, 0.85)	<0.001	0.92 (0.79, 1.07)	0.26	1.04 (0.86, 1.26)	0.68
Cameroon 2011 DHS	-	-	0.93 (0.77, 1.13)	0.48	0.85 (0.63, 1.13)	0.26	1.51 (1.00, 2.29)	0.05
Comoros 2012 DHS	-	-	-	-	1.74 (1.07, 2.81)	0.02	1.73 (0.40, 7.43)	0.46
Congo 2005 DHS	-	-	-	-	0.81 (0.58, 1.13)	0.21	1.38 (0.74, 2.58)	0.32
Congo 2011 DHS	-	-	-	-	1.06 (0.86, 1.30)	0.60	0.84 (0.58, 1.22)	0.37
DRC 2013 DHS	0.79 (0.61, 1.01)	0.06	0.79 (0.62, 1.01)	0.06	1.20 (0.98, 1.46)	0.07	0.87 (0.65, 1.16)	0.35
Cote d'Ivoire 2012 DHS	0.80 (0.63, 1.02)	0.07	0.88 (0.71, 1.09)	0.23	0.76 (0.56, 1.02)	0.06	0.67 (0.33, 1.38)	0.28
Eswatini 2006 DHS	-	-	-	-	0.72 (0.45, 1.14)	0.16	0.73 (0.35, 1.52)	0.40
Ethiopia 2016 DHS	-	-	-	-	1.16 (0.61, 2.19)	0.65	1.82 (0.74, 4.48)	0.19
Gabon 2012 DHS	-	-	-	-	1.03 (0.79, 1.34)	0.82	0.83 (0.57, 1.21)	0.33
Gambia 2013 DHS	1.06 (0.21, 5.30)	0.94	0.44 (0.17, 1.12)	0.08	1.02 (0.76, 1.36)	0.90	-	-
Ghana 2008 DHS	-	-	-	-	0.43 (0.11, 1.74)	0.24	-	-
Ghana 2014 DHS	0.84 (0.61, 1.15)	0.28	0.86 (0.61, 1.21)	0.37	1.14 (0.69, 1.87)	0.61	0.88 (0.17, 4.63)	0.88
Ghana 2016 MIS	0.97 (0.69, 1.37)	0.88	0.89 (0.64, 1.23)	0.48	-	-	-	-
Guinea 2012 DHS	1.02 (0.80, 1.29)	0.88	0.93 (0.72, 1.19)	0.56	0.99 (0.66, 1.47)	0.96	0.89 (0.32, 2.51)	0.83
Kenya 2008 DHS	-	-	-	-	0.69 (0.38, 1.25)	0.22	5.18 (0.85, 31.39)	0.07
Kenya 2014 DHS	-	-	-	-	1.10 (0.82, 1.47)	0.54	1.06 (0.66, 1.68)	0.82
Kenya 2015 MIS	0.98 (0.60, 1.58)	0.92	0.89 (0.59, 1.33)	0.57	-	-	-	-
Lesotho 2009 DHS	-	-	-	-	1.32 (0.53, 3.30)	0.55	-	-

Table A. Association between finished house construction materials and malaria, diarrhoea and acute respiratory infection in children aged 0-5 years in sub-Saharan Africa (adjusted results)

Survey	Malaria positive by microscopy		Malaria positive by RDT		Diarrhoea in past two weeks		Cough with short and rapid breathing in past two weeks	
	**OR (95% CI)**	**p**	**OR (95% CI)**	**p**	**OR (95% CI)**	**p**	**OR (95% CI)**	**p**
Lesotho 2014 DHS	-	-	-	-	0.81 (0.51, 1.29)	0.38	0.77 (0.34, 1.72)	0.52
Liberia 2011 MIS	0.96 (0.74, 1.24)	0.76	0.99 (0.77, 1.26)	0.91	-	-	-	-
Liberia 2013 DHS	-	-	-	-	1.20 (0.80, 1.81)	0.37	1.71 (0.72, 4.06)	0.22
Liberia 2016 MIS	-	-	0.80 (0.62, 1.02)	0.08	-	-	-	-
Madagascar 2008 DHS	-	-	-	-	1.18 (0.71, 1.98)	0.52	-	-
Madagascar 2011 MIS	0.48 (0.20, 1.17)	0.10	0.88 (0.50, 1.53)	0.64	-	-	-	-
Madagascar 2013 MIS	0.77 (0.40, 1.45)	0.41	1.05 (0.61, 1.81)	0.85	-	-	-	-
Malawi 2010 DHS	-	-	-	-	1.09 (0.94, 1.25)	0.26	0.91 (0.74, 1.13)	0.39
Malawi 2012 MIS	0.52 (0.37, 0.75)	<0.001	0.56 (0.40, 0.78)	<0.001	-	-	-	-
Malawi 2014 MIS	0.94 (0.68, 1.29)	0.69	0.97 (0.71, 1.33)	0.86	-	-	-	-
Malawi 2015 DHS	-	-	-	-	0.95 (0.83, 1.09)	0.44	0.88 (0.73, 1.06)	0.17
Malawi 2017 MIS	0.85 (0.64, 1.14)	0.28	0.78 (0.60, 1.02)	0.07	-	-	-	-
Mali 2012 DHS	0.79 (0.62, 1.01)	0.07	0.95 (0.73, 1.23)	0.69	1.30 (0.84, 2.04)	0.24	-	-
Mali 2015 MIS	0.82 (0.69, 0.98)	0.03	0.92 (0.76, 1.11)	0.37	-	-	-	-
Mozambique 2011 DHS	0.84 (0.62, 1.15)	0.28	0.75 (0.55, 1.01)	0.06	0.99 (0.73, 1.35)	0.96	-	-
Mozambique 2015 AIS	-	-	1.04 (0.77, 1.40)	0.82	-	-	-	-
Namibia 2006 DHS	-	-	-	-	0.90 (0.48, 1.68)	0.74	1.00 (0.18, 5.61)	1.00
Namibia 2013 DHS	-	-	-	-	0.85 (0.60, 1.20)	0.36	1.15 (0.68, 1.94)	0.60
Niger 2012 DHS	-	-	-	-	1.02 (0.73, 1.42)	0.93	1.04 (0.54, 2.02)	0.90
Nigeria 2008 DHS	-	-	-	-	0.60 (0.17, 2.12)	0.43	-	-
Nigeria 2010 MIS	1.07 (0.86, 1.34)	0.55	0.85 (0.68, 1.08)	0.18	-	-	-	-
Nigeria 2013 DHS	-	-	-	-	0.68 (0.35, 1.32)	0.26	-	-
Nigeria 2015 MIS	0.93 (0.76, 1.15)	0.52	1.02 (0.84, 1.25)	0.81	-	-	-	-
Rwanda 2010 DHS	0.80 (0.41, 1.58)	0.53	0.79 (0.47, 1.34)	0.38	1.12 (0.89, 1.41)	0.33	0.80 (0.54, 1.18)	0.26
Rwanda 2015 DHS	0.72 (0.31, 1.66)	0.44	0.42 (0.26, 0.69)	<0.001	1.04 (0.83, 1.32)	0.73	0.92 (0.66, 1.29)	0.64
Rwanda 2017 MIS	0.71 (0.44, 1.14)	0.16	0.65 (0.43, 0.99)	0.04	-	-	-	-
Senegal 2008 MIS	0.95 (0.59, 1.54)	0.84	1.35 (0.93, 1.96)	0.12	-	-	-	-
Senegal 2010 DHS	1.42 (0.82, 2.46)	0.21	0.93 (0.52, 1.67)	0.82	1.07 (0.85, 1.35)	0.55	1.14 (0.66, 1.97)	0.64
Senegal 2012 DHS	0.67 (0.44, 1.03)	0.07	0.74 (0.50, 1.09)	0.13	1.03 (0.73, 1.45)	0.86	-	-
Senegal 2014 DHS	0.89 (0.52, 1.53)	0.68	0.88 (0.51, 1.52)	0.65	0.69 (0.50, 0.96)	0.03	-	-

Table A. Association between finished house construction materials and malaria, diarrhoea and acute respiratory infection in children aged 0-5 years in sub-Saharan Africa (adjusted results)

**Survey**	**Malaria positive by microscopy**		**Malaria positive by RDT**		**Diarrhoea in past two weeks**		**Cough with short and rapid breathing in past two weeks**	
	**OR (95% CI)**	**p**	**OR (95% CI)**	**p**	**OR (95% CI)**	**p**	**OR (95% CI)**	**p**
Senegal 2015 DHS	1.43 (0.50, 4.09)	0.50	1.02 (0.56, 1.83)	0.96	0.81 (0.58, 1.13)	0.21	-	-
Senegal 2016 DHS	1.36 (0.74, 2.50)	0.33	1.47 (0.87, 2.49)	0.15	1.42 (0.99, 2.04)	0.06	-	-
Sierra Leone 2008 DHS	-	-	-	-	0.64 (0.26, 1.55)	0.32	-	-
Sierra Leone 2013 DHS	-	-	-	-	0.97 (0.75, 1.25)	0.79	0.78 (0.48, 1.27)	0.32
Sierra Leone 2016 MIS	0.98 (0.86, 1.12)	0.79	0.91 (0.79, 1.05)	0.18	-	-	-	-
Tanzania 2004 DHS	-	-	-	-	1.20 (0.86, 1.67)	0.27	-	-
Tanzania 2010 DHS	-	-	-	-	1.07 (0.79, 1.45)	0.66	1.04 (0.59, 1.85)	0.88
Tanzania 2012 AIS	0.67 (0.47, 0.95)	0.03	0.70 (0.53, 0.92)	0.01	-	-	-	-
Tanzania 2017 MIS	-	-	0.87 (0.67, 1.12)	0.28	-	-	-	-
Togo 2013 DHS	1.13 (0.90, 1.40)	0.29	1.00 (0.80, 1.25)	0.99	1.25 (0.89, 1.76)	0.20	0.85 (0.46, 1.59)	0.62
Uganda 2006 DHS	-	-	-	-	1.04 (0.63, 1.73)	0.88	0.78 (0.33, 1.85)	0.57
Uganda 2009 MIS	0.72 (0.56, 0.91)	0.01	0.69 (0.55, 0.88)	0.002	-	-	-	-
Uganda 2014 MIS	0.89 (0.69, 1.15)	0.37	0.80 (0.63, 1.02)	0.07	-	-	-	-
Uganda 2016 DHS	-	-	0.62 (0.49, 0.78)	<0.001	1.23 (1.01, 1.51)	0.04	1.20 (0.94, 1.53)	0.13
Zambia 2007 DHS	-	-	-	-	1.54 (0.84, 2.82)	0.16	0.83 (0.21, 3.38)	0.80
Zambia 2013 DHS	-	-	-	-	1.06 (0.87, 1.29)	0.57	1.34 (0.91, 1.97)	0.14
Zimbabwe 2005 DHS	-	-	-	-	1.03 (0.71, 1.49)	0.89	0.98 (0.49, 1.95)	0.95
Zimbabwe 2010 DHS	-	-	-	-	0.94 (0.66, 1.36)	0.76	1.25 (0.68, 2.30)	0.48
Zimbabwe 2015 DHS	-	-	-	-	1.02 (0.73, 1.43)	0.90	1.22 (0.77, 1.93)	0.41

AIS: AIDS Indicator Survey; CI: confidence intervals; DHS: Demographic and Health Survey; DRC: Democratic Republic of the Congo; MIS: Malaria Indicator Survey; OR: Odds Ratio; RDT: rapid diagnostic test. All p-values are calculated using the likelihood ratio test.

Table B. Association between finished house construction materials, growth failure and anaemia in children aged 0-5 years in sub-Saharan Africa (adjusted results)

Survey	Low height-for-age		Low weight-for-height		Low weight-for-age		Any anaemia		Moderate to severe anaemia	
	OR (95% CI)	p	OR (95% CI)	p	OR (95% CI)	p	OR (95% CI)	p	OR (95% CI)	p
Angola 2011 MIS	-	-	-	-	-	-	-	-	-	-
Angola 2015 DHS	1.14 (0.77, 1.68)	0.51	1.30 (0.54, 3.14)	0.56	0.95 (0.61, 1.48)	0.83	-	-	-	-
Benin 2001 DHS	-	-	-	-	-	-	-	-	-	-
Benin 2006 DHS	0.84 (0.72, 0.97)	0.02	1.06 (0.79, 1.42)	0.70	0.78 (0.66, 0.93)	0.01	0.92 (0.63, 1.35)	0.66	0.89 (0.64, 1.24)	0.50
Benin 2012 DHS	0.84 (0.70, 1.01)	0.07	0.93 (0.73, 1.17)	0.52	0.88 (0.72, 1.08)	0.22	1.09 (0.73, 1.63)	0.67	1.51 (1.01, 2.24)	0.04
Burkina Faso 2010 DHS	1.04 (0.83, 1.29)	0.75	0.83 (0.63, 1.11)	0.22	1.01 (0.81, 1.26)	0.93	0.68 (0.49, 0.96)	0.03	0.89 (0.71, 1.12)	0.33
Burkina Faso 2014 MIS	-	-	-	-	-	-	-	-	-	-
Burundi 2010 DHS	0.78 (0.59, 1.04)	0.10	0.87 (0.45, 1.69)	0.68	0.70 (0.52, 0.96)	0.03	1.10 (0.81, 1.51)	0.53	1.06 (0.72, 1.56)	0.76
Burundi 2012 MIS	-	-	-	-	-	-	-	-	-	-
Burundi 2016 DHS	0.86 (0.68, 1.08)	0.19	1.40 (0.85, 2.31)	0.19	0.75 (0.59, 0.95)	0.01	0.75 (0.59, 0.97)	0.03	0.69 (0.53, 0.89)	0.004
Cameroon 2011 DHS	0.91 (0.65, 1.26)	0.56	0.97 (0.44, 2.16)	0.95	0.99 (0.63, 1.56)	0.96	1.27 (0.88, 1.83)	0.20	0.83 (0.57, 1.21)	0.34
Comoros 2012 DHS	0.65 (0.41, 1.01)	0.06	0.92 (0.49, 1.75)	0.80	0.81 (0.49, 1.33)	0.40	-	-	-	-
Congo 2005 DHS	0.78 (0.59, 1.04)	0.09	1.15 (0.73, 1.80)	0.55	0.94 (0.68, 1.30)	0.70	1.16 (0.79, 1.68)	0.45	0.82 (0.57, 1.17)	0.27
Congo 2011 DHS	0.83 (0.63, 1.08)	0.16	0.89 (0.55, 1.47)	0.66	0.88 (0.66, 1.17)	0.37	0.98 (0.76, 1.26)	0.86	1.20 (0.93, 1.55)	0.16
DRC 2013 DHS	0.94 (0.73, 1.20)	0.61	1.07 (0.65, 1.75)	0.79	0.94 (0.71, 1.23)	0.65	1.03 (0.79, 1.34)	0.82	1.05 (0.80, 1.36)	0.73
Cote d'Ivoire 2012 DHS	0.86 (0.54, 1.37)	0.52	0.99 (0.42, 2.28)	0.97	0.73 (0.45, 1.18)	0.20	0.99 (0.58, 1.67)	0.96	1.11 (0.72, 1.71)	0.64
Eswatini 2006 DHS	0.72 (0.47, 1.12)	0.15	1.75 (0.45, 6.81)	0.42	0.46 (0.22, 0.94)	0.03	1.16 (0.76, 1.77)	0.49	1.38 (0.82, 2.31)	0.23
Ethiopia 2016 DHS	-	-	-	-	-	-	-	-	-	-
Gabon 2012 DHS	0.99 (0.71, 1.39)	0.95	1.21 (0.51, 2.91)	0.66	1.17 (0.75, 1.83)	0.49	1.05 (0.76, 1.44)	0.78	0.88 (0.65, 1.19)	0.41
Gambia 2013 DHS	0.69 (0.44, 1.10)	0.12	0.80 (0.44, 1.49)	0.49	1.16 (0.74, 1.82)	0.51	0.75 (0.46, 1.23)	0.25	0.91 (0.61, 1.38)	0.67
Ghana 2008 DHS	0.90 (0.33, 2.44)	0.83	0.33 (0.07, 1.63)	0.17	0.56 (0.17, 1.84)	0.34	1.82 (0.34, 9.71)	0.48	0.63 (0.17, 2.37)	0.49
Ghana 2014 DHS	0.62 (0.29, 1.36)	0.23	1.26 (0.40, 3.92)	0.69	0.71 (0.35, 1.44)	0.34	1.11 (0.56, 2.20)	0.77	0.84 (0.46, 1.54)	0.58
Ghana 2016 MIS	-	-	-	-	-	-	-	-	-	-
Guinea 2012 DHS	1.37 (0.81, 2.32)	0.24	1.05 (0.51, 2.17)	0.89	1.52 (0.90, 2.55)	0.11	0.68 (0.39, 1.19)	0.18	0.99 (0.63, 1.56)	0.98
Kenya 2008 DHS	0.87 (0.61, 1.25)	0.44	0.71 (0.36, 1.40)	0.32	0.87 (0.58, 1.33)	0.53	-	-	-	-
Kenya 2014 DHS	0.96 (0.72, 1.28)	0.80	1.32 (0.71, 2.46)	0.38	0.92 (0.66, 1.27)	0.61	-	-	-	-
Kenya 2015 MIS	-	-	-	-	-	-	-	-	-	-
Lesotho 2009 DHS	-	-	-	-	-	-	-	-	-	-
Lesotho 2014 DHS	-	-	-	-	-	-	-	-	-	-
Liberia 2011 MIS	-	-	-	-	-	-	-	-	-	-

Table B. Association between finished house construction materials, growth failure and anaemia in children aged 0-5 years in sub-Saharan Africa (adjusted results)

Survey	Low height-for-age		Low weight-for-height		Low weight-for-age		Any anaemia		Moderate to severe anaemia	
	OR (95% CI)	p	OR (95% CI)	p	OR (95% CI)	p	OR (95% CI)	p	OR (95% CI)	p
Liberia 2013 DHS	0.51 (0.25, 1.06)	0.07	4.27 (0.99, 18.39)	0.05	0.87 (0.37, 2.05)	0.75	-	-	-	-
Liberia 2016 MIS	-	-	-	-	-	-	-	-	-	-
Madagascar 2008 DHS	0.75 (0.51, 1.13)	0.17	-	-	-	-	1.00 (0.53, 1.89)	0.99	1.60 (0.63, 4.11)	0.33
Madagascar 2011 MIS	-	-	-	-	-	-	-	-	-	-
Madagascar 2013 MIS	-	-	-	-	-	-	-	-	-	-
Malawi 2010 DHS	1.12 (0.86, 1.46)	0.39	0.36 (0.17, 0.76)	0.01	0.93 (0.65, 1.31)	0.66	0.93 (0.70, 1.24)	0.63	0.86 (0.64, 1.14)	0.29
Malawi 2012 MIS	-	-	-	-	-	-	-	-	-	-
Malawi 2014 MIS	-	-	-	-	-	-	-	-	-	-
Malawi 2015 DHS	0.67 (0.50, 0.90)	0.01	1.08 (0.50, 2.34)	0.85	1.04 (0.73, 1.47)	0.84	0.85 (0.62, 1.16)	0.30	1.02 (0.77, 1.36)	0.88
Malawi 2017 MIS	-	-	-	-	-	-	-	-	-	-
Mali 2012 DHS	1.27 (0.73, 2.21)	0.39	2.49 (0.90, 6.87)	0.08	1.24 (0.71, 2.17)	0.45	0.85 (0.47, 1.55)	0.60	0.80 (0.48, 1.33)	0.39
Mali 2015 MIS	-	-	-	-	-	-	-	-	-	-
Mozambique 2011 DHS	0.98 (0.78, 1.23)	0.87	0.90 (0.51, 1.58)	0.71	0.94 (0.69, 1.28)	0.71	0.84 (0.60, 1.16)	0.29	0.78 (0.55, 1.10)	0.15
Mozambique 2015 AIS	-	-	-	-	-	-	-	-	-	-
Namibia 2006 DHS	0.76 (0.50, 1.15)	0.19	1.26 (0.66, 2.39)	0.49	1.14 (0.75, 1.74)	0.53	-	-	-	-
Namibia 2013 DHS	0.71 (0.37, 1.33)	0.28	0.77 (0.29, 2.04)	0.60	0.44 (0.22, 0.88)	0.02	1.14 (0.66, 1.96)	0.63	1.06 (0.55, 2.05)	0.87
Niger 2012 DHS	0.81 (0.48, 1.38)	0.44	0.51 (0.26, 0.98)	0.04	0.85 (0.51, 1.43)	0.55	0.98 (0.53, 1.82)	0.96	1.30 (0.77, 2.18)	0.33
Nigeria 2008 DHS	0.69 (0.37, 1.30)	0.25	0.81 (0.30, 2.14)	0.67	0.58 (0.30, 1.14)	0.11	-	-	-	-
Nigeria 2010 MIS	-	-	-	-	-	-	-	-	-	-
Nigeria 2013 DHS	0.77 (0.45, 1.34)	0.36	0.97 (0.53, 1.77)	0.91	0.56 (0.34, 0.94)	0.03	-	-	-	-
Nigeria 2015 MIS	-	-	-	-	-	-	-	-	-	-
Rwanda 2010 DHS	0.65 (0.50, 0.84)	0.001	0.62 (0.27, 1.42)	0.26	0.61 (0.43, 0.87)	0.01	0.80 (0.61, 1.04)	0.10	0.67 (0.46, 0.98)	0.04
Rwanda 2015 DHS	0.88 (0.67, 1.14)	0.33	0.28 (0.09, 0.86)	0.03	0.65 (0.44, 0.96)	0.03	1.00 (0.77, 1.30)	1.00	0.78 (0.55, 1.13)	0.19
Rwanda 2017 MIS	-	-	-	-	-	-	-	-	-	-
Senegal 2008 MIS	-	-	-	-	-	-	-	-	-	-
Senegal 2010 DHS	1.07 (0.68, 1.69)	0.76	1.71 (0.83, 3.53)	0.15	0.97 (0.60, 1.55)	0.88	1.08 (0.65, 1.80)	0.77	1.07 (0.70, 1.64)	0.75
Senegal 2012 DHS	1.26 (0.89, 1.80)	0.19	0.89 (0.56, 1.41)	0.62	0.80 (0.58, 1.09)	0.16	0.91 (0.66, 1.26)	0.57	0.99 (0.74, 1.34)	0.97
Senegal 2014 DHS	0.88 (0.61, 1.26)	0.48	0.88 (0.49, 1.57)	0.66	0.91 (0.64, 1.28)	0.58	1.31 (0.94, 1.81)	0.11	1.27 (0.92, 1.75)	0.15
Senegal 2015 DHS	0.78 (0.53, 1.14)	0.20	0.86 (0.51, 1.43)	0.56	0.93 (0.66, 1.31)	0.66	1.33 (0.93, 1.90)	0.12	1.10 (0.80, 1.51)	0.56
Senegal 2016 DHS	1.16 (0.76, 1.77)	0.50	1.55 (0.90, 2.66)	0.11	1.17 (0.81, 1.68)	0.40	0.80 (0.55, 1.16)	0.24	0.75 (0.54, 1.03)	0.07

Table B. Association between finished house construction materials, growth failure and anaemia in children aged 0-5 years in sub-Saharan Africa (adjusted results)

Survey	Low height-for-age		Low weight-for-height		Low weight-for-age		Any anaemia		Moderate to severe anaemia	
	**OR (95% CI)**	**p**	**OR (95% CI)**	**p**	**OR (95% CI)**	**p**	**OR (95% CI)**	**p**	**OR (95% CI)**	**p**
Sierra Leone 2008 DHS	0.55 (0.29, 1.03)	0.06	0.68 (0.18, 2.59)	0.57	0.68 (0.34, 1.37)	0.28	0.91 (0.23, 3.60)	0.89	0.73 (0.21, 2.48)	0.61
Sierra Leone 2013 DHS	1.37 (0.99, 1.89)	0.06	0.66 (0.36, 1.19)	0.17	1.13 (0.79, 1.64)	0.50	0.96 (0.63, 1.46)	0.83	1.14 (0.82, 1.59)	0.42
Sierra Leone 2016 MIS	-	-	-	-	-	-	-	-	-	-
Tanzania 2004 DHS	0.74 (0.57, 0.96)	0.02	0.73 (0.36, 1.49)	0.39	0.74 (0.55, 1.00)	0.05	0.76 (0.59, 0.98)	0.03	0.68 (0.54, 0.87)	0.002
Tanzania 2010 DHS	1.02 (0.80, 1.30)	0.86	1.14 (0.68, 1.92)	0.62	1.09 (0.82, 1.44)	0.57	1.20 (0.95, 1.52)	0.13	1.20 (0.92, 1.55)	0.17
Tanzania 2012 AIS	-	-	-	-	-	-	-	-	-	-
Tanzania 2017 MIS	-	-	-	-	-	-	-	-	-	-
Togo 2013 DHS	0.54 (0.34, 0.84)	0.01	0.48 (0.20, 1.15)	0.10	0.67 (0.42, 1.07)	0.09	0.94 (0.59, 1.48)	0.78	0.81 (0.53, 1.25)	0.34
Uganda 2006 DHS	0.86 (0.39, 1.87)	0.70	0.39 (0.08, 1.91)	0.25	0.67 (0.25, 1.79)	0.42	0.16 (0.03, 0.86)	0.03	0.24 (0.07, 0.85)	0.03
Uganda 2009 MIS	-	-	-	-	-	-	-	-	-	-
Uganda 2014 MIS	-	-	-	-	-	-	-	-	-	-
Uganda 2016 DHS	0.69 (0.38, 1.26)	0.23	1.74 (0.58, 5.27)	0.33	0.77 (0.41, 1.45)	0.42	0.69 (0.44, 1.10)	0.12	0.65 (0.39, 1.07)	0.09
Zambia 2007 DHS	0.86 (0.62, 1.18)	0.36	1.17 (0.63, 2.19)	0.62	0.75 (0.51, 1.11)	0.15	-	-	-	-
Zambia 2013 DHS	0.97 (0.82, 1.14)	0.72	0.93 (0.67, 1.29)	0.66	0.80 (0.66, 0.98)	0.03	-	-	-	-
Zimbabwe 2005 DHS	0.86 (0.63, 1.16)	0.31	1.48 (0.82, 2.66)	0.19	1.05 (0.72, 1.54)	0.78	1.07 (0.79, 1.44)	0.66	1.21 (0.86, 1.69)	0.28
Zimbabwe 2010 DHS	0.85 (0.64, 1.13)	0.27	0.69 (0.32, 1.52)	0.36	0.87 (0.61, 1.25)	0.46	0.89 (0.66, 1.19)	0.42	0.97 (0.71, 1.33)	0.87
Zimbabwe 2015 DHS	0.96 (0.67, 1.36)	0.81	1.10 (0.57, 2.11)	0.77	0.75 (0.48, 1.17)	0.20	0.92 (0.65, 1.29)	0.61	1.47 (0.96, 2.26)	0.08

AIS: AIDS Indicator Survey; CI: confidence intervals; DHS: Demographic and Health Survey; DRC: Democratic Republic of the Congo; MIS: Malaria Indicator Survey; OR: Odds Ratio. All p-values are calculated using the likelihood ratio test.

Table C. Association between improved housing and malaria, diarrhoea and acute respiratory infection in children aged 0-5 years in sub-Saharan Africa (adjusted results)

Survey	Malaria positive by microscopy		Malaria positive by RDT		Diarrhoea in past two weeks		Cough with short and rapid breathing in past two weeks	
	**OR (95% CI)**	**p**	**OR (95% CI)**	**p**	**OR (95% CI)**	**p**	**OR (95% CI)**	**p**
Angola 2011 MIS	2.26 (0.82, 6.25)	0.12	0.17 (0.02, 1.40)	0.10	-	-	-	-
Angola 2015 DHS	-	-	0.72 (0.48, 1.09)	0.13	-	-	-	-
Benin 2001 DHS	-	-	-	-	-	-	-	-
Benin 2006 DHS	-	-	-	-	0.96 (0.64, 1.43)	0.85	1.45 (0.81, 2.58)	0.21
Benin 2012 DHS	0.70 (0.45, 1.08)	0.11	0.68 (0.42, 1.11)	0.12	1.03 (0.68, 1.56)	0.89	-	-
Burkina Faso 2010 DHS	0.90 (0.69, 1.17)	0.42	1.03 (0.78, 1.34)	0.85	1.02 (0.83, 1.27)	0.83	-	-
Burkina Faso 2014 MIS	0.71 (0.56, 0.92)	0.008	0.72 (0.57, 0.91)	0.007	-	-	-	-
Burundi 2010 DHS	-	-	-	-	0.93 (0.71, 1.23)	0.63	1.09 (0.65, 1.83)	0.74
Burundi 2012 MIS	0.79 (0.51, 1.24)	0.31	0.61 (0.40, 0.93)	0.02	-	-	-	-
Burundi 2016 DHS	1.09 (0.88, 1.35)	0.44	0.87 (0.71, 1.07)	0.18	1.03 (0.86, 1.23)	0.77	0.96 (0.77, 1.21)	0.75
Cameroon 2011 DHS	-	-	0.87 (0.71, 1.08)	0.21	0.70 (0.54, 0.92)	0.009	1.08 (0.76, 1.52)	0.67
Comoros 2012 DHS	-	-	-	-	1.24 (0.79, 1.94)	0.34	12.91 (2.14, 77.75)	0.005
Congo 2005 DHS	-	-	-	-	0.89 (0.55, 1.44)	0.63	0.87 (0.41, 1.83)	0.71
Congo 2011 DHS	-	-	-	-	0.81 (0.60, 1.08)	0.15	1.44 (0.87, 2.37)	0.15
DRC 2013 DHS	0.57 (0.34, 0.94)	0.03	0.70 (0.44, 1.11)	0.13	1.22 (0.92, 1.63)	0.17	0.92 (0.61, 1.40)	0.71
Cote d'Ivoire 2012 DHS	0.74 (0.50, 1.08)	0.11	0.77 (0.59, 1.00)	0.05	0.78 (0.60, 1.03)	0.08	0.58 (0.32, 1.04)	0.07
Eswatini 2006 DHS	-	-	-	-	1.13 (0.60, 2.12)	0.70	1.25 (0.50, 3.07)	0.63
Ethiopia 2016 DHS	-	-	-	-	0.56 (0.26, 1.22)	0.14	0.11 (0.01, 0.92)	0.04
Gabon 2012 DHS	-	-	-	-	0.78 (0.60, 1.00)	0.05	1.28 (0.90, 1.81)	0.17
Gambia 2013 DHS	-	-	0.39 (0.13, 1.18)	0.10	1.06 (0.85, 1.31)	0.62	-	-
Ghana 2008 DHS	-	-	-	-	0.70 (0.26, 1.91)	0.49	-	-
Ghana 2014 DHS	0.84 (0.59, 1.20)	0.34	1.15 (0.81, 1.62)	0.44	0.96 (0.62, 1.47)	0.83	0.66 (0.16, 2.65)	0.55
Ghana 2016 MIS	1.22 (0.84, 1.77)	0.30	0.78 (0.53, 1.14)	0.20	-	-	-	-
Guinea 2012 DHS	1.19 (0.88, 1.60)	0.26	0.90 (0.66, 1.22)	0.50	0.89 (0.66, 1.19)	0.43	0.80 (0.41, 1.53)	0.50
Kenya 2008 DHS	-	-	-	-	1.18 (0.60, 2.31)	0.64	0.66 (0.14, 3.05)	0.59
Kenya 2014 DHS	-	-	-	-	0.70 (0.48, 1.02)	0.07	2.18 (1.21, 3.92)	0.009
Kenya 2015 MIS	-	-	0.32 (0.12, 0.86)	0.02	-	-	-	-
Lesotho 2009 DHS	-	-	-	-	2.50 (0.63, 9.90)	0.19	-	-
Lesotho 2014 DHS	-	-	-	-	0.59 (0.35, 1.01)	0.05	0.83 (0.38, 1.83)	0.65
Liberia 2011 MIS	1.30 (0.78, 2.16)	0.31	0.83 (0.52, 1.34)	0.45	-	-	-	-
Liberia 2013 DHS	-	-	-	-	0.70 (0.38, 1.31)	0.26	0.34 (0.11, 1.07)	0.07
Liberia 2016 MIS	-	-	0.77 (0.52, 1.16)	0.21	-	-	-	-
Madagascar 2008 DHS	-	-	-	-	0.62 (0.24, 1.64)	0.34	-	-
Madagascar 2011 MIS	-	-	-	-	-	-	-	-

Table C. Association between improved housing and malaria, diarrhoea and acute respiratory infection in children aged 0-5 years in sub-Saharan Africa (adjusted results)

Survey	Malaria positive by microscopy		Malaria positive by RDT		Diarrhoea in past two weeks		Cough with short and rapid breathing in past two weeks	
	OR (95% CI)	p	OR (95% CI)	p	OR (95% CI)	p	OR (95% CI)	p
Madagascar 2013 MIS	6.95 (1.12, 43.27)	0.04	4.12 (0.75, 22.71)	0.10	-	-	-	-
Malawi 2010 DHS	-	-	-	-	1.06 (0.77, 1.47)	0.72	0.99 (0.59, 1.64)	0.95
Malawi 2012 MIS	0.30 (0.10, 0.92)	0.04	0.35 (0.15, 0.85)	0.02	-	-	-	-
Malawi 2014 MIS	1.01 (0.42, 2.39)	0.99	1.05 (0.46, 2.39)	0.91	-	-	-	-
Malawi 2015 DHS	-	-	-	-	0.91 (0.78, 1.07)	0.26	0.80 (0.65, 0.99)	0.04
Malawi 2017 MIS	0.50 (0.23, 1.08)	0.08	0.63 (0.34, 1.16)	0.14	-	-	-	-
Mali 2012 DHS	0.84 (0.61, 1.15)	0.27	0.99 (0.69, 1.41)	0.95	1.24 (0.88, 1.76)	0.22	-	-
Mali 2015 MIS	0.89 (0.71, 1.13)	0.34	0.73 (0.55, 0.97)	0.03	-	-	-	-
Mozambique 2011 DHS	0.44 (0.24, 0.84)	0.01	0.58 (0.32, 1.05)	0.07	1.09 (0.77, 1.55)	0.62	-	-
Mozambique 2015 AIS	-	-	0.92 (0.49, 1.73)	0.79	-	-	-	-
Namibia 2006 DHS	-	-	-	-	0.92 (0.53, 1.59)	0.76	0.94 (0.16, 5.36)	0.94
Namibia 2013 DHS	-	-	-	-	1.00 (0.70, 1.44)	0.98	0.58 (0.33, 1.02)	0.06
Niger 2012 DHS	-	-	-	-	1.02 (0.72, 1.44)	0.93	1.33 (0.71, 2.50)	0.38
Nigeria 2008 DHS	-	-	-	-	0.60 (0.27, 1.33)	0.21	-	-
Nigeria 2010 MIS	1.08 (0.82, 1.41)	0.59	0.73 (0.56, 0.96)	0.03	-	-	-	-
Nigeria 2013 DHS	-	-	-	-	0.94 (0.67, 1.32)	0.70	-	-
Nigeria 2015 MIS	0.98 (0.73, 1.31)	0.88	0.90 (0.71, 1.14)	0.38	-	-	-	-
Rwanda 2010 DHS	1.70 (0.68, 4.27)	0.26	1.46 (0.69, 3.09)	0.32	1.08 (0.83, 1.39)	0.57	0.85 (0.54, 1.34)	0.48
Rwanda 2015 DHS	0.63 (0.13, 2.94)	0.55	0.57 (0.27, 1.21)	0.14	0.84 (0.62, 1.13)	0.26	0.78 (0.52, 1.17)	0.23
Rwanda 2017 MIS	0.70 (0.39, 1.26)	0.23	0.58 (0.36, 0.93)	0.02	-	-	-	-
Senegal 2008 MIS	0.80 (0.32, 1.99)	0.63	1.05 (0.65, 1.68)	0.85	-	-	-	-
Senegal 2010 DHS	0.94 (0.45, 1.98)	0.87	0.88 (0.44, 1.79)	0.73	1.01 (0.83, 1.22)	0.96	1.02 (0.67, 1.55)	0.92
Senegal 2012 DHS	1.62 (0.80, 3.29)	0.18	0.98 (0.52, 1.85)	0.95	0.81 (0.59, 1.12)	0.21	-	-
Senegal 2014 DHS	0.44 (0.08, 2.60)	0.37	1.19 (0.29, 4.83)	0.81	0.78 (0.59, 1.04)	0.09	-	-
Senegal 2015 DHS	0.41 (0.08, 2.13)	0.29	1.07 (0.20, 5.79)	0.94	1.00 (0.77, 1.32)	0.97	-	-
Senegal 2016 DHS	1.23 (0.30, 4.94)	0.77	1.56 (0.40, 6.16)	0.52	0.88 (0.65, 1.20)	0.43	-	-
Sierra Leone 2008 DHS	-	-	-	-	0.86 (0.31, 2.37)	0.77	-	-
Sierra Leone 2013 DHS	-	-	-	-	0.96 (0.69, 1.34)	0.82	1.01 (0.59, 1.72)	0.97
Sierra Leone 2016 MIS	1.03 (0.85, 1.23)	0.78	1.02 (0.85, 1.23)	0.83	-	-	-	-
Tanzania 2004 DHS	-	-	-	-	-	-	-	-
Tanzania 2010 DHS	-	-	-	-	1.04 (0.69, 1.57)	0.85	0.74 (0.32, 1.74)	0.49
Tanzania 2012 AIS	0.70 (0.26, 1.90)	0.48	1.11 (0.56, 2.19)	0.76	-	-	-	-
Tanzania 2017 MIS	-	-	0.68 (0.39, 1.17)	0.16	-	-	-	-
Togo 2013 DHS	0.63 (0.42, 0.94)	0.02	0.67 (0.45, 1.01)	0.05	0.91 (0.62, 1.34)	0.63	0.88 (0.52, 1.50)	0.64

Table C. Association between improved housing and malaria, diarrhoea and acute respiratory infection in children aged 0-5 years in sub-Saharan Africa (adjusted results)

**Survey**	**Malaria positive by microscopy**		**Malaria positive by RDT**		**Diarrhoea in past two weeks**		**Cough with short and rapid breathing in past two weeks**	
	**OR (95% CI)**	**p**	**OR (95% CI)**	**p**	**OR (95% CI)**	**p**	**OR (95% CI)**	**p**
Uganda 2006 DHS	-	-	-	-	0.79 (0.32, 1.96)	0.61	1.01 (0.23, 4.46)	0.99
Uganda 2009 MIS	0.64 (0.41, 0.99)	0.04	0.64 (0.43, 0.96)	0.03	-	-	-	-
Uganda 2014 MIS	0.77 (0.43, 1.38)	0.38	0.87 (0.54, 1.41)	0.58	-	-	-	-
Uganda 2016 DHS	-	-	0.79 (0.53, 1.17)	0.24	0.80 (0.60, 1.05)	0.11	0.97 (0.69, 1.36)	0.87
Zambia 2007 DHS	-	-	-	-	1.34 (0.74, 2.43)	0.33	0.66 (0.13, 3.37)	0.62
Zambia 2013 DHS	-	-	-	-	0.80 (0.63, 1.02)	0.07	1.14 (0.73, 1.80)	0.56
Zimbabwe 2005 DHS	-	-	-	-	1.18 (0.82, 1.68)	0.37	0.52 (0.24, 1.12)	0.10
Zimbabwe 2010 DHS	-	-	-	-	0.91 (0.67, 1.24)	0.55	1.30 (0.66, 2.55)	0.45
Zimbabwe 2015 DHS	-	-	-	-	0.66 (0.50, 0.87)	0.003	0.85 (0.55, 1.29)	0.44

AIS: AIDS Indicator Survey; CI: confidence intervals; DHS: Demographic and Health Survey; DRC: Democratic Republic of the Congo; MIS: Malaria Indicator Survey; OR: Odds Ratio; RDT: rapid diagnostic test. All p-values are calculated using the likelihood ratio test.

Table D. Association between improved housing, growth failure and anaemia in children aged 0-5 years in sub-Saharan Africa (adjusted results)

Survey	Low height-for-age		Low weight-for-height		Low weight-for-age		Any anaemia		Moderate to severe anaemia	
	OR (95% CI)	p	OR (95% CI)	p	OR (95% CI)	p	OR (95% CI)	p	OR (95% CI)	p
Angola 2011 MIS	-	-	-	-	-	-	-	-	-	-
Angola 2015 DHS	0.80 (0.50, 1.28)	0.36	0.73 (0.27, 1.93)	0.52	0.84 (0.52, 1.37)	0.48	-	-	-	-
Benin 2001 DHS	-	-	-	-	-	-	-	-	-	-
Benin 2006 DHS	1.01 (0.78, 1.31)	0.96	0.60 (0.35, 1.02)	0.06	0.97 (0.71, 1.34)	0.86	0.77 (0.45, 1.34)	0.35	0.69 (0.41, 1.17)	0.17
Benin 2012 DHS	0.62 (0.47, 0.82)	<0.001	0.90 (0.62, 1.32)	0.59	0.70 (0.51, 0.97)	0.03	1.00 (0.58, 1.73)	1.00	1.28 (0.69, 2.40)	0.44
Burkina Faso 2010 DHS	0.82 (0.60, 1.11)	0.20	0.82 (0.56, 1.22)	0.33	0.76 (0.56, 1.02)	0.07	1.02 (0.71, 1.46)	0.91	0.97 (0.74, 1.28)	0.82
Burkina Faso 2014 MIS	-	-	-	-	-	-	-	-	-	-
Burundi 2010 DHS	0.69 (0.46, 1.05)	0.08	0.59 (0.23, 1.51)	0.27	0.77 (0.49, 1.20)	0.25	0.78 (0.51, 1.19)	0.25	0.71 (0.40, 1.24)	0.22
Burundi 2012 MIS	-	-	-	-	-	-	-	-	-	-
Burundi 2016 DHS	0.85 (0.64, 1.12)	0.24	1.27 (0.69, 2.33)	0.45	0.78 (0.59, 1.04)	0.09	0.72 (0.55, 0.95)	0.02	0.78 (0.58, 1.06)	0.11
Cameroon 2011 DHS	0.93 (0.67, 1.29)	0.66	1.02 (0.45, 2.28)	0.97	1.30 (0.84, 2.03)	0.24	0.89 (0.66, 1.20)	0.44	0.88 (0.63, 1.24)	0.47
Comoros 2012 DHS	0.90 (0.57, 1.44)	0.67	0.79 (0.42, 1.47)	0.46	0.96 (0.57, 1.60)	0.87	-	-	-	-
Congo 2005 DHS	0.71 (0.45, 1.11)	0.14	0.83 (0.40, 1.74)	0.62	0.87 (0.44, 1.71)	0.68	0.75 (0.48, 1.18)	0.22	0.61 (0.36, 1.05)	0.08
Congo 2011 DHS	0.85 (0.50, 1.43)	0.53	1.37 (0.69, 2.74)	0.37	0.92 (0.54, 1.59)	0.78	1.10 (0.76, 1.61)	0.61	0.72 (0.49, 1.05)	0.09
DRC 2013 DHS	0.78 (0.51, 1.20)	0.26	1.18 (0.56, 2.48)	0.65	0.58 (0.34, 1.00)	0.05	0.89 (0.62, 1.26)	0.50	0.79 (0.52, 1.18)	0.25
Cote d'Ivoire 2012 DHS	0.71 (0.45, 1.10)	0.12	0.56 (0.26, 1.20)	0.13	0.76 (0.48, 1.21)	0.25	1.10 (0.76, 1.61)	0.61	0.90 (0.64, 1.29)	0.57
Eswatini 2006 DHS	0.59 (0.33, 1.06)	0.08	1.52 (0.34, 6.84)	0.59	0.32 (0.09, 1.14)	0.08	0.54 (0.32, 0.90)	0.02	0.80 (0.41, 1.53)	0.49
Ethiopia 2016 DHS	-	-	-	-	-	-	-	-	-	-
Gabon 2012 DHS	0.63 (0.43, 0.93)	0.02	0.95 (0.50, 1.81)	0.88	0.69 (0.42, 1.15)	0.15	0.89 (0.68, 1.16)	0.38	0.84 (0.63, 1.12)	0.24
Gambia 2013 DHS	1.22 (0.79, 1.90)	0.37	0.66 (0.38, 1.15)	0.14	0.95 (0.62, 1.45)	0.80	0.58 (0.40, 0.85)	0.005	0.78 (0.55, 1.10)	0.16
Ghana 2008 DHS	1.28 (0.61, 2.72)	0.51	3.77 (1.03, 13.78)	0.04	1.62 (0.70, 3.75)	0.26	1.58 (0.67, 3.72)	0.29	1.28 (0.58, 2.83)	0.54
Ghana 2014 DHS	0.52 (0.24, 1.12)	0.10	0.86 (0.31, 2.36)	0.77	0.51 (0.26, 1.00)	0.05	0.94 (0.58, 1.51)	0.79	0.80 (0.48, 1.33)	0.39
Ghana 2016 MIS	-	-	-	-	-	-	-	-	-	-
Guinea 2012 DHS	1.11 (0.70, 1.76)	0.65	1.54 (0.82, 2.90)	0.18	0.98 (0.61, 1.58)	0.95	0.78 (0.53, 1.16)	0.22	0.82 (0.58, 1.17)	0.28
Kenya 2008 DHS	0.85 (0.55, 1.31)	0.46	1.15 (0.55, 2.42)	0.70	0.89 (0.53, 1.51)	0.67	-	-	-	-
Kenya 2014 DHS	0.71 (0.48, 1.06)	0.09	0.98 (0.44, 2.22)	0.97	0.93 (0.59, 1.46)	0.74	-	-	-	-
Kenya 2015 MIS	-	-	-	-	-	-	-	-	-	-
Lesotho 2009 DHS	-	-	-	-	-	-	-	-	-	-
Lesotho 2014 DHS	-	-	-	-	-	-	-	-	-	-
Liberia 2011 MIS	-	-	-	-	-	-	-	-	-	-
Liberia 2013 DHS	0.42 (0.12, 1.44)	0.17	0.47 (0.04, 4.93)	0.53	1.61 (0.43, 6.04)	0.48	-	-	-	-
Liberia 2016 MIS	-	-	-	-	-	-	-	-	-	-
Madagascar 2008 DHS	0.18 (0.06, 0.59)	0.005	-	-	-	-	1.76 (0.50, 6.24)	0.38	1.63 (0.37, 7.29)	0.52
Madagascar 2011 MIS	-	-	-	-	-	-	-	-	-	-
Madagascar 2013 MIS	-	-	-	-	-	-	-	-	-	-

Table D. Association between improved housing, growth failure and anaemia in children aged 0-5 years in sub-Saharan Africa (adjusted results)

Survey	Low height-for-age		Low weight-for-height		Low weight-for-age		Any anaemia		Moderate to severe anaemia	
	OR (95% CI)	p	OR (95% CI)	p	OR (95% CI)	p	OR (95% CI)	p	OR (95% CI)	p
Malawi 2010 DHS	0.77 (0.41, 1.46)	0.42	0.88 (0.18, 4.20)	0.87	1.34 (0.58, 3.07)	0.50	0.90 (0.48, 1.69)	0.73	1.15 (0.56, 2.33)	0.71
Malawi 2012 MIS	-	-	-	-	-	-	-	-	-	-
Malawi 2014 MIS	-	-	-	-	-	-	-	-	-	-
Malawi 2015 DHS	0.66 (0.46, 0.94)	0.02	1.56 (0.68, 3.60)	0.29	1.07 (0.70, 1.62)	0.75	1.47 (1.01, 2.14)	0.04	1.14 (0.83, 1.58)	0.42
Malawi 2017 MIS	-	-	-	-	-	-	-	-	-	-
Mali 2012 DHS	1.08 (0.68, 1.72)	0.73	0.57 (0.30, 1.09)	0.09	0.95 (0.61, 1.48)	0.82	0.84 (0.56, 1.27)	0.41	1.00 (0.68, 1.48)	0.99
Mali 2015 MIS	-	-	-	-	-	-	-	-	-	-
Mozambique 2011 DHS	1.06 (0.80, 1.41)	0.68	1.13 (0.60, 2.14)	0.71	1.01 (0.68, 1.49)	0.97	0.86 (0.60, 1.24)	0.42	1.04 (0.69, 1.56)	0.86
Mozambique 2015 AIS	-	-	-	-	-	-	-	-	-	-
Namibia 2006 DHS	0.64 (0.43, 0.97)	0.03	0.90 (0.45, 1.83)	0.78	0.68 (0.43, 1.06)	0.09	-	-	-	-
Namibia 2013 DHS	0.42 (0.19, 0.92)	0.03	0.90 (0.37, 2.22)	0.82	0.86 (0.43, 1.69)	0.65	0.97 (0.56, 1.66)	0.90	1.46 (0.75, 2.85)	0.26
Niger 2012 DHS	1.03 (0.57, 1.86)	0.93	1.72 (0.82, 3.61)	0.15	0.99 (0.56, 1.73)	0.96	1.10 (0.59, 2.02)	0.77	1.19 (0.69, 2.07)	0.53
Nigeria 2008 DHS	1.14 (0.86, 1.51)	0.37	0.99 (0.64, 1.53)	0.96	0.92 (0.65, 1.32)	0.66	-	-	-	-
Nigeria 2010 MIS	-	-	-	-	-	-	-	-	-	-
Nigeria 2013 DHS	0.92 (0.69, 1.22)	0.55	0.68 (0.51, 0.90)	0.007	0.85 (0.65, 1.11)	0.23	-	-	-	-
Nigeria 2015 MIS	-	-	-	-	-	-	-	-	-	-
Rwanda 2010 DHS	0.59 (0.43, 0.81)	<0.001	1.99 (0.82, 4.79)	0.13	0.54 (0.35, 0.83)	0.005	0.95 (0.70, 1.29)	0.73	0.84 (0.54, 1.30)	0.43
Rwanda 2015 DHS	0.84 (0.60, 1.18)	0.31	0.49 (0.14, 1.76)	0.28	0.78 (0.46, 1.34)	0.37	0.66 (0.48, 0.90)	0.01	0.74 (0.46, 1.19)	0.22
Rwanda 2017 MIS	-	-	-	-	-	-	-	-	-	-
Senegal 2008 MIS	-	-	-	-	-	-	-	-	-	-
Senegal 2010 DHS	1.22 (0.80, 1.84)	0.36	0.86 (0.51, 1.45)	0.57	1.04 (0.69, 1.54)	0.86	0.69 (0.48, 1.01)	0.05	0.79 (0.56, 1.10)	0.16
Senegal 2012 DHS	0.94 (0.65, 1.37)	0.75	0.85 (0.55, 1.33)	0.48	0.94 (0.69, 1.28)	0.71	0.80 (0.62, 1.04)	0.10	0.95 (0.74, 1.21)	0.66
Senegal 2014 DHS	0.63 (0.42, 0.95)	0.03	1.09 (0.67, 1.79)	0.72	0.91 (0.65, 1.27)	0.57	0.87 (0.67, 1.13)	0.30	0.75 (0.56, 1.00)	0.05
Senegal 2015 DHS	0.92 (0.66, 1.27)	0.61	1.01 (0.67, 1.53)	0.94	0.84 (0.63, 1.11)	0.22	0.91 (0.72, 1.16)	0.46	0.90 (0.70, 1.15)	0.40
Senegal 2016 DHS	0.80 (0.53, 1.22)	0.30	0.66 (0.41, 1.08)	0.10	0.67 (0.48, 0.94)	0.02	0.83 (0.64, 1.09)	0.18	0.82 (0.62, 1.08)	0.15
Sierra Leone 2008 DHS	0.40 (0.18, 0.92)	0.03	0.84 (0.22, 3.20)	0.80	0.53 (0.20, 1.39)	0.20	2.43 (0.56, 10.50)	0.24	1.49 (0.36, 6.17)	0.58
Sierra Leone 2013 DHS	0.95 (0.63, 1.45)	0.82	1.02 (0.49, 2.11)	0.96	1.26 (0.77, 2.06)	0.37	1.01 (0.64, 1.57)	0.98	1.12 (0.75, 1.66)	0.58
Sierra Leone 2016 MIS	-	-	-	-	-	-	-	-	-	-
Tanzania 2004 DHS	-	-	-	-	-	-	-	-	-	-
Tanzania 2010 DHS	0.77 (0.52, 1.13)	0.18	0.75 (0.40, 1.38)	0.36	0.79 (0.51, 1.22)	0.28	0.90 (0.66, 1.22)	0.49	0.92 (0.65, 1.30)	0.63
Tanzania 2012 AIS	-	-	-	-	-	-	-	-	-	-
Tanzania 2017 MIS	-	-	-	-	-	-	-	-	-	-
Togo 2013 DHS	0.85 (0.49, 1.48)	0.57	1.66 (0.72, 3.81)	0.23	0.70 (0.41, 1.21)	0.21	0.73 (0.48, 1.11)	0.15	0.64 (0.42, 0.97)	0.04
Uganda 2006 DHS	0.80 (0.23, 2.76)	0.72	0.29 (0.02, 4.74)	0.39	1.02 (0.19, 5.46)	0.98	0.16 (0.03, 0.88)	0.04	0.29 (0.05, 1.68)	0.17
Uganda 2009 MIS	-	-	-	-	-	-	-	-	-	-

Table D. Association between improved housing, growth failure and anaemia in children aged 0-5 years in sub-Saharan Africa (adjusted results)

Survey	Low height-for-age		Low weight-for-height		Low weight-for-age		Any anaemia		Moderate to severe anaemia	
	**OR (95% CI)**	**p**	**OR (95% CI)**	**p**	**OR (95% CI)**	**p**	**OR (95% CI)**	**p**	**OR (95% CI)**	**p**
Uganda 2014 MIS	-	-	-	-	-	-	-	-	-	-
Uganda 2016 DHS	0.47 (0.18, 1.21)	0.12	5.01 (0.75, 33.59)	0.10	0.47 (0.17, 1.28)	0.14	1.17 (0.65, 2.10)	0.61	1.25 (0.67, 2.35)	0.49
Zambia 2007 DHS	0.80 (0.54, 1.20)	0.28	1.51 (0.69, 3.29)	0.30	0.95 (0.57, 1.58)	0.84	-	-	-	-
Zambia 2013 DHS	0.96 (0.78, 1.17)	0.67	0.86 (0.58, 1.27)	0.45	0.85 (0.66, 1.09)	0.20	-	-	-	-
Zimbabwe 2005 DHS	1.00 (0.75, 1.32)	0.99	0.91 (0.53, 1.57)	0.73	0.91 (0.62, 1.32)	0.61	0.83 (0.63, 1.11)	0.21	0.86 (0.63, 1.17)	0.33
Zimbabwe 2010 DHS	0.73 (0.55, 0.95)	0.02	1.30 (0.65, 2.60)	0.46	0.83 (0.58, 1.19)	0.31	0.83 (0.63, 1.09)	0.18	0.98 (0.73, 1.31)	0.87
Zimbabwe 2015 DHS	0.84 (0.62, 1.13)	0.24	0.54 (0.27, 1.09)	0.09	0.68 (0.45, 1.04)	0.08	1.01 (0.78, 1.30)	0.96	1.20 (0.88, 1.64)	0.25

AIS: AIDS Indicator Survey; CI: confidence intervals; DHS: Demographic and Health Survey; DRC: Democratic Republic of the Congo; MIS: Malaria Indicator Survey; OR: Odds Ratio. All p-values are calculated using the likelihood ratio test.

Table E. Association between ITN use, improved drinking water source and improved sanitation facility and child health outcomes in children aged 0-5 years in sub-Saharan Africa (adjusted results)

Survey	ITN use the previous night				Improved drinking water source		Improved sanitation facility	
	Malaria positive by microscopy		Malaria positive by RDT		Diarrhoea in past two weeks		Diarrhoea in past two weeks	
	OR (95% CI)	p	OR (95% CI)	p	OR (95% CI)	p	OR (95% CI)	p
Angola 2011 MIS	0.62 (0.41, 0.95)	0.03	0.85 (0.58, 1.25)	0.40	-	-	-	-
Angola 2015 DHS	-	-	0.85 (0.68, 1.07)	0.17	-	-	-	-
Benin 2001 DHS	-	-	-	-	1.31 (0.88, 1.95)	0.18	1.26 (0.70, 2.26)	0.45
Benin 2006 DHS	-	-	-	-	0.75 (0.58, 0.97)	0.03	1.07 (0.81, 1.41)	0.63
Benin 2012 DHS	1.08 (0.89, 1.31)	0.45	0.97 (0.79, 1.20)	0.78	0.82 (0.59, 1.13)	0.22	0.90 (0.65, 1.24)	0.52
Burkina Faso 2010 DHS	0.94 (0.81, 1.09)	0.40	1.04 (0.89, 1.22)	0.60	0.96 (0.79, 1.16)	0.68	1.09 (0.90, 1.33)	0.35
Burkina Faso 2014 MIS	0.90 (0.78, 1.04)	0.15	0.86 (0.74, 1.01)	0.06	-	-	-	-
Burundi 2010 DHS	-	-	-	-	0.68 (0.55, 0.86)	0.001	0.89 (0.71, 1.11)	0.29
Burundi 2012 MIS	0.79 (0.61, 1.02)	0.07	0.71 (0.55, 0.91)	0.01	-	-	-	-
Burundi 2016 DHS	0.69 (0.59, 0.80)	<0.001	0.67 (0.58, 0.78)	<0.001	0.99 (0.80, 1.22)	0.90	0.99 (0.85, 1.14)	0.85
Cameroon 2011 DHS	-	-	0.82 (0.70, 0.97)	0.02	0.77 (0.57, 1.05)	0.10	0.96 (0.72, 1.26)	0.75
Comoros 2012 DHS	-	-	-	-	1.06 (0.59, 1.91)	0.85	1.08 (0.71, 1.64)	0.73
Congo 2005 DHS	-	-	-	-	1.13 (0.76, 1.66)	0.55	0.82 (0.59, 1.13)	0.22
Congo 2011 DHS	-	-	-	-	0.93 (0.74, 1.17)	0.54	0.71 (0.58, 0.88)	0.002
DRC 2013 DHS	0.89 (0.78, 1.02)	0.09	0.86 (0.76, 0.98)	0.03	0.86 (0.70, 1.04)	0.12	1.04 (0.87, 1.24)	0.68
Cote d'Ivoire 2012 DHS	0.85 (0.69, 1.04)	0.12	0.73 (0.62, 0.86)	<0.001	1.38 (1.01, 1.88)	0.04	1.09 (0.85, 1.40)	0.49
Eswatini 2006 DHS	-	-	-	-	0.99 (0.64, 1.53)	0.96	0.79 (0.50, 1.25)	0.32
Ethiopia 2016 DHS	-	-	-	-	0.89 (0.43, 1.83)	0.75	0.91 (0.51, 1.64)	0.75
Gabon 2012 DHS	-	-	-	-	0.81 (0.59, 1.12)	0.20	1.04 (0.82, 1.31)	0.75
Gambia 2013 DHS	0.59 (0.15, 2.30)	0.44	0.56 (0.28, 1.14)	0.11	1.13 (0.79, 1.60)	0.51	1.27 (0.99, 1.64)	0.06
Ghana 2008 DHS	-	-	-	-	0.18 (0.04, 0.72)	0.02	0.46 (0.14, 1.48)	0.19
Ghana 2014 DHS	0.93 (0.75, 1.15)	0.48	0.96 (0.78, 1.20)	0.74	1.46 (1.00, 2.14)	0.05	0.77 (0.54, 1.11)	0.17
Ghana 2016 MIS	0.75 (0.61, 0.93)	0.01	0.95 (0.78, 1.17)	0.66	-	-	-	-
Guinea 2012 DHS	0.98 (0.79, 1.21)	0.86	1.04 (0.83, 1.30)	0.73	0.84 (0.55, 1.28)	0.41	1.05 (0.75, 1.47)	0.78
Kenya 2008 DHS	-	-	-	-	1.01 (0.59, 1.72)	0.97	0.86 (0.52, 1.43)	0.57
Kenya 2014 DHS	-	-	-	-	0.97 (0.74, 1.28)	0.85	0.70 (0.54, 0.89)	0.004
Kenya 2015 MIS	0.45 (0.31, 0.65)	<0.001	0.62 (0.45, 0.85)	0.003	-	-	-	-
Lesotho 2009 DHS	-	-	-	-	0.59 (0.20, 1.75)	0.34	1.20 (0.42, 3.39)	0.74
Lesotho 2014 DHS	-	-	-	-	0.85 (0.51, 1.43)	0.55	0.67 (0.42, 1.06)	0.09
Liberia 2011 MIS	0.91 (0.76, 1.10)	0.34	0.99 (0.83, 1.18)	0.93	-	-	-	-

Table E. Association between ITN use, improved drinking water source and improved sanitation facility and child health outcomes in children aged 0-5 years in sub-Saharan Africa (adjusted results)

Survey	ITN use the previous night				Improved drinking water source		Improved sanitation facility	
	Malaria positive by microscopy		Malaria positive by RDT		Diarrhoea in past two weeks		Diarrhoea in past two weeks	
	OR (95% CI)	p	OR (95% CI)	p	OR (95% CI)	p	OR (95% CI)	p
Liberia 2013 DHS	-	-	-	-	1.23 (0.72, 2.11)	0.45	0.77 (0.53, 1.13)	0.19
Liberia 2016 MIS	-	-	0.85 (0.70, 1.03)	0.09	-	-	-	-
Madagascar 2008 DHS	-	-	-	-	0.96 (0.57, 1.62)	0.87	1.08 (0.64, 1.81)	0.78
Madagascar 2011 MIS	0.60 (0.39, 0.92)	0.02	0.71 (0.50, 1.02)	0.06	-	-	-	-
Madagascar 2013 MIS	0.69 (0.51, 0.94)	0.02	0.73 (0.55, 0.97)	0.03	-	-	-	-
Malawi 2010 DHS	-	-	-	-	0.92 (0.78, 1.08)	0.32	0.78 (0.64, 0.96)	0.02
Malawi 2012 MIS	0.63 (0.48, 0.81)	<0.001	0.71 (0.55, 0.93)	0.01	-	-	-	-
Malawi 2014 MIS	0.69 (0.52, 0.93)	0.01	0.71 (0.53, 0.96)	0.02	-	-	-	-
Malawi 2015 DHS	-	-	-	-	1.08 (0.88, 1.32)	0.46	0.89 (0.76, 1.03)	0.12
Malawi 2017 MIS	0.61 (0.47, 0.79)	<0.001	0.70 (0.55, 0.89)	0.004	-	-	-	-
Mali 2012 DHS	0.99 (0.84, 1.16)	0.88	1.00 (0.86, 1.18)	0.96	1.17 (0.66, 2.07)	0.60	1.13 (0.74, 1.72)	0.56
Mali 2015 MIS	0.86 (0.76, 0.99)	0.03	0.83 (0.72, 0.96)	0.01	-	-	-	-
Mozambique 2011 DHS	0.99 (0.82, 1.19)	0.91	0.94 (0.78, 1.13)	0.48	1.01 (0.72, 1.41)	0.97	1.05 (0.83, 1.33)	0.70
Mozambique 2015 AIS	-	-	0.96 (0.80, 1.16)	0.66	-	-	-	-
Namibia 2006 DHS	-	-	-	-	0.72 (0.38, 1.37)	0.32	0.73 (0.41, 1.30)	0.28
Namibia 2013 DHS	-	-	-	-	1.12 (0.74, 1.70)	0.59	1.17 (0.81, 1.71)	0.40
Niger 2012 DHS	-	-	-	-	0.91 (0.58, 1.41)	0.66	0.81 (0.58, 1.14)	0.23
Nigeria 2008 DHS	-	-	-	-	1.16 (0.52, 2.61)	0.72	0.52 (0.21, 1.25)	0.14
Nigeria 2010 MIS	0.89 (0.74, 1.06)	0.20	0.93 (0.78, 1.12)	0.45	-	-	-	-
Nigeria 2013 DHS	-	-	-	-	1.12 (0.79, 1.61)	0.52	0.77 (0.51, 1.17)	0.22
Nigeria 2015 MIS	0.98 (0.84, 1.16)	0.84	1.06 (0.91, 1.22)	0.46	-	-	-	-
Rwanda 2010 DHS	0.68 (0.38, 1.21)	0.19	0.51 (0.33, 0.80)	0.003	0.75 (0.59, 0.96)	0.02	1.00 (0.80, 1.25)	1.00
Rwanda 2015 DHS	0.52 (0.32, 0.84)	0.01	0.63 (0.46, 0.86)	0.004	1.06 (0.85, 1.33)	0.62	1.04 (0.85, 1.26)	0.71
Rwanda 2017 MIS	0.53 (0.37, 0.77)	<0.001	0.51 (0.37, 0.71)	<0.001	-	-	-	-
Senegal 2008 MIS	1.21 (0.85, 1.70)	0.29	1.22 (0.93, 1.59)	0.15	-	-	-	-
Senegal 2010 DHS	1.15 (0.78, 1.70)	0.46	1.11 (0.74, 1.68)	0.61	0.97 (0.75, 1.24)	0.79	0.90 (0.73, 1.13)	0.37
Senegal 2012 DHS	0.68 (0.49, 0.94)	0.02	0.70 (0.52, 0.95)	0.02	0.81 (0.58, 1.14)	0.23	1.11 (0.81, 1.51)	0.53
Senegal 2014 DHS	0.87 (0.49, 1.54)	0.63	0.95 (0.53, 1.68)	0.85	1.37 (0.98, 1.93)	0.07	1.19 (0.87, 1.62)	0.28
Senegal 2015 DHS	1.38 (0.54, 3.52)	0.50	0.71 (0.37, 1.35)	0.30	1.11 (0.83, 1.50)	0.48	1.00 (0.74, 1.35)	0.99
Senegal 2016 DHS	1.28 (0.76, 2.14)	0.35	1.19 (0.73, 1.94)	0.48	0.96 (0.66, 1.39)	0.83	1.15 (0.84, 1.59)	0.38

Table E. Association between ITN use, improved drinking water source and improved sanitation facility and child health outcomes in children aged 0-5 years in sub-Saharan Africa (adjusted results)

Survey	ITN use the previous night				Improved drinking water source		Improved sanitation facility	
	Malaria positive by microscopy		Malaria positive by RDT		Diarrhoea in past two weeks		Diarrhoea in past two weeks	
	OR (95% CI)	p	OR (95% CI)	p	OR (95% CI)	p	OR (95% CI)	p
Sierra Leone 2008 DHS	-	-	-	-	3.61 (1.23, 10.57)	0.02	0.84 (0.39, 1.82)	0.66
Sierra Leone 2013 DHS	-	-	-	-	1.10 (0.81, 1.49)	0.55	1.14 (0.89, 1.45)	0.29
Sierra Leone 2016 MIS	0.87 (0.78, 0.97)	0.01	0.92 (0.82, 1.04)	0.17	-	-	-	-
Tanzania 2004 DHS	-	-	-	-	1.12 (0.71, 1.76)	0.64	1.35 (0.79, 2.31)	0.28
Tanzania 2010 DHS	-	-	-	-	0.93 (0.66, 1.30)	0.66	1.02 (0.75, 1.38)	0.89
Tanzania 2012 AIS	0.81 (0.61, 1.07)	0.14	0.86 (0.69, 1.07)	0.18	-	-	-	-
Tanzania 2017 MIS	-	-	0.79 (0.64, 0.98)	0.04	-	-	-	-
Togo 2013 DHS	0.78 (0.66, 0.93)	0.01	0.75 (0.63, 0.90)	0.002	0.97 (0.72, 1.29)	0.83	0.85 (0.61, 1.20)	0.36
Uganda 2006 DHS	-	-	-	-	1.36 (0.77, 2.39)	0.28	1.19 (0.78, 1.82)	0.42
Uganda 2009 MIS	0.67 (0.55, 0.80)	<0.001	0.67 (0.56, 0.81)	<0.001	-	-	-	-
Uganda 2014 MIS	1.03 (0.83, 1.27)	0.81	0.93 (0.76, 1.14)	0.48	-	-	-	-
Uganda 2016 DHS	-	-	0.69 (0.58, 0.81)	<0.001	0.93 (0.73, 1.18)	0.54	1.00 (0.83, 1.21)	0.98
Zambia 2007 DHS	-	-	-	-	0.97 (0.57, 1.64)	0.91	1.11 (0.67, 1.83)	0.68
Zambia 2013 DHS	-	-	-	-	1.09 (0.89, 1.32)	0.41	1.00 (0.84, 1.19)	0.99
Zimbabwe 2005 DHS	-	-	-	-	1.47 (1.00, 2.17)	0.05	0.98 (0.69, 1.40)	0.93
Zimbabwe 2010 DHS	-	-	-	-	0.86 (0.59, 1.25)	0.43	0.84 (0.61, 1.17)	0.31
Zimbabwe 2015 DHS	-	-	-	-	0.69 (0.49, 0.98)	0.04	1.17 (0.87, 1.58)	0.30

AIS: AIDS Indicator Survey; CI: confidence intervals; DHS: Demographic and Health Survey; DRC: Democratic Republic of the Congo; ITN: insecticide-treated bednet; MIS: Malaria Indicator Survey; OR: Odds Ratio; RDT: rapid diagnostic test. All p-values are calculated using the likelihood ratio test.

Table F. Association between finished house construction materials and malaria, diarrhoea and acute respiratory infection in children aged 0-5 years in sub-Saharan Africa (crude results)

Survey	Malaria positive by microscopy		Malaria positive by RDT		Diarrhoea in past two weeks		Cough with short and rapid breathing in past two weeks	
	OR (95% CI)	p	OR (95% CI)	p	OR (95% CI)	p	OR (95% CI)	p
Angola 2011 MIS	0.98 (0.53, 1.81)	0.95	0.61 (0.33, 1.12)	0.11	-	-	-	-
Angola 2015 DHS	-	-	0.79 (0.61, 1.03)	0.08	-	-	-	-
Benin 2001 DHS	-	-	-	-	1.12 (0.80, 1.57)	0.51	-	-
Benin 2006 DHS	-	-	-	-	1.00 (0.79, 1.26)	1.00	0.99 (0.69, 1.43)	0.96
Benin 2012 DHS	0.77 (0.62, 0.95)	0.02	0.77 (0.62, 0.97)	0.02	1.06 (0.80, 1.39)	0.70	-	-
Burkina Faso 2010 DHS	0.94 (0.80, 1.11)	0.49	0.86 (0.71, 1.05)	0.14	0.91 (0.78, 1.07)	0.27	-	-
Burkina Faso 2014 MIS	0.87 (0.76, 1.00)	0.06	0.94 (0.80, 1.09)	0.38	-	-	-	-
Burundi 2010 DHS	-	-	-	-	0.80 (0.66, 0.96)	0.02	1.02 (0.70, 1.49)	0.91
Burundi 2012 MIS	0.62 (0.45, 0.85)	0.003	0.51 (0.37, 0.69)	<0.001	-	-	-	-
Burundi 2016 DHS	0.64 (0.55, 0.74)	<0.001	0.61 (0.53, 0.71)	<0.001	0.88 (0.76, 1.01)	0.07	0.93 (0.77, 1.11)	0.41
Cameroon 2011 DHS	-	-	0.78 (0.65, 0.93)	0.006	0.75 (0.57, 0.98)	0.04	1.43 (0.96, 2.13)	0.08
Comoros 2012 DHS	-	-	-	-	1.66 (1.06, 2.60)	0.03	2.35 (0.77, 7.14)	0.13
Congo 2005 DHS	-	-	-	-	0.77 (0.56, 1.06)	0.11	1.14 (0.63, 2.07)	0.67
Congo 2011 DHS	-	-	-	-	1.05 (0.86, 1.27)	0.66	0.84 (0.59, 1.19)	0.33
DRC 2013 DHS	0.71 (0.56, 0.91)	0.006	0.72 (0.57, 0.91)	0.006	1.10 (0.92, 1.33)	0.30	0.84 (0.63, 1.11)	0.22
Cote d'Ivoire 2012 DHS	0.77 (0.61, 0.97)	0.03	0.84 (0.69, 1.03)	0.10	0.78 (0.59, 1.04)	0.09	0.66 (0.34, 1.28)	0.22
Eswatini 2006 DHS	-	-	-	-	0.63 (0.41, 0.96)	0.03	0.76 (0.40, 1.48)	0.42
Ethiopia 2016 DHS	-	-	-	-	0.88 (0.49, 1.58)	0.67	1.47 (0.64, 3.39)	0.36
Gabon 2012 DHS	-	-	-	-	1.06 (0.83, 1.36)	0.63	0.88 (0.61, 1.25)	0.47
Gambia 2013 DHS	1.25 (0.29, 5.34)	0.77	0.55 (0.24, 1.30)	0.17	1.07 (0.81, 1.40)	0.65	-	-
Ghana 2008 DHS	-	-	-	-	0.39 (0.11, 1.37)	0.14	-	-
Ghana 2014 DHS	0.70 (0.52, 0.95)	0.02	0.73 (0.53, 1.01)	0.06	1.07 (0.66, 1.72)	0.79	0.91 (0.24, 3.46)	0.89
Ghana 2016 MIS	0.88 (0.63, 1.23)	0.46	0.80 (0.58, 1.10)	0.17	-	-	-	-
Guinea 2012 DHS	0.95 (0.76, 1.20)	0.69	0.89 (0.70, 1.13)	0.36	1.02 (0.70, 1.49)	0.92	0.87 (0.35, 2.16)	0.76
Kenya 2008 DHS	-	-	-	-	0.76 (0.45, 1.28)	0.30	3.65 (0.93, 14.31)	0.06
Kenya 2014 DHS	-	-	-	-	0.89 (0.68, 1.15)	0.36	0.97 (0.64, 1.46)	0.88
Kenya 2015 MIS	0.86 (0.55, 1.34)	0.50	0.73 (0.50, 1.07)	0.10	-	-	-	-
Lesotho 2009 DHS	-	-	-	-	1.23 (0.57, 2.64)	0.59	-	-
Lesotho 2014 DHS	-	-	-	-	0.71 (0.47, 1.06)	0.09	0.73 (0.37, 1.43)	0.36
Liberia 2011 MIS	0.88 (0.69, 1.13)	0.31	0.89 (0.71, 1.12)	0.33	-	-	-	-
Liberia 2013 DHS	-	-	-	-	1.09 (0.74, 1.59)	0.66	1.56 (0.75, 3.21)	0.23
Liberia 2016 MIS	-	-	0.81 (0.64, 1.02)	0.07	-	-	-	-
Madagascar 2008 DHS	-	-	-	-	1.24 (0.79, 1.96)	0.35	-	-
Madagascar 2011 MIS	0.44 (0.19, 1.04)	0.06	0.76 (0.44, 1.29)	0.31	-	-	-	-

Table F. Association between finished house construction materials and malaria, diarrhoea and acute respiratory infection in children aged 0-5 years in sub-Saharan Africa (crude results)

Survey	Malaria positive by microscopy		Malaria positive by RDT		Diarrhoea in past two weeks		Cough with short and rapid breathing in past two weeks	
	OR (95% CI)	p	OR (95% CI)	p	OR (95% CI)	p	OR (95% CI)	p
Madagascar 2013 MIS	0.60 (0.32, 1.12)	0.11	0.88 (0.52, 1.49)	0.64	-	-	-	-
Malawi 2010 DHS	-	-	-	-	0.94 (0.83, 1.07)	0.35	0.85 (0.70, 1.04)	0.11
Malawi 2012 MIS	0.49 (0.35, 0.68)	<0.001	0.48 (0.36, 0.65)	<0.001	-	-	-	-
Malawi 2014 MIS	0.89 (0.66, 1.20)	0.44	0.85 (0.63, 1.13)	0.26	-	-	-	-
Malawi 2015 DHS	-	-	-	-	0.90 (0.79, 1.01)	0.08	0.91 (0.77, 1.08)	0.29
Malawi 2017 MIS	0.65 (0.50, 0.85)	0.002	0.61 (0.48, 0.77)	<0.001	-	-	-	-
Mali 2012 DHS	0.70 (0.55, 0.89)	0.004	0.86 (0.67, 1.11)	0.25	1.34 (0.88, 2.04)	0.18	-	-
Mali 2015 MIS	0.81 (0.68, 0.96)	0.01	0.89 (0.75, 1.07)	0.21	-	-	-	-
Mozambique 2011 DHS	0.78 (0.58, 1.05)	0.11	0.66 (0.50, 0.88)	0.005	0.93 (0.70, 1.24)	0.64	-	-
Mozambique 2015 AIS	-	-	0.83 (0.63, 1.11)	0.21	-	-	-	-
Namibia 2006 DHS	-	-	-	-	0.69 (0.39, 1.23)	0.21	1.15 (0.25, 5.34)	0.86
Namibia 2013 DHS	-	-	-	-	0.86 (0.63, 1.18)	0.34	0.99 (0.62, 1.60)	0.98
Niger 2012 DHS	-	-	-	-	0.91 (0.69, 1.22)	0.54	1.17 (0.66, 2.08)	0.58
Nigeria 2008 DHS	-	-	-	-	0.51 (0.14, 1.79)	0.29	-	-
Nigeria 2010 MIS	1.00 (0.81, 1.25)	0.97	0.80 (0.64, 1.00)	0.05	-	-	-	-
Nigeria 2013 DHS	-	-	-	-	0.71 (0.37, 1.36)	0.30	-	-
Nigeria 2015 MIS	0.86 (0.70, 1.05)	0.14	0.91 (0.75, 1.11)	0.35	-	-	-	-
Rwanda 2010 DHS	0.65 (0.35, 1.23)	0.19	0.63 (0.39, 1.01)	0.05	1.03 (0.83, 1.28)	0.77	0.86 (0.59, 1.24)	0.41
Rwanda 2015 DHS	0.43 (0.19, 0.95)	0.04	0.31 (0.20, 0.49)	<0.001	0.88 (0.71, 1.08)	0.22	0.90 (0.66, 1.22)	0.48
Rwanda 2017 MIS	0.44 (0.29, 0.69)	<0.001	0.46 (0.32, 0.68)	<0.001	-	-	-	-
Senegal 2008 MIS	0.75 (0.48, 1.18)	0.21	1.07 (0.76, 1.51)	0.71	-	-	-	-
Senegal 2010 DHS	1.36 (0.82, 2.25)	0.23	0.83 (0.49, 1.41)	0.49	1.05 (0.85, 1.30)	0.66	1.09 (0.64, 1.83)	0.76
Senegal 2012 DHS	0.71 (0.48, 1.05)	0.08	0.74 (0.52, 1.06)	0.10	0.95 (0.70, 1.29)	0.73	-	-
Senegal 2014 DHS	0.83 (0.49, 1.39)	0.47	0.75 (0.44, 1.26)	0.27	0.71 (0.53, 0.96)	0.02	-	-
Senegal 2015 DHS	0.92 (0.35, 2.37)	0.86	1.01 (0.57, 1.78)	0.99	0.73 (0.54, 0.99)	0.04	-	-
Senegal 2016 DHS	1.18 (0.68, 2.06)	0.56	1.29 (0.80, 2.08)	0.30	1.45 (1.03, 2.03)	0.03	-	-
Sierra Leone 2008 DHS	-	-	-	-	0.62 (0.27, 1.39)	0.25	-	-
Sierra Leone 2013 DHS	-	-	-	-	0.95 (0.74, 1.23)	0.71	0.74 (0.46, 1.19)	0.21
Sierra Leone 2016 MIS	0.94 (0.83, 1.06)	0.31	0.87 (0.76, 0.99)	0.04	-	-	-	-
Tanzania 2004 DHS	-	-	-	-	1.24 (0.92, 1.68)	0.16	-	-
Tanzania 2010 DHS	-	-	-	-	0.98 (0.73, 1.30)	0.87	1.06 (0.63, 1.80)	0.82
Tanzania 2012 AIS	0.70 (0.50, 0.97)	0.03	0.66 (0.52, 0.86)	0.002	-	-	-	-
Tanzania 2017 MIS	-	-	0.71 (0.55, 0.90)	0.005	-	-	-	-
Togo 2013 DHS	1.01 (0.82, 1.25)	0.91	0.92 (0.74, 1.15)	0.47	1.12 (0.81, 1.56)	0.48	0.93 (0.51, 1.72)	0.83

Table F. Association between finished house construction materials and malaria, diarrhoea and acute respiratory infection in children aged 0-5 years in sub-Saharan Africa (crude results)

Survey	Malaria positive by microscopy		Malaria positive by RDT		Diarrhoea in past two weeks		Cough with short and rapid breathing in past two weeks	
	OR (95% CI)	p	OR (95% CI)	p	OR (95% CI)	p	OR (95% CI)	p
Uganda 2006 DHS	-	-	-	-	1.03 (0.66, 1.59)	0.90	0.55 (0.26, 1.18)	0.12
Uganda 2009 MIS	0.65 (0.52, 0.81)	<0.001	0.64 (0.51, 0.79)	<0.001	-	-	-	-
Uganda 2014 MIS	0.80 (0.63, 1.02)	0.07	0.75 (0.59, 0.94)	0.01	-	-	-	-
Uganda 2016 DHS	-	-	0.55 (0.44, 0.68)	<0.001	1.14 (0.95, 1.38)	0.16	1.14 (0.90, 1.43)	0.28
Zambia 2007 DHS	-	-	-	-	1.24 (0.73, 2.11)	0.42	0.47 (0.13, 1.65)	0.24
Zambia 2013 DHS	-	-	-	-	1.02 (0.85, 1.23)	0.82	1.40 (0.98, 2.01)	0.07
Zimbabwe 2005 DHS	-	-	-	-	1.04 (0.74, 1.47)	0.81	1.06 (0.56, 2.03)	0.85
Zimbabwe 2010 DHS	-	-	-	-	0.89 (0.65, 1.23)	0.48	1.21 (0.70, 2.10)	0.50
Zimbabwe 2015 DHS	-	-	-	-	0.98 (0.72, 1.35)	0.92	1.15 (0.74, 1.78)	0.54

AIS: AIDS Indicator Survey; CI: confidence intervals; DHS: Demographic and Health Survey; DRC: Democratic Republic of the Congo; MIS: Malaria Indicator Survey; OR: Odds Ratio; RDT: rapid diagnostic test. All p-values are calculated using the likelihood ratio test.

Table G. Association between finished house construction materials, growth failure and anaemia in children aged 0-5 years in sub-Saharan Africa (crude results)

Survey	Low height-for-age		Low weight-for-height		Low weight-for-age		Any anaemia		Moderate to severe anaemia	
	OR (95% CI)	p	OR (95% CI)	p	OR (95% CI)	p	OR (95% CI)	p	OR (95% CI)	p
Angola 2011 MIS	-	-	-	-	-	-	-	-	-	-
Angola 2015 DHS	0.87 (0.62, 1.22)	0.42	0.78 (0.37, 1.64)	0.51	0.69 (0.47, 1.02)	0.06	-	-	-	-
Benin 2001 DHS	-	-	-	-	-	-	-	-	-	-
Benin 2006 DHS	0.77 (0.67, 0.90)	<0.001	1.07 (0.81, 1.42)	0.63	0.74 (0.63, 0.88)	<0.001	0.80 (0.56, 1.14)	0.21	0.82 (0.60, 1.11)	0.20
Benin 2012 DHS	0.81 (0.68, 0.96)	0.02	0.88 (0.70, 1.10)	0.26	0.85 (0.70, 1.03)	0.09	0.99 (0.68, 1.43)	0.95	1.33 (0.92, 1.91)	0.13
Burkina Faso 2010 DHS	0.95 (0.78, 1.15)	0.59	0.86 (0.66, 1.11)	0.25	0.98 (0.80, 1.19)	0.83	0.64 (0.47, 0.87)	0.005	0.80 (0.65, 0.99)	0.04
Burkina Faso 2014 MIS	-	-	-	-	-	-	-	-	-	-
Burundi 2010 DHS	0.70 (0.54, 0.91)	0.008	0.90 (0.50, 1.62)	0.73	0.63 (0.48, 0.84)	0.001	1.06 (0.80, 1.40)	0.68	0.99 (0.70, 1.39)	0.94
Burundi 2012 MIS	-	-	-	-	-	-	-	-	-	-
Burundi 2016 DHS	0.70 (0.57, 0.85)	<0.001	1.11 (0.71, 1.71)	0.65	0.64 (0.52, 0.79)	<0.001	0.75 (0.59, 0.94)	0.01	0.69 (0.54, 0.87)	0.002
Cameroon 2011 DHS	0.70 (0.52, 0.94)	0.02	1.17 (0.59, 2.32)	0.65	0.86 (0.58, 1.29)	0.47	1.16 (0.83, 1.61)	0.40	0.84 (0.60, 1.19)	0.33
Comoros 2012 DHS	0.67 (0.44, 1.03)	0.07	0.81 (0.44, 1.49)	0.49	0.88 (0.55, 1.43)	0.61	-	-	-	-
Congo 2005 DHS	0.71 (0.54, 0.93)	0.01	1.14 (0.74, 1.77)	0.55	0.84 (0.62, 1.15)	0.28	1.04 (0.72, 1.48)	0.85	0.74 (0.52, 1.04)	0.08
Congo 2011 DHS	0.73 (0.57, 0.93)	0.01	0.83 (0.52, 1.31)	0.42	0.75 (0.57, 0.99)	0.04	0.97 (0.77, 1.23)	0.82	1.14 (0.90, 1.45)	0.28
DRC 2013 DHS	0.81 (0.64, 1.01)	0.06	1.12 (0.70, 1.78)	0.64	0.83 (0.64, 1.07)	0.14	0.96 (0.75, 1.23)	0.74	0.96 (0.75, 1.24)	0.77
Cote d'Ivoire 2012 DHS	0.92 (0.60, 1.41)	0.70	0.68 (0.33, 1.42)	0.31	0.68 (0.44, 1.06)	0.09	0.85 (0.52, 1.40)	0.52	0.97 (0.65, 1.46)	0.90
Eswatini 2006 DHS	0.57 (0.39, 0.85)	0.006	0.82 (0.25, 2.71)	0.75	0.46 (0.24, 0.86)	0.01	0.96 (0.67, 1.38)	0.83	1.03 (0.67, 1.59)	0.89
Ethiopia 2016 DHS	-	-	-	-	-	-	-	-	-	-
Gabon 2012 DHS	0.90 (0.65, 1.23)	0.51	1.37 (0.61, 3.09)	0.45	1.19 (0.78, 1.81)	0.41	1.05 (0.78, 1.41)	0.76	0.89 (0.66, 1.18)	0.42
Gambia 2013 DHS	0.60 (0.39, 0.92)	0.02	0.72 (0.41, 1.28)	0.27	0.97 (0.65, 1.46)	0.89	0.76 (0.48, 1.19)	0.23	0.90 (0.62, 1.30)	0.56
Ghana 2008 DHS	0.82 (0.34, 1.95)	0.65	0.41 (0.13, 1.34)	0.14	0.45 (0.17, 1.22)	0.12	1.23 (0.30, 5.12)	0.77	0.40 (0.12, 1.36)	0.14
Ghana 2014 DHS	0.56 (0.27, 1.13)	0.10	1.15 (0.42, 3.16)	0.79	0.68 (0.37, 1.26)	0.22	0.99 (0.52, 1.89)	0.98	0.70 (0.40, 1.24)	0.23
Ghana 2016 MIS	-	-	-	-	-	-	-	-	-	-
Guinea 2012 DHS	1.19 (0.74, 1.90)	0.48	1.24 (0.64, 2.40)	0.52	1.37 (0.85, 2.20)	0.20	0.76 (0.47, 1.22)	0.25	1.00 (0.66, 1.51)	1.00
Kenya 2008 DHS	0.76 (0.55, 1.06)	0.10	0.70 (0.38, 1.32)	0.27	0.72 (0.49, 1.05)	0.09	-	-	-	-
Kenya 2014 DHS	0.71 (0.55, 0.91)	0.007	0.89 (0.52, 1.54)	0.69	0.67 (0.50, 0.90)	0.007	-	-	-	-
Kenya 2015 MIS	-	-	-	-	-	-	-	-	-	-
Lesotho 2009 DHS	-	-	-	-	-	-	-	-	-	-
Lesotho 2014 DHS	-	-	-	-	-	-	-	-	-	-
Liberia 2011 MIS	-	-	-	-	-	-	-	-	-	-
Liberia 2013 DHS	0.54 (0.28, 1.05)	0.07	2.48 (0.77, 7.93)	0.13	0.94 (0.45, 1.95)	0.86	-	-	-	-
Liberia 2016 MIS	-	-	-	-	-	-	-	-	-	-
Madagascar 2008 DHS	0.60 (0.43, 0.85)	0.004	-	-	-	-	0.86 (0.51, 1.46)	0.58	1.99 (0.95, 4.19)	0.07
Madagascar 2011 MIS	-	-	-	-	-	-	-	-	-	-

Table G. Association between finished house construction materials, growth failure and anaemia in children aged 0-5 years in sub-Saharan Africa (crude results)

Survey	Low height-for-age		Low weight-for-height		Low weight-for-age		Any anaemia		Moderate to severe anaemia	
	OR (95% CI)	p	OR (95% CI)	p	OR (95% CI)	p	OR (95% CI)	p	OR (95% CI)	p
Madagascar 2013 MIS	-	-	-	-	-	-	-	-	-	-
Malawi 2010 DHS	0.91 (0.72, 1.15)	0.43	0.47 (0.24, 0.90)	0.02	0.76 (0.55, 1.05)	0.10	0.77 (0.61, 0.99)	0.04	0.72 (0.56, 0.93)	0.01
Malawi 2012 MIS	-	-	-	-	-	-	-	-	-	-
Malawi 2014 MIS	-	-	-	-	-	-	-	-	-	-
Malawi 2015 DHS	0.65 (0.50, 0.85)	0.001	0.99 (0.51, 1.93)	0.98	1.08 (0.80, 1.46)	0.60	0.81 (0.61, 1.07)	0.14	0.90 (0.70, 1.17)	0.45
Malawi 2017 MIS	-	-	-	-	-	-	-	-	-	-
Mali 2012 DHS	1.19 (0.71, 1.99)	0.51	2.19 (0.88, 5.46)	0.09	1.17 (0.69, 1.99)	0.56	0.84 (0.49, 1.43)	0.52	0.74 (0.47, 1.18)	0.21
Mali 2015 MIS	-	-	-	-	-	-	-	-	-	-
Mozambique 2011 DHS	0.80 (0.65, 0.99)	0.04	0.73 (0.44, 1.22)	0.23	0.72 (0.54, 0.96)	0.02	0.76 (0.56, 1.02)	0.07	0.71 (0.52, 0.97)	0.03
Mozambique 2015 AIS	-	-	-	-	-	-	-	-	-	-
Namibia 2006 DHS	0.58 (0.40, 0.83)	0.003	0.94 (0.52, 1.70)	0.84	0.83 (0.57, 1.20)	0.32	-	-	-	-
Namibia 2013 DHS	0.56 (0.32, 0.97)	0.04	0.84 (0.37, 1.90)	0.68	0.42 (0.23, 0.75)	0.003	0.92 (0.57, 1.50)	0.74	0.87 (0.50, 1.52)	0.62
Niger 2012 DHS	0.76 (0.48, 1.21)	0.25	0.51 (0.29, 0.92)	0.03	0.84 (0.54, 1.29)	0.42	0.83 (0.52, 1.33)	0.44	1.07 (0.70, 1.65)	0.76
Nigeria 2008 DHS	0.70 (0.39, 1.27)	0.24	0.80 (0.30, 2.11)	0.65	0.66 (0.35, 1.24)	0.20	-	-	-	-
Nigeria 2010 MIS	-	-	-	-	-	-	-	-	-	-
Nigeria 2013 DHS	0.60 (0.35, 1.01)	0.06	0.82 (0.46, 1.45)	0.49	0.48 (0.29, 0.79)	0.004	-	-	-	-
Nigeria 2015 MIS	-	-	-	-	-	-	-	-	-	-
Rwanda 2010 DHS	0.61 (0.48, 0.77)	<0.001	0.60 (0.29, 1.24)	0.17	0.57 (0.41, 0.79)	<0.001	0.81 (0.63, 1.03)	0.08	0.79 (0.57, 1.09)	0.15
Rwanda 2015 DHS	0.64 (0.51, 0.81)	<0.001	0.62 (0.27, 1.42)	0.26	0.51 (0.36, 0.72)	<0.001	0.86 (0.69, 1.08)	0.20	0.67 (0.49, 0.92)	0.01
Rwanda 2017 MIS	-	-	-	-	-	-	-	-	-	-
Senegal 2008 MIS	-	-	-	-	-	-	-	-	-	-
Senegal 2010 DHS	0.84 (0.56, 1.26)	0.41	1.72 (0.87, 3.40)	0.12	0.86 (0.56, 1.30)	0.47	0.93 (0.59, 1.48)	0.77	0.95 (0.65, 1.38)	0.77
Senegal 2012 DHS	0.95 (0.69, 1.30)	0.74	0.78 (0.52, 1.18)	0.24	0.69 (0.52, 0.91)	0.009	0.84 (0.63, 1.12)	0.24	0.90 (0.69, 1.17)	0.42
Senegal 2014 DHS	0.80 (0.57, 1.13)	0.20	0.73 (0.43, 1.23)	0.24	0.78 (0.57, 1.08)	0.13	1.07 (0.80, 1.43)	0.65	1.06 (0.79, 1.41)	0.71
Senegal 2015 DHS	0.70 (0.49, 0.98)	0.04	0.84 (0.53, 1.34)	0.46	0.80 (0.59, 1.09)	0.16	1.10 (0.80, 1.52)	0.54	0.96 (0.72, 1.28)	0.80
Senegal 2016 DHS	1.04 (0.70, 1.53)	0.86	1.34 (0.81, 2.22)	0.25	1.06 (0.76, 1.48)	0.74	0.75 (0.54, 1.05)	0.10	0.73 (0.55, 0.98)	0.03
Sierra Leone 2008 DHS	0.63 (0.36, 1.11)	0.11	0.64 (0.21, 1.95)	0.43	0.68 (0.36, 1.27)	0.23	0.61 (0.19, 2.00)	0.41	0.85 (0.31, 2.34)	0.76
Sierra Leone 2013 DHS	1.19 (0.88, 1.61)	0.26	0.67 (0.38, 1.18)	0.17	1.06 (0.75, 1.51)	0.73	0.97 (0.65, 1.45)	0.88	1.05 (0.77, 1.43)	0.77
Sierra Leone 2016 MIS	-	-	-	-	-	-	-	-	-	-
Tanzania 2004 DHS	0.69 (0.54, 0.88)	0.003	0.96 (0.50, 1.83)	0.90	0.66 (0.50, 0.87)	0.004	0.79 (0.63, 1.00)	0.05	0.65 (0.52, 0.82)	<0.001
Tanzania 2010 DHS	0.87 (0.70, 1.08)	0.21	1.05 (0.64, 1.71)	0.85	1.00 (0.77, 1.31)	0.98	1.18 (0.96, 1.46)	0.11	1.16 (0.92, 1.47)	0.22
Tanzania 2012 AIS	-	-	-	-	-	-	-	-	-	-
Tanzania 2017 MIS	-	-	-	-	-	-	-	-	-	-
Togo 2013 DHS	0.58 (0.38, 0.88)	0.01	0.53 (0.26, 1.09)	0.09	0.67 (0.43, 1.05)	0.08	0.90 (0.59, 1.37)	0.62	0.80 (0.54, 1.19)	0.27

Table G. Association between finished house construction materials, growth failure and anaemia in children aged 0-5 years in sub-Saharan Africa (crude results)

Survey	Low height-for-age		Low weight-for-height		Low weight-for-age		Any anaemia		Moderate to severe anaemia	
	**OR (95% CI)**	**p**	**OR (95% CI)**	**p**	**OR (95% CI)**	**p**	**OR (95% CI)**	**p**	**OR (95% CI)**	**p**
Uganda 2006 DHS	0.81 (0.42, 1.56)	0.53	0.39 (0.11, 1.34)	0.13	0.65 (0.29, 1.44)	0.28	0.28 (0.09, 0.83)	0.02	0.37 (0.14, 0.97)	0.04
Uganda 2009 MIS	-	-	-	-	-	-	-	-	-	-
Uganda 2014 MIS	-	-	-	-	-	-	-	-	-	-
Uganda 2016 DHS	0.53 (0.33, 0.86)	0.01	0.91 (0.38, 2.19)	0.84	0.74 (0.43, 1.27)	0.27	0.68 (0.46, 1.00)	0.05	0.58 (0.38, 0.90)	0.01
Zambia 2007 DHS	0.77 (0.58, 1.03)	0.08	1.03 (0.58, 1.83)	0.93	0.67 (0.47, 0.96)	0.03	-	-	-	-
Zambia 2013 DHS	0.85 (0.73, 0.98)	0.03	0.85 (0.62, 1.18)	0.33	0.72 (0.60, 0.87)	<0.001	-	-	-	-
Zimbabwe 2005 DHS	0.85 (0.65, 1.11)	0.24	1.19 (0.71, 1.99)	0.52	0.89 (0.64, 1.25)	0.50	1.02 (0.78, 1.34)	0.88	1.18 (0.88, 1.58)	0.28
Zimbabwe 2010 DHS	0.73 (0.57, 0.94)	0.02	0.81 (0.42, 1.57)	0.54	0.71 (0.52, 0.98)	0.04	0.89 (0.70, 1.15)	0.38	0.93 (0.71, 1.22)	0.60
Zimbabwe 2015 DHS	0.81 (0.59, 1.13)	0.21	1.09 (0.60, 1.98)	0.78	0.70 (0.47, 1.04)	0.08	0.96 (0.70, 1.31)	0.79	1.48 (1.00, 2.19)	0.05

AIS: AIDS Indicator Survey; CI: confidence intervals; DHS: Demographic and Health Survey; DRC: Democratic Republic of the Congo; MIS: Malaria Indicator Survey; OR: Odds Ratio. All p-values are calculated using the likelihood ratio test.

Table H. Association between improved housing and malaria, diarrhoea and acute respiratory infection in children aged 0-5 years in sub-Saharan Africa (crude results)

Survey	Malaria positive by microscopy		Malaria positive by RDT		Diarrhoea in past two weeks		Cough with short and rapid breathing in past two weeks	
	OR (95% CI)	p	OR (95% CI)	p	OR (95% CI)	p	OR (95% CI)	p
Angola 2011 MIS	2.13 (0.79, 5.77)	0.14	0.17 (0.02, 1.35)	0.09	-	-	-	-
Angola 2015 DHS	-	-	0.69 (0.46, 1.03)	0.07	-	-	-	-
Benin 2001 DHS	-	-	-	-	-	-	-	-
Benin 2006 DHS	-	-	-	-	0.94 (0.63, 1.39)	0.75	1.43 (0.82, 2.51)	0.21
Benin 2012 DHS	0.69 (0.45, 1.07)	0.10	0.61 (0.38, 0.99)	0.04	1.01 (0.68, 1.52)	0.95	-	-
Burkina Faso 2010 DHS	0.79 (0.61, 1.01)	0.06	0.89 (0.69, 1.15)	0.37	0.96 (0.79, 1.18)	0.72	-	-
Burkina Faso 2014 MIS	0.66 (0.52, 0.83)	<0.001	0.71 (0.56, 0.89)	0.003	-	-	-	-
Burundi 2010 DHS	-	-	-	-	0.85 (0.66, 1.11)	0.23	1.12 (0.69, 1.83)	0.65
Burundi 2012 MIS	0.68 (0.45, 1.05)	0.08	0.55 (0.37, 0.84)	0.005	-	-	-	-
Burundi 2016 DHS	0.89 (0.73, 1.09)	0.28	0.71 (0.58, 0.87)	<0.001	0.95 (0.80, 1.12)	0.53	0.86 (0.69, 1.07)	0.18
Cameroon 2011 DHS	-	-	0.80 (0.66, 0.98)	0.03	0.67 (0.52, 0.86)	0.002	1.07 (0.77, 1.47)	0.70
Comoros 2012 DHS	-	-	-	-	1.22 (0.81, 1.85)	0.34	7.34 (2.17, 24.78)	0.001
Congo 2005 DHS	-	-	-	-	0.80 (0.50, 1.26)	0.33	0.80 (0.39, 1.64)	0.55
Congo 2011 DHS	-	-	-	-	0.76 (0.57, 1.01)	0.06	1.17 (0.73, 1.88)	0.51
DRC 2013 DHS	0.50 (0.31, 0.83)	0.01	0.63 (0.41, 0.99)	0.04	1.18 (0.90, 1.56)	0.23	0.93 (0.62, 1.40)	0.74
Cote d'Ivoire 2012 DHS	0.65 (0.45, 0.95)	0.03	0.69 (0.54, 0.90)	0.01	0.79 (0.61, 1.03)	0.08	0.60 (0.34, 1.05)	0.07
Eswatini 2006 DHS	-	-	-	-	0.80 (0.45, 1.43)	0.45	1.05 (0.47, 2.35)	0.90
Ethiopia 2016 DHS	-	-	-	-	0.49 (0.23, 1.05)	0.07	0.10 (0.01, 0.82)	0.03
Gabon 2012 DHS	-	-	-	-	0.78 (0.61, 0.99)	0.04	1.30 (0.93, 1.81)	0.13
Gambia 2013 DHS	-	-	0.55 (0.21, 1.46)	0.23	1.09 (0.89, 1.33)	0.41	-	-
Ghana 2008 DHS	-	-	-	-	0.76 (0.31, 1.90)	0.56	-	-
Ghana 2014 DHS	0.75 (0.54, 1.05)	0.09	0.99 (0.72, 1.37)	0.96	0.94 (0.62, 1.42)	0.76	0.66 (0.21, 2.12)	0.49
Ghana 2016 MIS	1.14 (0.80, 1.64)	0.47	0.71 (0.49, 1.03)	0.07	-	-	-	-
Guinea 2012 DHS	1.04 (0.78, 1.39)	0.77	0.84 (0.63, 1.13)	0.25	0.90 (0.68, 1.19)	0.47	0.64 (0.35, 1.17)	0.15
Kenya 2008 DHS	-	-	-	-	1.14 (0.63, 2.08)	0.66	1.09 (0.31, 3.88)	0.89
Kenya 2014 DHS	-	-	-	-	0.71 (0.50, 1.01)	0.06	2.17 (1.23, 3.82)	0.01
Kenya 2015 MIS	-	-	0.31 (0.13, 0.78)	0.01	-	-	-	-
Lesotho 2009 DHS	-	-	-	-	1.45 (0.44, 4.74)	0.54	-	-
Lesotho 2014 DHS	-	-	-	-	0.49 (0.30, 0.80)	0.005	0.73 (0.36, 1.49)	0.39
Liberia 2011 MIS	1.24 (0.76, 2.05)	0.39	0.79 (0.50, 1.25)	0.31	-	-	-	-
Liberia 2013 DHS	-	-	-	-	0.79 (0.44, 1.41)	0.42	0.51 (0.18, 1.41)	0.19
Liberia 2016 MIS	-	-	0.73 (0.49, 1.07)	0.10	-	-	-	-
Madagascar 2008 DHS	-	-	-	-	0.60 (0.23, 1.56)	0.30	-	-

Table H. Association between improved housing and malaria, diarrhoea and acute respiratory infection in children aged 0-5 years in sub-Saharan Africa (crude results)

Survey	Malaria positive by microscopy		Malaria positive by RDT		Diarrhoea in past two weeks		Cough with short and rapid breathing in past two weeks	
	**OR (95% CI)**	**p**	**OR (95% CI)**	**p**	**OR (95% CI)**	**p**	**OR (95% CI)**	**p**
Madagascar 2011 MIS	-	-	-	-	-	-	-	-
Madagascar 2013 MIS	3.37 (0.59, 19.21)	0.17	2.54 (0.46, 13.90)	0.28	-	-	-	-
Malawi 2010 DHS	-	-	-	-	0.87 (0.65, 1.18)	0.37	0.84 (0.52, 1.36)	0.48
Malawi 2012 MIS	0.25 (0.08, 0.73)	0.01	0.27 (0.12, 0.62)	0.002	-	-	-	-
Malawi 2014 MIS	0.82 (0.36, 1.87)	0.64	0.72 (0.33, 1.55)	0.40	-	-	-	-
Malawi 2015 DHS	-	-	-	-	0.88 (0.76, 1.01)	0.08	0.84 (0.68, 1.02)	0.08
Malawi 2017 MIS	0.32 (0.15, 0.66)	0.002	0.43 (0.24, 0.76)	0.004	-	-	-	-
Mali 2012 DHS	0.73 (0.54, 0.99)	0.04	0.86 (0.61, 1.21)	0.39	1.20 (0.86, 1.67)	0.28	-	-
Mali 2015 MIS	0.84 (0.67, 1.05)	0.12	0.68 (0.52, 0.89)	0.01	-	-	-	-
Mozambique 2011 DHS	0.39 (0.21, 0.73)	0.003	0.47 (0.26, 0.83)	0.01	1.03 (0.74, 1.44)	0.86	-	-
Mozambique 2015 AIS	-	-	0.75 (0.40, 1.38)	0.35	-	-	-	-
Namibia 2006 DHS	-	-	-	-	0.83 (0.50, 1.36)	0.46	1.27 (0.35, 4.54)	0.71
Namibia 2013 DHS	-	-	-	-	0.96 (0.69, 1.34)	0.81	0.51 (0.31, 0.86)	0.01
Niger 2012 DHS	-	-	-	-	0.96 (0.70, 1.31)	0.78	1.39 (0.78, 2.49)	0.26
Nigeria 2008 DHS	-	-	-	-	0.57 (0.26, 1.24)	0.16	-	-
Nigeria 2010 MIS	1.01 (0.78, 1.31)	0.94	0.71 (0.54, 0.92)	0.01	-	-	-	-
Nigeria 2013 DHS	-	-	-	-	0.93 (0.67, 1.29)	0.65	-	-
Nigeria 2015 MIS	0.94 (0.71, 1.25)	0.69	0.86 (0.69, 1.08)	0.20	-	-	-	-
Rwanda 2010 DHS	1.43 (0.62, 3.33)	0.40	1.09 (0.55, 2.16)	0.81	0.98 (0.78, 1.25)	0.90	0.93 (0.62, 1.42)	0.75
Rwanda 2015 DHS	0.31 (0.07, 1.42)	0.13	0.35 (0.17, 0.73)	0.005	0.66 (0.51, 0.87)	0.003	0.79 (0.55, 1.13)	0.19
Rwanda 2017 MIS	0.41 (0.24, 0.70)	0.001	0.41 (0.26, 0.64)	<0.001	-	-	-	-
Senegal 2008 MIS	0.62 (0.26, 1.47)	0.28	0.89 (0.57, 1.40)	0.62	-	-	-	-
Senegal 2010 DHS	0.91 (0.45, 1.85)	0.80	0.84 (0.42, 1.66)	0.62	1.04 (0.87, 1.26)	0.65	0.99 (0.68, 1.45)	0.96
Senegal 2012 DHS	1.52 (0.76, 3.04)	0.23	0.96 (0.52, 1.77)	0.89	0.79 (0.58, 1.08)	0.14	-	-
Senegal 2014 DHS	0.47 (0.08, 2.87)	0.41	1.18 (0.30, 4.68)	0.81	0.77 (0.59, 1.00)	0.05	-	-
Senegal 2015 DHS	0.33 (0.07, 1.58)	0.17	0.88 (0.17, 4.44)	0.88	0.92 (0.72, 1.19)	0.55	-	-
Senegal 2016 DHS	1.12 (0.29, 4.39)	0.87	1.51 (0.39, 5.80)	0.55	0.93 (0.69, 1.25)	0.62	-	-
Sierra Leone 2008 DHS	-	-	-	-	0.82 (0.31, 2.19)	0.70	-	-
Sierra Leone 2013 DHS	-	-	-	-	0.94 (0.68, 1.29)	0.68	1.01 (0.60, 1.69)	0.98
Sierra Leone 2016 MIS	0.96 (0.81, 1.14)	0.66	0.96 (0.81, 1.15)	0.68	-	-	-	-
Tanzania 2004 DHS	-	-	-	-	-	-	-	-
Tanzania 2010 DHS	-	-	-	-	0.89 (0.60, 1.31)	0.56	0.69 (0.32, 1.46)	0.33
Tanzania 2012 AIS	0.70 (0.27, 1.81)	0.46	0.94 (0.50, 1.77)	0.84	-	-	-	-

Table H. Association between improved housing and malaria, diarrhoea and acute respiratory infection in children aged 0-5 years in sub-Saharan Africa (crude results)

Survey	Malaria positive by microscopy		Malaria positive by RDT		Diarrhoea in past two weeks		Cough with short and rapid breathing in past two weeks	
	**OR (95% CI)**	**p**	**OR (95% CI)**	**p**	**OR (95% CI)**	**p**	**OR (95% CI)**	**p**
Tanzania 2017 MIS	-	-	0.53 (0.31, 0.88)	0.02	-	-	-	-
Togo 2013 DHS	0.54 (0.36, 0.79)	0.002	0.60 (0.41, 0.89)	0.01	0.81 (0.56, 1.18)	0.28	0.99 (0.60, 1.65)	0.98
Uganda 2006 DHS	-	-	-	-	0.76 (0.34, 1.67)	0.49	0.44 (0.13, 1.52)	0.19
Uganda 2009 MIS	0.57 (0.38, 0.86)	0.01	0.61 (0.42, 0.90)	0.01	-	-	-	-
Uganda 2014 MIS	0.66 (0.38, 1.15)	0.14	0.73 (0.46, 1.16)	0.18	-	-	-	-
Uganda 2016 DHS	-	-	0.66 (0.45, 0.96)	0.03	0.79 (0.60, 1.03)	0.08	0.92 (0.66, 1.28)	0.62
Zambia 2007 DHS	-	-	-	-	1.22 (0.72, 2.09)	0.46	0.79 (0.19, 3.29)	0.74
Zambia 2013 DHS	-	-	-	-	0.79 (0.64, 0.99)	0.04	1.24 (0.80, 1.91)	0.33
Zimbabwe 2005 DHS	-	-	-	-	1.24 (0.89, 1.73)	0.20	0.68 (0.35, 1.32)	0.26
Zimbabwe 2010 DHS	-	-	-	-	0.91 (0.68, 1.21)	0.51	1.11 (0.61, 2.02)	0.74
Zimbabwe 2015 DHS	-	-	-	-	0.69 (0.53, 0.90)	0.01	0.78 (0.52, 1.17)	0.23

AIS: AIDS Indicator Survey; CI: confidence intervals; DHS: Demographic and Health Survey; DRC: Democratic Republic of the Congo; MIS: Malaria Indicator Survey; OR: Odds Ratio; RDT: rapid diagnostic test. All p-values are calculated using the likelihood ratio test.

Table I. Association between improved housing, growth failure and anaemia in children aged 0-5 years in sub-Saharan Africa (crude results)

Survey	Low height-for-age		Low weight-for-height		Low weight-for-age		Any anaemia		Moderate to severe anaemia	
	OR (95% CI)	p	OR (95% CI)	p	OR (95% CI)	p	OR (95% CI)	p	OR (95% CI)	p
Angola 2011 MIS	-	-	-	-	-	-	-	-	-	-
Angola 2015 DHS	0.74 (0.47, 1.15)	0.18	0.71 (0.30, 1.67)	0.43	0.75 (0.47, 1.19)	0.23	-	-	-	-
Benin 2001 DHS	-	-	-	-	-	-	-	-	-	-
Benin 2006 DHS	0.88 (0.69, 1.14)	0.34	0.60 (0.36, 1.02)	0.06	0.87 (0.64, 1.18)	0.37	0.67 (0.40, 1.11)	0.12	0.61 (0.37, 1.00)	0.05
Benin 2012 DHS	0.61 (0.47, 0.79)	<0.001	0.85 (0.59, 1.23)	0.39	0.70 (0.52, 0.96)	0.02	0.97 (0.57, 1.65)	0.90	1.16 (0.63, 2.11)	0.63
Burkina Faso 2010 DHS	0.76 (0.57, 1.02)	0.06	0.83 (0.58, 1.20)	0.33	0.73 (0.55, 0.97)	0.03	0.85 (0.62, 1.17)	0.32	0.87 (0.67, 1.12)	0.27
Burkina Faso 2014 MIS	-	-	-	-	-	-	-	-	-	-
Burundi 2010 DHS	0.60 (0.41, 0.87)	0.01	0.64 (0.27, 1.50)	0.30	0.62 (0.41, 0.94)	0.02	0.79 (0.54, 1.16)	0.22	0.70 (0.42, 1.17)	0.17
Burundi 2012 MIS	-	-	-	-	-	-	-	-	-	-
Burundi 2016 DHS	0.70 (0.54, 0.89)	0.004	0.90 (0.52, 1.56)	0.70	0.67 (0.51, 0.86)	0.002	0.70 (0.54, 0.90)	0.01	0.76 (0.57, 1.00)	0.05
Cameroon 2011 DHS	0.81 (0.60, 1.10)	0.18	1.15 (0.56, 2.38)	0.70	1.06 (0.71, 1.58)	0.78	0.84 (0.64, 1.11)	0.23	0.90 (0.66, 1.23)	0.49
Comoros 2012 DHS	0.81 (0.53, 1.25)	0.35	0.76 (0.43, 1.34)	0.34	0.98 (0.61, 1.59)	0.94	-	-	-	-
Congo 2005 DHS	0.67 (0.43, 1.04)	0.07	0.76 (0.37, 1.56)	0.46	0.66 (0.35, 1.27)	0.22	0.65 (0.42, 0.99)	0.05	0.51 (0.30, 0.85)	0.01
Congo 2011 DHS	0.65 (0.39, 1.08)	0.10	1.11 (0.57, 2.15)	0.76	0.74 (0.44, 1.24)	0.26	0.93 (0.65, 1.32)	0.67	0.65 (0.45, 0.94)	0.02
DRC 2013 DHS	0.73 (0.49, 1.09)	0.13	1.24 (0.60, 2.55)	0.56	0.56 (0.33, 0.94)	0.03	0.88 (0.62, 1.24)	0.46	0.78 (0.53, 1.16)	0.22
Cote d'Ivoire 2012 DHS	0.69 (0.45, 1.04)	0.08	0.49 (0.23, 1.01)	0.05	0.66 (0.42, 1.02)	0.06	0.99 (0.69, 1.41)	0.95	0.86 (0.61, 1.19)	0.36
Eswatini 2006 DHS	0.49 (0.29, 0.85)	0.01	0.91 (0.23, 3.59)	0.90	0.23 (0.07, 0.79)	0.02	0.52 (0.33, 0.80)	0.003	0.67 (0.39, 1.17)	0.16
Ethiopia 2016 DHS	-	-	-	-	-	-	-	-	-	-
Gabon 2012 DHS	0.62 (0.43, 0.88)	0.01	1.03 (0.56, 1.87)	0.93	0.67 (0.42, 1.05)	0.08	0.86 (0.67, 1.11)	0.24	0.82 (0.62, 1.09)	0.17
Gambia 2013 DHS	0.97 (0.65, 1.45)	0.89	0.73 (0.44, 1.21)	0.22	0.85 (0.58, 1.24)	0.39	0.64 (0.46, 0.89)	0.01	0.80 (0.59, 1.08)	0.15
Ghana 2008 DHS	1.10 (0.55, 2.21)	0.78	1.72 (0.62, 4.74)	0.30	1.38 (0.64, 3.01)	0.41	1.40 (0.64, 3.07)	0.40	1.24 (0.60, 2.55)	0.56
Ghana 2014 DHS	0.68 (0.35, 1.30)	0.24	0.82 (0.34, 1.98)	0.67	0.56 (0.31, 1.02)	0.06	0.88 (0.56, 1.38)	0.58	0.75 (0.47, 1.19)	0.22
Ghana 2016 MIS	-	-	-	-	-	-	-	-	-	-
Guinea 2012 DHS	0.99 (0.65, 1.51)	0.96	1.58 (0.90, 2.80)	0.11	0.90 (0.57, 1.40)	0.63	0.78 (0.54, 1.13)	0.18	0.87 (0.63, 1.21)	0.42
Kenya 2008 DHS	0.74 (0.50, 1.11)	0.15	1.04 (0.52, 2.08)	0.91	0.69 (0.42, 1.13)	0.14	-	-	-	-
Kenya 2014 DHS	0.53 (0.37, 0.77)	<0.001	0.74 (0.35, 1.57)	0.43	0.69 (0.45, 1.05)	0.08	-	-	-	-
Kenya 2015 MIS	-	-	-	-	-	-	-	-	-	-
Lesotho 2009 DHS	-	-	-	-	-	-	-	-	-	-
Lesotho 2014 DHS	-	-	-	-	-	-	-	-	-	-
Liberia 2011 MIS	-	-	-	-	-	-	-	-	-	-
Liberia 2013 DHS	0.73 (0.25, 2.12)	0.56	1.20 (0.20, 7.09)	0.84	2.04 (0.58, 7.16)	0.27	-	-	-	-
Liberia 2016 MIS	-	-	-	-	-	-	-	-	-	-
Madagascar 2008 DHS	0.17 (0.05, 0.52)	0.002	-	-	-	-	1.35 (0.43, 4.28)	0.61	1.82 (0.47, 7.08)	0.39
Madagascar 2011 MIS	-	-	-	-	-	-	-	-	-	-

Table I. Association between improved housing, growth failure and anaemia in children aged 0-5 years in sub-Saharan Africa (crude results)

Survey	Low height-for-age		Low weight-for-height		Low weight-for-age		Any anaemia		Moderate to severe anaemia	
	**OR (95% CI)**	**p**	**OR (95% CI)**	**p**	**OR (95% CI)**	**p**	**OR (95% CI)**	**p**	**OR (95% CI)**	**p**
Madagascar 2013 MIS	-	-	-	-	-	-	-	-	-	-
Malawi 2010 DHS	0.63 (0.35, 1.14)	0.13	0.97 (0.23, 3.97)	0.96	1.02 (0.46, 2.27)	0.95	0.81 (0.45, 1.44)	0.47	0.90 (0.46, 1.76)	0.76
Malawi 2012 MIS	-	-	-	-	-	-	-	-	-	-
Malawi 2014 MIS	-	-	-	-	-	-	-	-	-	-
Malawi 2015 DHS	0.65 (0.47, 0.90)	0.01	1.33 (0.64, 2.78)	0.45	1.16 (0.80, 1.68)	0.43	1.21 (0.87, 1.69)	0.26	0.94 (0.70, 1.27)	0.70
Malawi 2017 MIS	-	-	-	-	-	-	-	-	-	-
Mali 2012 DHS	0.96 (0.62, 1.49)	0.85	0.55 (0.30, 1.03)	0.06	0.87 (0.57, 1.33)	0.52	0.77 (0.53, 1.11)	0.16	0.88 (0.61, 1.26)	0.48
Mali 2015 MIS	-	-	-	-	-	-	-	-	-	-
Mozambique 2011 DHS	0.92 (0.71, 1.20)	0.55	1.01 (0.55, 1.86)	0.98	0.81 (0.56, 1.18)	0.27	0.88 (0.62, 1.25)	0.48	1.09 (0.74, 1.61)	0.66
Mozambique 2015 AIS	-	-	-	-	-	-	-	-	-	-
Namibia 2006 DHS	0.54 (0.37, 0.78)	0.001	0.69 (0.36, 1.30)	0.25	0.59 (0.40, 0.87)	0.01	-	-	-	-
Namibia 2013 DHS	0.38 (0.19, 0.77)	0.01	0.94 (0.44, 2.02)	0.88	0.64 (0.35, 1.18)	0.15	0.76 (0.47, 1.24)	0.27	0.91 (0.51, 1.62)	0.76
Niger 2012 DHS	0.81 (0.47, 1.40)	0.46	1.43 (0.73, 2.77)	0.29	0.86 (0.52, 1.41)	0.55	0.89 (0.54, 1.49)	0.67	1.00 (0.62, 1.63)	0.99
Nigeria 2008 DHS	1.17 (0.88, 1.54)	0.27	1.05 (0.69, 1.59)	0.83	0.97 (0.68, 1.37)	0.85	-	-	-	-
Nigeria 2010 MIS	-	-	-	-	-	-	-	-	-	-
Nigeria 2013 DHS	0.81 (0.62, 1.06)	0.12	0.69 (0.53, 0.91)	0.01	0.79 (0.61, 1.02)	0.08	-	-	-	-
Nigeria 2015 MIS	-	-	-	-	-	-	-	-	-	-
Rwanda 2010 DHS	0.54 (0.41, 0.71)	<0.001	1.15 (0.54, 2.43)	0.71	0.50 (0.33, 0.74)	<0.001	0.85 (0.65, 1.12)	0.26	0.87 (0.60, 1.28)	0.49
Rwanda 2015 DHS	0.58 (0.43, 0.79)	<0.001	0.54 (0.21, 1.39)	0.20	0.51 (0.31, 0.82)	0.01	0.58 (0.43, 0.76)	<0.001	0.57 (0.37, 0.87)	0.01
Rwanda 2017 MIS	-	-	-	-	-	-	-	-	-	-
Senegal 2008 MIS	-	-	-	-	-	-	-	-	-	-
Senegal 2010 DHS	1.11 (0.75, 1.64)	0.59	0.88 (0.54, 1.46)	0.63	1.01 (0.70, 1.46)	0.97	0.77 (0.55, 1.09)	0.14	0.87 (0.64, 1.18)	0.37
Senegal 2012 DHS	0.76 (0.54, 1.08)	0.12	0.80 (0.52, 1.21)	0.28	0.78 (0.59, 1.04)	0.09	0.80 (0.63, 1.02)	0.07	0.89 (0.71, 1.11)	0.31
Senegal 2014 DHS	0.57 (0.39, 0.85)	0.01	1.00 (0.64, 1.57)	0.99	0.76 (0.56, 1.04)	0.09	0.85 (0.67, 1.07)	0.17	0.70 (0.54, 0.92)	0.01
Senegal 2015 DHS	0.83 (0.61, 1.13)	0.23	1.01 (0.68, 1.49)	0.97	0.77 (0.59, 1.00)	0.05	0.85 (0.68, 1.06)	0.16	0.86 (0.68, 1.07)	0.18
Senegal 2016 DHS	0.79 (0.53, 1.17)	0.24	0.67 (0.43, 1.06)	0.09	0.68 (0.50, 0.93)	0.02	0.87 (0.68, 1.11)	0.25	0.84 (0.66, 1.08)	0.18
Sierra Leone 2008 DHS	0.49 (0.23, 1.04)	0.06	0.68 (0.22, 2.16)	0.52	0.49 (0.20, 1.18)	0.11	1.24 (0.37, 4.14)	0.73	0.99 (0.29, 3.30)	0.98
Sierra Leone 2013 DHS	0.96 (0.65, 1.43)	0.86	1.08 (0.54, 2.18)	0.82	1.28 (0.80, 2.05)	0.31	1.00 (0.65, 1.54)	1.00	1.08 (0.74, 1.57)	0.70
Sierra Leone 2016 MIS	-	-	-	-	-	-	-	-	-	-
Tanzania 2004 DHS	-	-	-	-	-	-	-	-	-	-
Tanzania 2010 DHS	0.65 (0.45, 0.92)	0.02	0.68 (0.38, 1.24)	0.21	0.75 (0.50, 1.13)	0.17	0.88 (0.66, 1.16)	0.36	0.86 (0.62, 1.18)	0.35
Tanzania 2012 AIS	-	-	-	-	-	-	-	-	-	-
Tanzania 2017 MIS	-	-	-	-	-	-	-	-	-	-
Togo 2013 DHS	0.79 (0.47, 1.31)	0.36	1.44 (0.69, 2.98)	0.33	0.69 (0.42, 1.14)	0.15	0.67 (0.46, 0.97)	0.03	0.58 (0.40, 0.86)	0.01

Table I. Association between improved housing, growth failure and anaemia in children aged 0-5 years in sub-Saharan Africa (crude results)

Survey	Low height-for-age		Low weight-for-height		Low weight-for-age		Any anaemia		Moderate to severe anaemia	
	OR (95% CI)	p	OR (95% CI)	p	OR (95% CI)	p	OR (95% CI)	p	OR (95% CI)	p
Uganda 2006 DHS	0.86 (0.30, 2.48)	0.78	0.26 (0.03, 2.41)	0.24	0.43 (0.09, 2.15)	0.30	0.22 (0.07, 0.72)	0.01	0.45 (0.13, 1.58)	0.21
Uganda 2009 MIS	-	-	-	-	-	-	-	-	-	-
Uganda 2014 MIS	-	-	-	-	-	-	-	-	-	-
Uganda 2016 DHS	0.42 (0.18, 0.97)	0.04	1.79 (0.38, 8.52)	0.46	0.47 (0.19, 1.15)	0.10	1.04 (0.60, 1.77)	0.90	1.00 (0.57, 1.74)	1.00
Zambia 2007 DHS	0.70 (0.48, 1.02)	0.06	1.13 (0.54, 2.36)	0.74	0.75 (0.46, 1.21)	0.24	-	-	-	-
Zambia 2013 DHS	0.88 (0.73, 1.06)	0.18	0.75 (0.52, 1.10)	0.14	0.77 (0.61, 0.98)	0.03	-	-	-	-
Zimbabwe 2005 DHS	0.94 (0.73, 1.22)	0.66	0.85 (0.51, 1.41)	0.52	0.81 (0.57, 1.15)	0.24	0.86 (0.67, 1.12)	0.27	0.97 (0.74, 1.27)	0.82
Zimbabwe 2010 DHS	0.73 (0.56, 0.94)	0.01	1.13 (0.63, 2.03)	0.69	0.80 (0.57, 1.12)	0.19	0.85 (0.67, 1.09)	0.20	0.94 (0.72, 1.23)	0.67
Zimbabwe 2015 DHS	0.76 (0.57, 1.01)	0.05	0.54 (0.29, 1.04)	0.06	0.72 (0.49, 1.06)	0.10	1.03 (0.80, 1.31)	0.82	1.16 (0.87, 1.55)	0.32

AIS: AIDS Indicator Survey; CI: confidence intervals; DHS: Demographic and Health Survey; DRC: Democratic Republic of the Congo; MIS: Malaria Indicator Survey; OR: Odds Ratio. All p-values are calculated using the likelihood ratio test.
